# Promoting Electrochemical Reactions with Dual‐Atom Catalysts for High‐Rate Lithium–Sulfur Batteries

**DOI:** 10.1002/adma.202511345

**Published:** 2025-09-22

**Authors:** Jing Yu, Oleg Usoltsev, Irina Martynova, Chen Huang, Zhifu Liang, Ivan Pinto‐Huguet, Canhuang Li, Liqiang Lu, Chaoqi Zhang, Xuan Lu, Kapil Gupta, Marc Botifoll, Laura Simonelli, François Fauth, Jin Yuan Zhou, Jordi Llorca, Yan Lu, Chao Yue Zhang, Jordi Arbiol, Andreu Cabot

**Affiliations:** ^1^ Catalonia Institute for Energy Research (IREC) Sant Adrià de Besòs Barcelona Catalonia 08930 Spain; ^2^ Catalan Institute of Nanoscience and Nanotechnology (ICN2), CSIC and BIST Campus UAB Bellaterra Barcelona Catalonia 08193 Spain; ^3^ CELLS‐ALBA Synchrotron Cerdanyola del Vallès Carrer de la Llum, 2, 26 Barcelona Catalonia 08290 Spain; ^4^ Department of Chemistry University of Barcelona Barcelona Catalonia 08028 Spain; ^5^ College of Ocean Science and Engineering Shanghai Maritime University Shanghai 201306 China; ^6^ Helmholtz‐Zentrum Berlin für Materialien und Energie Hahn‐Meitner‐Platz 1 14109 Berlin Germany; ^7^ College of Materials Science and Engineering Fuzhou University No.2, Xueyuan Road, Minhou County Fuzhou City Fujian 350108 China; ^8^ School of Physical Science and Technology Lanzhou University Lanzhou Gansu 730000 China; ^9^ Department of Chemical Engineering and Barcelona Research Center in Multiscale Science and Engineering Universitat Politècnica de Catalunya EEBE, Eduard Maristany 10–14 Barcelona Catalonia 08019 Spain; ^10^ Department of Chemistry and Biochemistry University of California Los Angeles CA 90095 USA; ^11^ ICREA Pg. Lluis Companys Barcelona Catalonia 08010 Spain

**Keywords:** carbon nitride, dual‐atom catalysts, electrocatalysts, lithium sulfide nucleation, lithium–sulfur batteries, polysulfides, single‐atom catalysts, sulfur cathodes, sulfur reduction reaction

## Abstract

Sulfur cathodes offer a promising solution for high‐energy‐density, cost‐effective, and sustainable energy storage. However, their practical application is limited by sluggish and complex multistep sulfur redox reactions (SRRs), involving both electrochemical and chemical processes. Herein, it is demonstrated that accelerating electrochemical processes, particularly Li_2_S nucleation, over competing chemical pathways is fundamental to minimizing sulfur loss and achieving high‐rate performance. To this end, a scalable and cost‐effective strategy is presented for synthesizing a series of 3d transition metal–bismuth (TM–Bi) atomic pairs anchored on carbon nitride (CN) and investigate their potential to activate SRRs in lithium‐sulfur batteries (LSBs). An initial screening identifies Ni–Bi/CN and Co–Bi/CN as highly effective in improving rate performance. Detailed analysis shows these catalysts promote direct electrochemical transitions and rapid Li_2_S nucleation over competing chemical reactions, enabling high charge–discharge rates while preventing active material loss and enhancing stability. Electrochemical analysis, density functional theory, and operando spectroscopy reveal that TM‐Bi pairing shifts d‐band states closer to the Fermi level and modulates HOMO–LUMO levels, promoting lithium polysulfide (LiPS) interaction and facilitating efficient charge transfer. These findings offer valuable insights for designing advanced catalysts for LSBs and broader electrocatalytic applications.

## Graphical abstract

A scalable strategy for synthesizing transition metal–bismuth atomic pairs on carbon nitride to accelerate sulfur redox reactions in lithium–sulfur batteries is presented. Nickel‐bismuth and cobatl‐bismuth catalysts improve rate performance by promoting direct electrochemical transitions and rapid Li_2_S nucleation, minimizing sulfur loss, and enhancing stability.

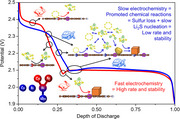

## 1 Introduction

Conversion‐type cathodes, including those based on oxygen, chalcogens, and halogens, hold great promise for next‐generation high‐energy‐density rechargeable batteries. However, despite significant progress in recent years, their practical implementation still faces critical challenges.^[^ ^1^ ^]^ Among these, the activation of the conversion reaction stands out as a major hurdle, requiring catalysts that are both highly active and stable while contributing minimal additional weight to the system.^[^ ^2^, ^3^ ^]^ Sulfur cathodes represent a paradigmatic example of high‐energy‐density, conversion‐type cathodes, offering an impressive theoretical energy density of 2600 Wh kg^−1^ while relying on a low‐cost, abundant, and environmentally benign resource.^[^ ^4^ ^]^ However, the sluggish sulfur redox kinetics, which lead to low charge/discharge rates and limited cycling stability, remain a major challenge to their commercial viability. Addressing this limitation necessitates the development of catalysts that efficiently accelerate the sulfur redox reactions (SRRs), prevent the loss of active material,^[^ ^5^ ^]^ and thereby enhance overall battery performance.^[^ ^6^, ^7^, ^8^ ^]^

The development of optimized catalysts for the SRRs remains a significant challenge due to their intrinsic complexity, involving multiple electrochemical steps alongside coupled chemical processes such as dissociation, disproportionation, and comproportionation.^[^ ^9^, ^10^, ^11^, ^12^, ^13^, ^14^, ^15^ ^]^ Additionally, the reaction pathway is likely influenced by various factors, including charge/discharge rate, cycling history, and cell architecture, particularly solvent composition and cathode additives. While numerous mechanisms have been proposed to describe this conversion process, the specific sulfur species involved and the liquid–solid transition mechanisms remain a topic of ongoing debate. A critical gap in current understanding lies in the formation and transformation of solid phases and the role of catalysts in facilitating these processes. For instance, C. Prehal et al. reported evidence of Li_2_S formation from Li_2_S_2_ in a catalyst‐free solid‐state conversion process, showing that at relatively low current densities, preformed Li_2_S_2_ particles transform into aggregates of Li_2_S grains with small crystallite sizes.^[^ ^16^ ^]^ Z. Wu et al. demonstrated that a catalyst can promote epitaxial growth of Li_2_S from lithium polysulfide (LiPS) dissociation into Li_2_S_2_ and short‐chain LiPS on the Li_2_S (100) plane, followed by lithiation of Li_2_S_2_ into crystalline Li_2_S.^[^ ^17^ ^]^ S. Zhou et al., using in situ transition electron microscopy (TEM) measurements, revealed that catalysts can act as a LiPS attraction pole, drawing a dense liquid phase of LiPS that instantaneously deposits as crystalline Li_2_S particles.^[^ ^6^ ^]^ These findings highlight the complexity of the Li_2_S formation pathway and the pivotal role of catalysts in this process, emphasizing the need for a deeper understanding of catalytic mechanisms to advance technologies based on sulfur cathodes and optimize catalyst performance.

Single‐atom catalysts (SACs) have emerged as promising candidates for accelerating cathodic conversion reactions, particularly SRRs.^[^ ^18^, ^19^ ^]^ SACs offer maximized atomic utilization efficiency, unique electronic structures, and often superior catalytic activity, all achieved with minimal metal usage and added weight. Among them, 3d transition metal (TM) SACs, such as those based on manganese, cobalt, and nickel, have drawn particular attention due to their abundance, low cost, relatively low weight, and remarkable ability to catalytically enhance various electrochemical reactions, including the SRRs.^[^ ^20^, ^21^, ^22^ ^]^

Unlike 3d TM, p‐block metals have fully occupied 3d orbitals, leaving them with relatively inactive d electrons for bonding or electronic interactions.^[^ ^23^ ^]^ However, their unfilled p orbitals can interact with the 2p electrons of elements such as oxygen, chalcogens, or halogens, enabling the formation of π‐electron conjugation.^[^ ^24^ ^]^ This interaction transforms the localized p orbitals into a delocalized state, effectively mimicking the electronic characteristics commonly associated with the d orbitals of TMs. As a result, p‐block elements can also create tunable and multifunctional electronic structures, contributing to catalytically accelerating conversion reactions.^[^ ^25^ ^]^ These unique properties make p‐block elements increasingly attractive metal electrocatalysts, offering a cost‐effective and versatile alternative to traditional noble metal catalysts.^[^ ^26^ ^]^

Among the p‐block metals, bismuth stands out for its relatively high abundance, stability, low cost, and moderate environmental and health impact. Despite these advantages, its potential in catalysis remains largely unexplored. With an electronic configuration of [Xe] 4f^14^5d^10^6s^2^6p^3^, Bi benefits from the weak shielding effect of 4f electrons, a phenomenon commonly referred to as lanthanide contraction.^[^ ^24^ ^]^ This effect imparts Bi(III) with soft Lewis acidity, making it particularly effective in activating polysulfides. In sulfur cathodes, the 6p orbitals of Bi readily hybridize with the 2p orbitals of sulfur, enhancing charge transfer and lowering the energy barrier for polysulfide conversion.^[^ ^26^ ^]^ While these interactions enhance Bi's ability to bind polysulfides and catalyze the SRRs, they also introduce a limitation. The strong interaction between Bi and S─S multiple bonds hampers the cleavage of S─Bi bonds after the sulfur reduction process, thereby reducing the efficiency of subsequent reaction cycles.^[^ ^9^ ^]^

To address these issues, we propose harnessing the robust π‐activation properties of 6p main‐group elements in synergy with the versatile and multifunctional nature of 3d TMs to activate the SRRs. To maximize catalytic activity with minimal weight addition, we suggest integrating these components into asymmetric diatomic catalysts (DACs).^[^ ^27^ ^]^ DACs are particularly advantageous for complex reactions requiring intricate electronic interactions, as their dual metal sites provide unique electronic structures and coordination environments that often surpass the performance of SACs. DACs also offer greater tunability compared to SACs, thanks to their inherent asymmetry, broader compositional flexibility, and the potential synergistic effects between the two distinct atomic centers. These features enhance catalytic activity, selectivity, and stability across various reactions.^[^ ^28^ ^]^ In DACs, the local charge symmetry is disrupted, leading to a dynamic redistribution of the electron cloud density at the active center during the reaction.^[^ ^18^, ^29^ ^]^ This redistribution significantly influences the catalytic properties but adds complexity to the system, which has been seldom investigated. Understanding this interaction between the paired elements is crucial for optimizing DAC performance and unlocking their full potential in catalytic applications.

This study introduces 3d‐6p DACs to accelerate SRR kinetics in lithium–sulfur batteries (LSBs) and investigates the S_8_ ⇄ Li_2_S conversion pathway behind their enhanced rate performance and stability. We first present a general, straightforward, low‐cost, and scalable liquid‐phase method for synthesizing DACs, in which TM‐Bi atomic pairs are uniformly dispersed in a carbon nitride (CN) matrix. Three TMs are considered: Co and Ni with partially filled 3d orbitals, and Mn with a half‐filled 3d shell. LSBs incorporating these catalysts as cathode additives are then systematically evaluated and compared with Co/CN and Bi/CN reference electrocatalysts. Experimental results identify Ni–Bi/CN and particularly Co–Bi/CN as the most effective TM‐Bi/CN catalysts for improving cathode performance. Detailed analysis of the charge and discharge processes reveals that these best‐performing catalysts significantly enhance the electrochemical S_8_ → Li_2_S_8_ conversion at relatively high voltage, effectively suppressing the simultaneous formation of Li_2_S_4_ and the chemical comproportionation reaction to Li_2_S_2_. Additionally, cathodes incorporating these catalysts exhibit faster activation of the Li_2_S phase, enabling the maintenance of high capacities at elevated charge/discharge rates. To further elucidate the catalytic mechanisms, first‐principles calculations are employed to correlate the electronic interactions within the coupled 3d‐6p atomic pairs with their electrocatalytic performance. Overall, this work provides valuable insights for the rational design of practical Li–S catalysts, particularly DACs, unlocking their potential to advance LSB technology, optimize other conversion‐type cathodes, and expand applications in various electrocatalytic processes.

## 2 Results and Discussion

### 2.1 DAC Synthesis and Physicochemical Characterization

**Figure 1:**
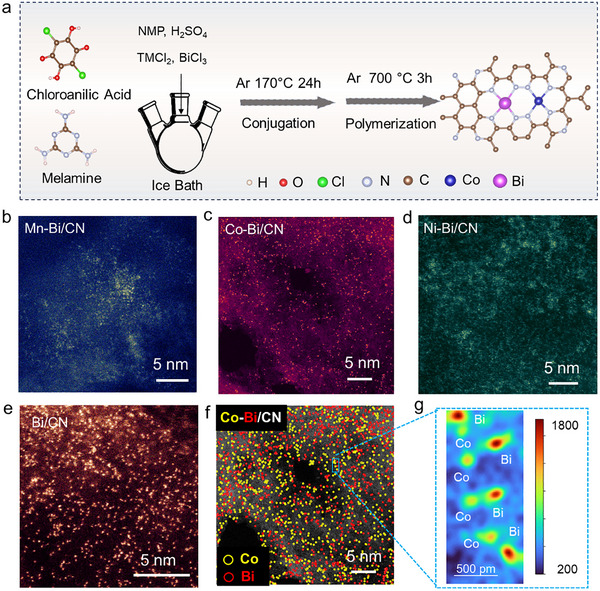
Synthesis and physicochemical characterization of TM‐Bi/CN DACs. a) Scheme of the synthesis mechanism. b–e) Atomic‐resolution AC HAADF STEM images (false‐colors) of TM‐Bi/CN: Mn‐Bi/CN (b) Co‐Bi/CN (c), Ni‐Bi/CN (d), and Bi/CN (e). f) Co‐Bi/CN HAADF‐STEM image identifying Co (yellow) and Bi (red) atoms. g) 3D intensity plot of a magnified region of the Co‐Bi/CN HAADF‐STEM image.

A series of CN‐supported TM‐Bi atomic pairs, including Mn–Bi/CN, Co–Bi/CN, and Ni–Bi/CN was produced through the pyrolysis of a mixture of chloroanilic acid and melamine containing small amounts of the corresponding metal chlorides (**Figure** **1**a, see details in the Experimental Section, Supporting Information). Additionally, reference Bi/CN, Co/CN, and CN materials were synthesized using the same procedure and evaluated as control samples. Chloroanilic acid exhibits a distinctive molecular structure, featuring a six‐membered carbon ring decorated with highly electronegative groups, including ─Cl, ═O, and ─OH, which contribute to its unique chemical reactivity and versatility. When combined with melamine, these electronegative groups interact with hydrogen (─H) from the Lewis base group (─NH_2_) on melamine. This interaction promotes the detachment of small molecules, which, upon pyrolysis, results in the formation of a nitrogen‐enriched carbon framework with numerous nitrogen‐terminated structural voids. These voids provide abundant sites for metal–N bonding, enabling the incorporation of a high loading of Lewis acid atoms, such as Bi and 3d TMs, with excellent dispersion. This high density of trapping sites is crucial for synthesizing atomically dispersed catalysts, effectively suppressing the formation of metal‐based nanoparticles.^[^ ^30^ ^]^

Scanning electron microscopy (SEM) images show that all samples feature irregular nanosheet‐like structures (Figure S1, Supporting Information). Aberration‐corrected high‐angle annular dark field scanning TEM (AC HAADF STEM) imaging, combined with energy dispersive X‐ray spectroscopy (EDS) analysis, reveals that the TM‐Bi DACs and the reference samples consist of a CN matrix with a loose, flocculent morphology and a uniform distribution of TMs (Mn, Co, and Ni), Bi, C, and N (Figures S2–S5, Supporting Information). X‐ray photoelectron spectroscopy (XPS) further corroborates the presence of these elements (Figure S6, Supporting Information). It is noteworthy that the relative signal intensities of TM and Bi significantly differ, which is attributed to their notably distinct photoionization cross‐sections. Specifically, heavier elements such as Bi have larger cross‐sections than lighter TMs, resulting in stronger XPS signals at comparable concentrations. Additionally, the XPS spectrum in Figure S7 (Supporting Information) confirms the absence of any metal elements in the CN sample. Atomic‐resolution AC HAADF STEM images (Figure 1b–e) display a high density of metal atoms distributed as single atoms across the CN matrix, validating the synthesis process's ability to trap different elements in single‐atom form.

Using the Co–Bi/CN sample as a representative case, atomic‐resolution AC HAADF STEM images were analyzed with a Laplacian of Gaussian algorithm to identify regions of rapid intensity variation, thereby detecting metal atoms. The intensity values of the identified metal atoms exhibit a bimodal distribution, indicating the presence of two distinct intensity levels corresponding to two different atomic species (Figure S8, Supporting Information). Given that atomic‐resolution AC HAADF STEM intensity scales approximately with the atomic number (Z),^[^ ^31^ ^]^ the position of the two peaks aligns well with the atomic numbers of Co and Bi when taking into account an underlying 3 nm‐thick C/CN layer contributed by the CN matrix and the ultrathin carbon grid used for STEM characterization. Furthermore, the ratio of the integrated peak areas matches the atomic ratio of Co and Bi detected by EDS analysis (Table S1, Supporting Information), validating the algorithm's ability to differentiate between the two metal atoms (Figure 1f). Building on this differentiation, we conducted a detailed analysis of the spatial organization of Co and Bi atoms within the CN matrix (Figure S9, Supporting Information). Statistical results indicate that ≈32% of the metal atoms form coupled Co–Bi pairs, as those shown in Figure 1g, another 31% were organized as either Bi–Bi or Co–Co pairs, and the remaining 37% existed as isolated Co or Bi atoms. Inductively coupled plasma mass spectrometry showed the atomic metal concentration to be ≈1% with a TM/Bi ratio of ≈1 (Table S2, Supporting Information).

**Figure 2:**
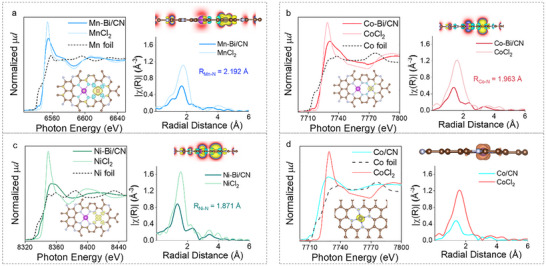
Coordination environment and structure of TM‐Bi/CN DACs. a–d) TM K‐edge XANES spectra (left) and FT of the EXAF spectra (right) of Mn‐Bi/CN (a), Co‐Bi/CN (b), Ni‐Bi/CN (c), and Co/CN (d). The spectra of the corresponding metal foils and metal(II) chlorides are also included as references. Structural parameters extracted from the first shell Fourier analysis are shown in Table S3 (Supporting Information). Insets show the ball and stick structure models, where brown = C; grey = N; pink = Bi; and other colors = TMs. The models include electronic density data obtained from DFT calculations, where cyan = spin down electron cloud, and yellow = spin up electron cloud. The isosurface level is 0.001 e a_0_^−3^.

X‐ray absorption spectroscopy (XAS) was used to identify the local geometry of the TMs. Extended X‐ray absorption fine structure (EXAFS) analysis of the TM *K*‐edges allowed us to extract interatomic distances of the first coordination shell for TM‐Bi systems and construct a series of density functional theory (DFT) atomic structure models for TM‐Bi/CN complexes. X‐ray absorption near‐edge structure (XANES) spectra and the generated molecular structure models are shown in **Figure** **2**. The peak ≈2 Å in the Fourier‐transformed (FT) EXAFS spectrum corresponds to the TM─N bond. The length of this bond is influenced by the TM chemical environment, particularly the presence of Bi, which should be evident in the experimental data, offering a means to verify the formation of coupled TM–Bi pairs. First shell Fourier analysis revealed the interatomic TM‐N distances to be *R*_Mn−N_ = 2.192 Å, *R*_Co−N_ = 1.963 Å, and *R*_Ni−N_ = 1.871 Å (Table S2, Supporting Information). The simulated bond lengths from the generated asymmetric pair model (TM‐Bi/CN) and experimental results are displayed in Figure S10 (Supporting Information). The experimental TM─N bond lengths align with those simulated for the TM─Bi/CN model, confirming the presence of a substantial amount of asymmetric coupled metal pairs, consistent with atomic resolution AC HAADF STEM analysis.

Additional extensive TEM and STEM screening revealed no evidence of high‐density contrast features attributable to metal clusters or nanoparticles. The absence of such signatures across multiple regions of analysis indicates that the metal species are atomically dispersed rather than aggregated into larger crystalline or amorphous domains. Besides, X‐ray diffraction (XRD) and Raman spectroscopy analyses further confirmed the absence of Bi and TM‐based crystalline domains (Figures S11,S12, Supporting Information). The XRD patterns of Bi/CN, Co/CN, and Co–Bi/CN display only the broad diffraction features characteristic of graphitic carbon nitride, with no additional peaks corresponding to crystalline rhombohedral Bi or TM phases (Figure S11, Supporting Information). Raman spectra of all three samples exhibit similar characteristic D and G bands of carbon nitride (Figure S12, Supporting Information), without any new bands assignable to Bi–Bi or Co–Co vibrations. Overall, these observations imply that the incorporation of Bi and/or TM atoms does not disrupt the CN framework nor induce the formation of metal‐based domains.

### 2.2 DAC Performance in LSBs

To assess the electrochemical performance of DACs as catalytic additives in sulfur‐based cathodes, CR2032 coin‐type full cells were assembled using a 1 M lithium bis(trifluoromethanesulfonyl)imide (LiTFSI) and 0.2 m LiNO_3_ in a 1,3‐dioxolane (DOL)/dimethoxyethane (DME) electrolyte, a Li foil anode, and a TM‐Bi/CN/S cathode. The cathode was prepared by casting a mixture with an 8:1:1 mass ratio of TM‐Bi/CN/S, conductive carbon (Super P, SP), and polyvinylidene fluoride binder. TM‐Bi/CN/S was produced by heating a 1:3 mixture of TM‐Bi/CN and S at 155 °C (Figure S13, Supporting Information, see details in the Experimental Section, Supporting Information). The cells were cycled within the voltage range of 1.7–2.8 V. Cells based on Bi/CN/S, Co/CN/S, CN/S, and SP/S were also assembled and tested as a reference.

In the commonly used DOL/DME electrolyte, the galvanostatic discharge profiles of LSBs typically display two distinct plateaus separated by a gradual transition. The combined capacity of the first plateau and the transition region (*Q_1_*) is usually attributed to the four‐electron reduction of S_8_ to Li_2_S_4_. The initial plateau is generally associated with the conversion of S_8_ to Li_2_S_8_, a two‐electron process with an equilibrium potential of 2.41 V that contributes to 50% of *Q_1_*.^[^ ^12^ ^]^ The subsequent sloped transition represents the reduction of Li_2_S_8_ to Li_2_S_4_, which involves two additional electrons and occurs at a lower potential (2.24 V).^[^ ^12^ ^]^ The sloped nature of this transition arises from the multiple disproportionation and comproportionation reactions that can occur in this region, particularly considering the relatively low solubility of Li_2_S_4_ in the DOL/DME electrolyte. The second discharge plateau, *Q_2_*, overall accounts for the conversion of Li_2_S_4_ to Li_2_S, a process that is assumed to occur through the precipitation of soluble LiPS into Li_2_S_2_, followed by its crystallization into Li_2_S. This step involves the transfer of 12 electrons, accounting for 75% of the cell's total capacity, and it is governed by sluggish liquid–solid and solid‐state reactions, making it a critical bottleneck in LSB performance.

**Figure 3:**
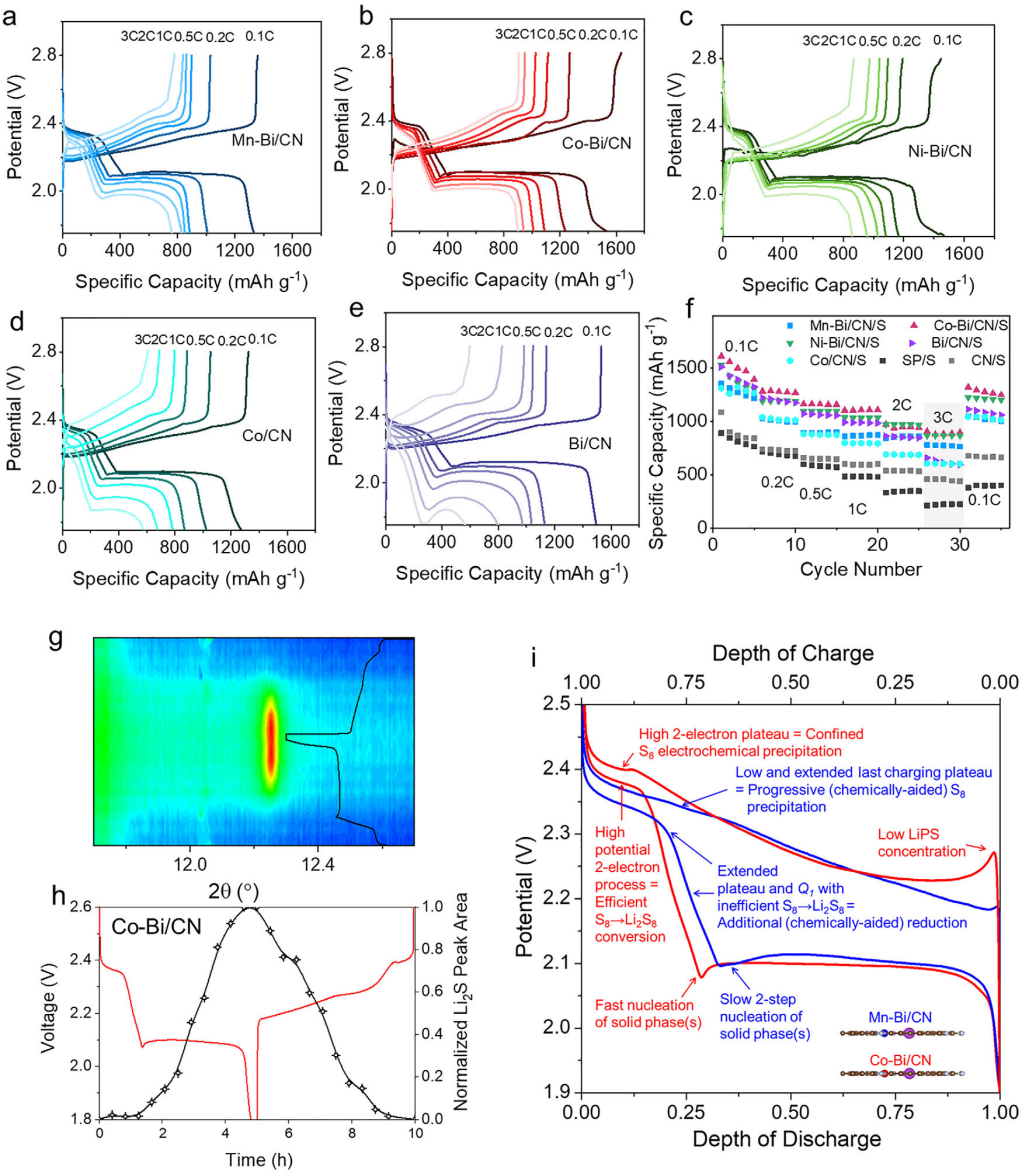
Electrochemical performance of TM‐Bi/CN/S cathodes. a–e) GCD profiles of Mn–Bi/CN/S (a), Co–Bi/CN/S (b), Ni–Bi/CN/S (c), Co/CN/S (d), and Bi/CN/S (e) at different current densities. f) Rate performance of TM‐Bi/CN/S, Co/CN/S, Bi/CN/S, CN/S and SP/S full cells. g) O*perando* XRD patterns of Co–Bi/CN/S and GCD profile. h) Normalized Li_2_S (220) XRD peak area obtained from *operando* XRD analysis and GCD profile. i) GCD profiles of Co–Bi/CN/S (red) and Mn–Bi/CN/S (blue) cells labelled with the key mechanisms determining rate performance and stability.

All the cells incorporating catalytic additives achieved high capacities approaching the theoretical limit, indicating a high sulfur utilization (**Figure** **3**–g; Figure S14, Supporting Information). In contrast, the catalyst‐free SP/S cell displayed significantly lower capacity, highlighting the critical role of catalysts in the activation of the electrochemical process (Figure S14, Supporting Information). While all catalysts promoted the cell rate performance and stability, notable differences among the tested DAC‐based electrodes were observed, further demonstrating their substantial influence on sulfur redox kinetics and overall battery performance.

As consistently observed in previous literature, all tested LSBs experience capacity loss during the initial cycles at low galvanostatic charge–discharge (GCD) rates, primarily due to the dissolution and redistribution of LiPS throughout the cell (Figure S14, Supporting Information). However, while some of the LSBs continue to suffer significant capacity degradation during high‐rate cycling, the cells based on Ni–Bi/CN/S and Co–Bi/CN/S demonstrate minimal capacity loss at high rates. These electrodes also exhibited the best rate performance (Figure 3a–h), achieving high specific capacities at the highest current rates tested. Specifically, at 3C, Co–Bi/CN/S delivered a discharge specific capacity of 906 mAh g^−1^, that is, 56% of the capacity delivered at 0.1C, significantly outperforming the Mn–Bi/CN/S electrode and most previously reported cathodes.^[^ ^3^, ^5^, ^32^, ^33^, ^34^, ^35^ ^]^ The rate performance of the Co–Bi/CN/S‐based cell also surpassed that of the Co/CN/S and Bi/CN/S reference electrodes, which retained only 46% and 42% of their capacities for Co/CN/S for Bi/CN/S, respectively, when the current density was increased from 0.1C to 3C.

A closer examination of the galvanostatic discharge profiles at the lowest tested current density (0.1C) reveals two differentiated behaviors (Figure S15, Supporting Information). On the one hand, the best‐performing cathodes, those based on Ni–Bi/CN/S and Co–Bi/CN/S, exhibit the highest first discharge plateau, at an average potential of ≈2.38 V. This plateau contributes ≈50% of the first‐step capacity (*Q_1_*), which represents roughly one‐quarter of the total discharge capacity, yielding a *Q_2_/Q_1_* ratio of ≈3. This aligns with the simplified interpretation of the sulfur redox pathway, where the first plateau corresponds to the 2‐electron S_8_ → Li_2_S_8_ transition, the sloped region accounts for the subsequent 2‐electron Li_2_S_8_ → Li_2_S_4_ conversion, and the second plateau represents the 12‐electron Li_2_S_4_ → Li_2_S transformation.

On the other hand, Mn–Bi/CN/S, Co/CN/S, and Bi/CN/S demonstrate substantially superior performance compared to SP/S, rivalling that of previously reported high‐performance catalysts (Figure S15, Supporting Information).^[^ ^3^, ^5^, ^32^, ^33^, ^34^, ^35^ ^]^ However, their performance was inferior to that of the best‐performing DACs developed in this study, particularly in terms of rate performance and stability. These relatively less‐performing electrodes display initial discharge plateaus at a lower average voltage (≈2.34 V), indicating limitations in the activation of the S_8_ → Li_2_S_8_ conversion, thereby requiring additional overpotential. Notably, the Mn–Bi/CN/S and particularly Bi/CN/S electrodes display a significantly extended first plateau that accounts for 60% of *Q_1_*, reaching approximately three electrons per S_8_ molecule. This suggests that the initial plateau involves sulfur electrochemical reduction beyond Li_2_S_8_, probably activated by the lower potentials reached during the initial discharge required by the inefficient S_8_ → Li_2_S_8_ conversion. Although the first plateau in these catalysts remains above the equilibrium potential of further Li_2_S_8_ electrochemical reduction reactions (≤2.24 V), this reaction can be facilitated by the additional driving force provided by the chemical disproportionation or comproportionation conversion of the Li_2_S_8_ reduction products, such as Li_2_S_4_. Additionally, *Q_1_* exceeds the theoretical limit of the four‐electron S_8_→2Li_2_S_4_ reaction and results in low *Q_2_/Q_1 _* ≈ 2. This implies that the first sulfur reduction step extends beyond Li_2_S_4_, involving the formation of some Li_2_S_2_. Since the equilibrium potential for the electrochemical formation of Li_2_S_2_ or Li_2_S is too low to be effective (≤2.1 V) at this discharge stage, Li_2_S_2_ needs to be formed via a chemical comproportionation reaction involving additional production of S_8_ or Li_2_S_8_ to further fuel the *Q_1_* electrochemical process and sustaining the extended first plateau, for example, Li_2_S_8 _+ Li_2_S_4 _→ S_8 _+ 2Li_2_S_2_. This reaction is likely activated in these specific electrodes, but not in the best‐performing Ni–Bi/CN/S and Co–Bi/CN/S cathodes, due to the slow S_8_ → Li_2_S_8_ conversion that enables the coexistence of a richer variety of sulfur species in the presence of S_8_, thereby facilitating comproportionation reactions that generate additional S_8_ capable of heterogeneously precipitating. Since the formation of Li_2_S_2_ through chemical comproportionation can occur away from the electrode surface, part of the active sulfur may become electrochemically isolated and be lost during each cycle, contributing to capacity fade and reduced long‐term cycling stability. This capacity loss may be masked by the LiPS migration‐induced losses at slow discharge rates, but it becomes evident at higher discharge rates (Figure S14, Supporting Information). Post‐cycling optical images of separators (Figure S16, Supporting Information) support this observation, revealing sulfur species deposition on the Bi–CN/S separator, whereas no such deposition is evident on the Co–Bi/CN/S separator.

When normalizing the discharge profile to the capacity achieved at 1.9 V (Figure S17, Supporting Information), we observe that all the less‐performing LSBs, including the SP/S cell, exhibit a relatively longer first plateau, along with a relatively low *Q_2_/Q_1_* ratio (Table S4, Supporting Information). This suggests that the commonly observed low *Q_2_/Q_1_* ratio in these systems is not solely attributable to incomplete LiPS reduction, as often assumed. Instead, it may also result from a relatively extended *Q_1_* step beyond four electrons per S_8_ participating in the reaction, likely caused by the inefficient S_8_ → Li_2_S_8_ conversion that facilitates additional electrochemical reactions followed by chemical disproportionation processes, ultimately leading to the precipitation of some Li_2_S_2_.

At the onset of the second discharge plateau, the Ni–Bi/CN/S and Co–Bi/CN/S electrodes exhibit a distinct sharp potential dip, which is attributed to the Li_2_S_2_/Li_2_S nucleation barrier and remains relatively unaffected when increasing the discharge rate (Figure S18, Supporting Information). No additional features are observed within this plateau. Operando XRD (Figure 3g,h) shows that the Li_2_S phase appears immediately after the dip, indicating that Li_2_S forms either directly from or concurrently with the Li_2_S_2_ through the reduction of solution‐phase precursors from the very start of the second plateau. Conversely, for the Mn–Bi/CN/S sample, Li_2_S formation becomes evident only sometime after the voltage reaches 2.1 V, followed by a progressive growth until the end of discharge (Figures S19,S20, Supporting Information).

In contrast, at 0.1C, the Mn–Bi/CN/S and Bi/CN/S electrodes exhibit a less pronounced yet significantly more extended potential dip at the onset of the second plateau, characterized by two distinct steps (Figure S18, Supporting Information). Co/CN/S electrodes exhibit an even more pronounced two‐step potential dip. This dip and the overall plateau overpotential strongly increase with the discharge rate for all these electrodes. The reduced depth of the potential dip at 0.1C obtained for these catalysts is consistent with the formation of Li_2_S_2_ precipitates in the previous step (*Q_1_*), which subsequently facilitates the electrochemical deposition of short‐chain polysulfides on the pre‐nucleated Li_2_S_2_ at the onset of *Q_2_*. However, this shadow dip comes at the cost of an extended prolongation that suggests a delayed Li_2_S nucleation. This delayed onset of the Li_2_S nucleation occurs either because the activated Li_2_S_2_ growth prevents reaching the lower potentials required for Li_2_S nucleation at the onset of *Q_2_* or because the catalyst surface is covered by a solid Li_2_S_2_ layer that requires the less favorable solid‐to‐solid phase nucleation over the more accessible liquid‐to‐solid pathway. In the best cases, the dip extends for ≈1000 s, which constitutes only a small fraction of the total discharge time at 0.1C (36000 s). Consequently, a nearly complete conversion of S_8_ to Li_2_S remains attainable at these low rates. However, at higher rates, such as 1C (3600 s full discharge) and above, this extended nucleation time becomes a critical bottleneck, significantly restricting Li_2_S crystallization and thereby strongly limiting the capacities obtained at relatively high rates. Post‐mortem SEM analysis of the discharged cathodes confirmed that the reference Co/CN and Bi/CN catalysts failed to promote Li_2_S nucleation even at moderate current densities, resulting in irregular Li_2_S_2_ deposits rather than well‐defined Li_2_S crystals (Figure S21, Supporting Information). In contrast, the Co–Bi/CN/S cathode displayed clear crystalline features after discharging at 0.5C and 2C current densities (Figure S21, Supporting Information)

Upon normalizing the discharge profiles to the maximum capacity of each step (*Q_1_* and *Q_2_*, Figure S22, Supporting Information) and using the 0.1C discharge profile as a reference, we observe that the Co–Bi/CN/S electrode exhibits minimal overpotential increase with the current rate during the first plateau, corresponding to the formation of Li_2_S_8_. This indicates fast reaction kinetics and an absence of diffusion limitations, as the reactant is initially in the solid state, and Li_2_S_8_ dissolution likely occurs rapidly at the beginning of discharge when no LiPS is yet present in solution (Figure S22, Supporting Information). A noticeable overpotential increase appears midway through the first step, coinciding with the transition between the first and second plateaus. This is attributed to diffusion limitations, as a significant fraction of the generated Li_2_S_8_ fueling this second reaction is now dissolved in solution. However, the overpotential stabilizes during this transition and remains steady throughout the second plateau, where Li_2_S_2_ and Li_2_S form and crystallize from solution‐phase precursors, encountering the same diffusion constraints as in the Li_2_S_8_ → Li_2_S_4_ conversion. The Co/CN/S electrode exhibits a similar overpotential trend with increasing cycling rates, showing a moderate initial rise that becomes more pronounced at the transition between the two plateaus before stabilizing in the second plateau. However, the overpotential increase in Co/CN/S is higher than that in Co–Bi/CN/S, suggesting lower catalytic activity or a reduced ability to interact with LiPS in solution due to a lower LiPS adsorption energy, as discussed later. In contrast, the Bi/CN/S electrode exhibits a lower initial increase in overpotential compared to the Co/CN/S electrode during the first step, attributed to enhanced interactions with LiPS. However, as sulfur reduction progresses, the overpotential gradually increases throughout the first plateau and continues to rise into the second plateau. This behavior suggests increasing difficulties in breaking additional S─S bonds as the LiPS chain shortens, likely due to the strong interaction between Bi and S within LiPS, as shown below.

Focusing on the galvanostatic charge curves, distinct charging profiles for the two types of catalysts discussed above are observed (Figures S23,S24, Supporting Information). The Ni–Bi/CN/S and Co–Bi/CN/S electrodes show a pronounced initial overpotential peak, followed by a steady rise in charging potential, eventually reaching a plateau at ≈2.40 V. This plateau, extending for two electrons per S_8_ molecule, corresponds to the final Li_2_S_8_ → S_8_ step. Interestingly, as the charge rate increases, the initial overpotential peak decreases, suggesting different dominant mechanisms at low and high cycling rates. This initial overpotential is associated with an additional discharge plateau observed at ≈1.8 V (Figure S14, Supporting Information), which can accommodate up to two electrons per S_8_ molecule. Notably, this additional plateau is pronounced only in high‐performing electrodes and exclusively at low discharge rates (<1C). The disappearance of this discharge plateau correlates with the vanishing of the overpotential peak at the onset of charging.

Discharge plateaus within this voltage range have been attributed to reactions involving LiNO_3_ electrolyte additives.^[^ ^36^ ^]^ However, in the present study, the selective appearance of this plateau for certain catalysts, its confinement to low discharge rates, indicative of a diffusion‐limited process, and its persistence even after returning to 0.2C, imply a different underlying mechanism. A plausible explanation is the direct reduction of residual, highly soluble Li_2_S_6_ into Li_2_S, which would occur at a lower potential than the 2.1 V of the second plateau.^[^ ^12^ ^]^ This extended reduction process not only increases the discharge capacity but also depletes the electrolyte of LiPS, thereby preventing undesirable reactions with the anode and enhancing the cell's stability. However, this mechanism also introduces a trade‐off. The extensive consumption of LiPS during discharge impedes the dissolution of Li_2_S at the onset of the subsequent charge process, leading to the observed initial overpotential peak.

In contrast, all the other electrodes exhibit only a minor initial overpotential at 0.1C, which rapidly increases with the charge rate. This behavior suggests the sufficient presence of LiPS at the discharge onset but a limited ability to interact with them effectively. These electrodes also show a steeper voltage rise, with a lower potential (≈2.36 V) more extended final plateau, corresponding to the transfer of approximately four electrons per S_8_. Similar to the discharge process, these electrodes facilitate the simultaneous and premature formation of Li_2_S_8_ and S_8_ from Li_2_S_4_ or other short‐chained LiPS formed during the electrochemical dissolution of Li_2_S_2_/Li_2_S. The coexistence of these species promotes chemical comproportionation reactions, resulting in both homogeneous and heterogeneous sulfur nucleation within the solution and at the electrode and separator surfaces. This uncontrolled chemical formation of S_8_ leads to the precipitation of some electrically isolated sulfur (Figure S16, Supporting Information), rendering it inactive for subsequent cycles. While this chemical S_8_ precipitation can contribute to capacity loss, especially at high rates, it also facilitates S_8_ growth at lower charge potentials. In contrast, the best‐performing catalysts effectively confine the electrochemical growth of S_8_ to the electrode surface, preventing uncontrolled sulfur precipitation and enhancing the cycling stability of the battery.

As an overview, Figure 3i compares the normalized GCD curves of the Co–Bi/CN/S and Mn–Bi/CN/S cells at 0.1C, highlighting the key features that influence their performance, including rate capability and cycling stability.

**Figure 4:**
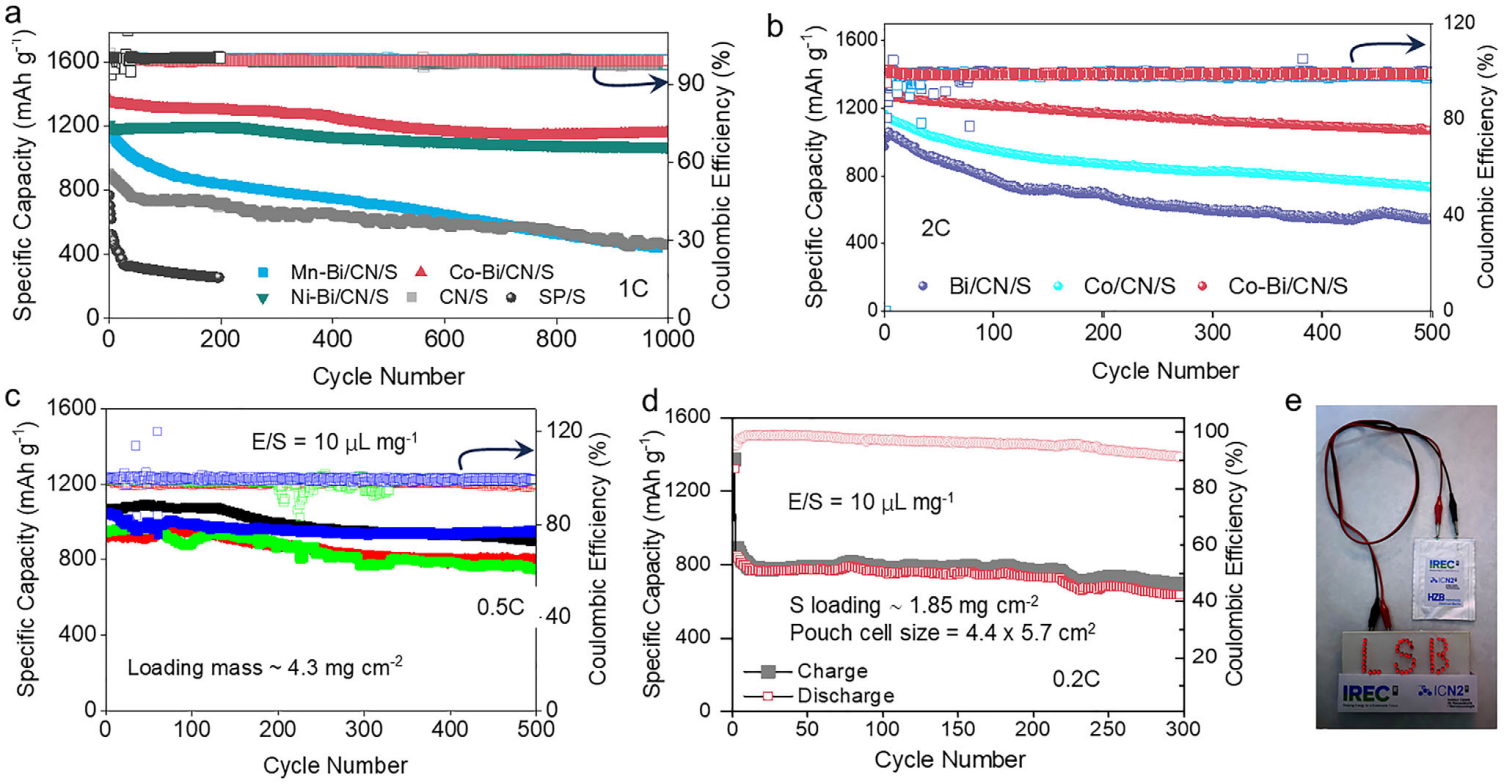
Cycling stability of coin/pouch cells at moderate/high sulfur loadings. a,b) Long‐term cycling performance at 1C of TM‐Bi/CN/S cells (a), and at 2C of Bi/CN/S, Co/CN/S, and Co–Bi/CN/S cells (b). c) Cycling performance at 0.5C of four coin cells equipped with Co–Bi–CN/S‐cathodes containing a sulfur loading of ≈4.3 mg cm^−2^. d) Cycling performance of a Co–Bi/CN/S‐based pouch cell with 1.85 mg cm^−2^ sulfur loading at 0.2C current density, and e) photograph of the pouch cell powering an LED sign.

**Figure** **4**a displays the long‐term cycling performance of the TM‐Bi/CN/S full cells tested at 1C.

Among the different catalysts, the Co–Bi/CN/S and Ni–Bi/CN/S cells exhibited significantly improved capacity retention over 1000 cycles, maintaining 86% (1153 mAh g^−1^) and 89% (1070 mAh g^−1^) of their initial capacities, respectively, along with a stable and high Coulombic efficiency exceeding 99.5%. In contrast, the Mn–Bi/CN/S‐based cell retained only 43% of its capacity under the same conditions. A summary of a literature survey on the capacity decay rates of LSBs employing related SACs and DACs is provided in Table S5 (Supporting Information).

Co–Bi/CN/S cells also exhibited the highest initial specific capacitance and the highest capacity retention rate over 500 cycles at 2C (1268 mAh g^−1^ and 84.5%), well above that of Bi/CN/S (973 mAh g^−1^ and 51%), and Co/CN/S (1167 mAh g^−1^ and 62.6%), as displayed in Figure 4b.

To further evaluate the catalytic activity under practical conditions, several coin cells using Co–Bi/CN/S cathodes with a high sulfur loading of 4.3 mg cm^−2^ were assembled and tested (Figure 4c). Remarkably, even after 500 cycles, the best electrodes retained 952 mAh g^−1^, and all cells demonstrated capacity retention rates above 85%. Moreover, at a sulfur loading of 4.6 mg cm^−2^ and under lean electrolyte conditions (6.5 µL mg^−1^), the cells exhibited stable cycling (Figure S25, Supporting Information), delivering 814.9 mAh g^−1^ initially and retaining 698.6 mAh g^−1^ after 300 cycles, with 85.8% capacity retention and 0.048% average degradation per cycle. Notably, the specific capacity at 0.5C under high sulfur loading is lower than the capacities observed at higher current rates (1C and 2C) with lower sulfur loadings (≈1.2 mg cm^−2^, Figure 4a,b). This is attributed to increased electrode thickness and reduced electrolyte‐to‐sulfur ratio at high loading, which limit ion and electron transport and enhance polysulfide shuttle effects, thereby reducing sulfur utilization despite the lower current rate. These findings demonstrate that our DAC catalysts maintain high catalytic activity and durability under realistic high‐loading and limited‐electrolyte conditions, highlighting their promise for practical high‐energy‐density LSBs. Finally, to demonstrate commercial potential, pouch cells incorporating Co–Bi/CN/S cathodes were assembled and evaluated. As shown in Figure 4d, the pouch cell delivers an initial discharge capacity of 1372 mAh g^−1^ at 0.05C. After the activation process, the second discharge capacity stabilizes at 890.7 mAh g^−1^. When cycled at 0.2C for 300 cycles, it maintained 698.6 mAh g^−1^, corresponding to a low capacity decay rate of ≈0.08% per cycle after activation, demonstrating excellent cycling stability. Additionally, Figure 4e presents a practical demonstration of the pouch cell powering an LSB sign containing 46 light‐emitting diodes (LEDs), highlighting its robust performance and potential for real‐world energy storage applications.

Post‐cycling XPS analysis (Figures S26,S27, Supporting Information) revealed that Co–Bi/CN and Ni–Bi/CN cathodes contained higher proportions of fully reduced sulfur species (Li_2_S) and exhibited markedly lower residual polysulfide content compared with other catalysts, confirming their sustained catalytic promotion of complete SRR. The N 1s spectra showed well‐preserved pyridinic‐N and pyrrolic‐N environments with only minor losses, indicating robust atomic coordination structures. F 1s and C 1s signals further suggested that DAC‐based electrodes formed more stable electrode–electrolyte interfaces, with reduced carbonate/SO_x_F_y_ species relative to SP/S.^[^ ^4^, ^37^, ^38^, ^39^, ^40^, ^41^ ^]^

Post‐mortem separator and lithium anode SEM analyses (Figure S28, Supporting Information) supported these findings: separators from cycled Co–Bi/CN and Ni–Bi/CN cells displayed much lighter coloration, indicating more effective polysulfide confinement, while SEM images of Li anodes showed smoother surfaces without severe dendrite growth, in contrast to control electrodes. These results demonstrate that Co–Bi/CN and Ni–Bi/CN DACs maintain catalytic efficiency, structural integrity, and effective polysulfide management over prolonged cycling—critical factors for achieving durable, high‐performance LSBs.

### 2.3 TM‐Bi Electronic Interaction

DFT calculations were performed to explore the electronic structure and charge redistribution within coupled heteroatom pairs. As discussed earlier, Co and Ni possess partially filled asymmetrical 3d orbitals close to the Fermi level, making them highly effective for orbital overlap and bonding with adsorbed species. These elements, even as isolated atoms, can exhibit indirect exchange interactions via the Ruderman–Kittel–Kasuya–Yosida mechanism within the CN framework, leading to ferromagnetic spin alignment. In contrast, Mn, with its half‐filled symmetric 3d shell, offers higher stability and has its 3d orbital center positioned further from the Fermi level. These distinctions partially account for the superior catalytic performance observed in Ni and Co‐based systems compared to Mn‐based ones. However, this explanation falls short of accounting for the significantly enhanced performance of the Co–Bi/CN system relative to Co/CN, suggesting that additional factors, likely linked to the synergistic effects between Co and Bi, contribute to the observed improvement.

**Figure 5:**
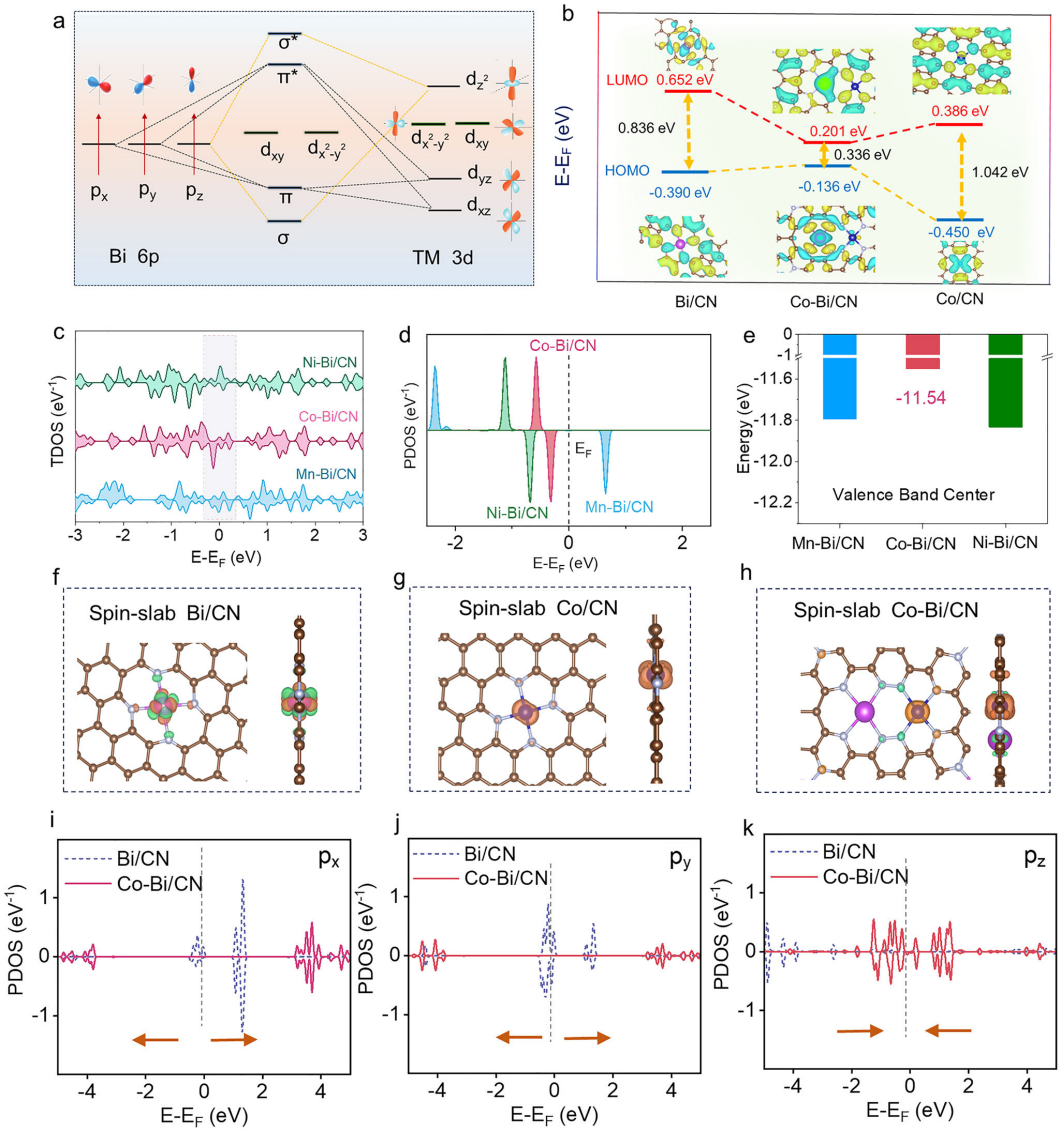
Electronic structure of TM‐Bi/CN DACs. a) Scheme of the Bi 6p – TM 3d orbital hybridization. b) HOMO and LUMO schemes for Co/CN, Bi/CN, and Co–Bi/CN. c) TM TDOS for the different TM‐Bi/CN DACs. d) TM $d_{z^{2}}$ PDOS and e) valence band center in TM‐Bi/CN. f–h) Top and cross views of the spin slabs of Bi/CN (f), Co/CN (g), and Co–Bi/CN (h). i–k) Bi 6p orbitals (p_x_, p_y_, and p_z_) PDOS from Bi/CN (dash line) and Co–Bi/CN (solid line) models.

Upon combination, the 3d orbitals of the TM (Mn, Co, and Ni) and the half‐filled Bi 6p^3^ orbitals^[^ ^29^ ^]^ may undergo hybridization to *σ*, *π*, σ*, and π* orbitals, as visualized in the scheme in **Figure** **5**a and the top and side views of the electron cloud diagram of the generated models in Figure 2. The $d_{z^{2}}$ of TM 3d can selectively hybridize with the pz orbital of Bi 6p, forming *σ* and σ* orbitals due to their compatible orbital symmetries. Meanwhile, the d_xz_/d_yz_ orbitals of TM 3d interact directionally with the p_x/y_ orbitals of Bi 6p, resulting in the generation of *π* and π* orbitals. On the other hand, the d_xy_ and d_x_^2^ − _y_^2^ orbitals of TM 3d are considered nonbonding, as their orbital orientations do not align effectively with the Bi 6p orbitals.^[^ ^42^, ^43^ ^]^ d‐p hybridization significantly reduces the energy gap between the highest occupied molecular orbital (HOMO) and the lowest unoccupied molecular orbital (LUMO). As an example, the energy gap decreases from 0.84 eV for Bi/CN and 1.04 eV for Co/CN single atom models to just 0.34 eV for Co‐Bi/CN (Figure 5b). The higher HOMO level facilitates electron transfer from the catalyst to sulfur species during the reduction process, while a lower LUMO level promotes electron acceptance from sulfur species during the oxidation process. This effect aligns with the significantly enhanced performance observed for the DACs, particularly for Co–Bi/CN, compared to the single‐metal systems, Co/CN and Bi/CN.

According to Marcus' theory^[^ ^44^ ^]^ the electron transfer rate is primarily governed by the electronic coupling between donor and acceptor states. The diverse electron configurations of 3d TMs result in distinct hybridization with Bi atoms, leading to differences in their electron capture and donation capabilities. The TM total density of states (TDOS) and the $d_{z^{2}}$ contribution to the hybridized orbitals in the Mn–Bi/CN, Co–Bi/CN, and Ni–Bi/CN, systems is displayed in Figure 5c,d. Among these heteroatom pairs, the Co 3d orbitals exhibit a $d_{z^{2}}$ partial density of states (PDOS) (Figure 5d) positioned closest to the Fermi level, followed by the Ni‐based system. In addition, Co–Bi/CN has the lowest valence band center for the entire system.(Figure 5e). This favorable electronic alignment partially explains why Ni–Bi/CN and particularly Co‐Bi/CN possess the most well‐suited electronic structure for enhanced catalytic performance.

DFT analyses also reveal significant differences in the electron cloud distribution around the metal atoms in Co–Bi/CN compared to the Bi/CN and Co/CN single‐atom models (Figure 5f–h). The PDOS of the different Co 3d orbitals within Co–Bi/CN and Co/CN shows the filled $d_{z^{2}}$ orbital contribution to shift 0.26 eV closer to the Fermi level after hybridization with Bi atoms (Figures S29,S30, Supporting Information). Concurrently, the PDOS of the Bi 6p orbitals in Co–Bi/CN shows a substantial rearrangement in the electron cloud distribution around the Bi atom (Figure 5i–k). Specifically, the p_x_ and p_y_ orbital electrons are displaced further from the Fermi level, while the p_z_ orbital electron density increases significantly near the Fermi level.

Top and side views of the DFT models of Li_2_S_4_, Li_2_S_2_, and Li_2_S molecules adsorbed on TM‐Bi/CN DACs are shown in Figures S31–S33 (Supporting Information). Both initial and final adsorption geometries are shown in Figure S34 (Supporting Information).^[^ ^45^, ^46^, ^47^ ^]^ Li_2_S_4_ primarily binds to the TM site via a TM─S bond, with Li atoms interacting with nearby nitrogen atoms but without charge transfer, thus forming fragile Li─N bonds. In contrast, Li_2_S preferentially binds to the Bi site through a Bi─S bond, with Li atoms interacting with the nitrogen surrounding the TM atom.

**Figure 6:**
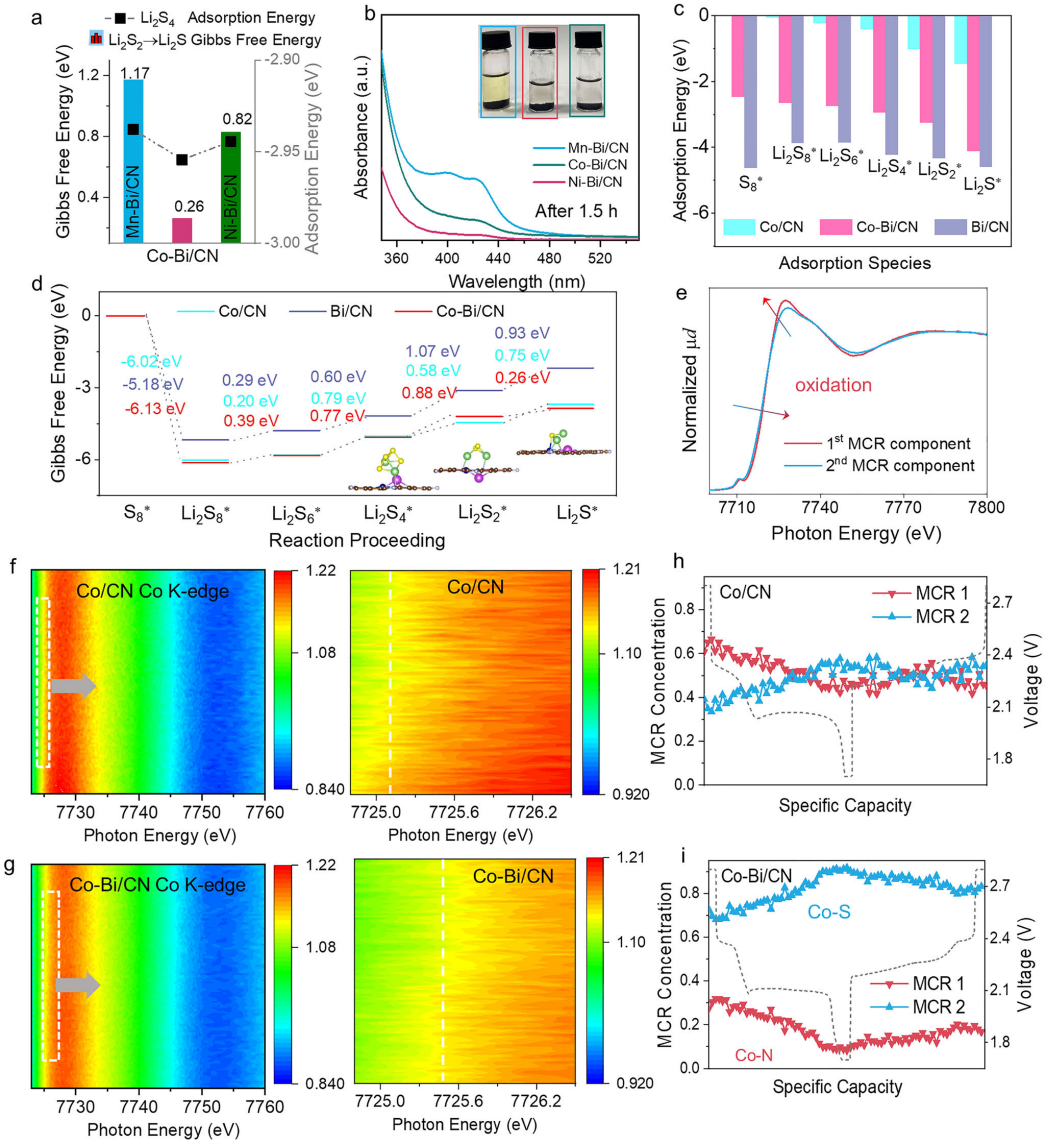
Dynamic evolution of polysulfide conversion in DACs. a) Adsorption energy of Li_2_S_4_ and Gibbs free energy change for the conversion of Li_2_S_2_ to Li_2_S on the surface of Mn–Bi/CN, Co‐Bi/CN, and Ni–Bi/CN catalysts. b) UV–vis absorption spectra of the supernatant of the vials containing the different catalysts and a Li_2_S_4_ solution after 1.5 h adsorption. The inset shows an optical photograph of the vials after 1.5 h adsorption. c) Adsorption energies of S_8_, Li_2_S_8_, Li_2_S_6_, Li_2_S_4_, Li_2_S_2_, and Li_2_S on Bi/CN, Co/CN, and Co‐Bi/CN catalysts. d) Gibbs free energy of S_8_, Li_2_S_8_, Li_2_S_6_, Li_2_S_4_, Li_2_S_2_, and Li_2_S on Bi/CN, Co/CN, and Co‐Bi/CN catalysts. e) MCR‐retrieved XANES spectra for the operando XAS Co K‐edge dataset. f–i) *Operando* XAS 3D map of Co K‐edge results for Co/CN/S and Co–Bi/CN/S, the second rows show enlarged details of the spectra to more clearly show the different oxidation states. *Operando* concentration profiles of the 2 identified Co‐species of f,g) Co/CN /S and h,i) Co–Bi/CN/S.

The adsorption energies of Li_2_S_4_ on various TM‐Bi/CN catalysts are presented in **Figure** **6**a, indicating that Mn–Bi/CN exhibits the weakest adsorption strength, while Co–Bi/CN shows the strongest. These results are consistent with the experimental LiPS adsorption findings shown in Figure 6b. The LiPS adsorption capability of the DACs was experimentally assessed by dispersing equal amounts of each TM‐Bi/CN catalyst in vials containing a Li_2_S_4_ solution. After 1.5 h, the ultraviolet‐visible (UV–vis) spectra revealed a substantial reduction in the characteristic UV absorption peak of Li_2_S_4_ for the vials containing Co–Bi/CN‐ and Ni–Bi/CN, indicating their relatively high LiPS adsorption capacities (Figure 6b). In contrast, Mn–Bi/CN exhibited significantly lower adsorption abilities. These observations were visually supported by optical photographs of the vials after Li_2_S_4_ adsorption (inset Figure 6b). The strong affinity of Ni–Bi/CN/S and Co–Bi/CN/S for Li_2_S_4_ can promote effective solid phase growth from solution, being Li_2_S_4_ a key species bridging the solid–liquid–solid reaction.^[^ ^48^ ^]^ Additionally, we performed Bader charge analysis showing minimal charge transfer on Li atoms but significant charge redistribution on S atoms upon adsorption, confirming strong TM–S interactions (Figure S35, Table S6, Supporting Information). This indicates weak electronic coupling between Li and N, disproving the presence of a substantial Li─N bond.

Side and top views of the DFT‐optimized geometrical configurations for diatomic Co–Bi/CN and single‐atom Bi/CN and Co/CN models, with adsorbed S_8_, Li_2_S_8_, Li_2_S_6_, Li_2_S_4_, Li_2_S_2_, and Li_2_S species are presented in Figures S36–S38 (Supporting Information). The corresponding adsorption energies are compiled in Figure 6c. The spin‐slab side view in Figure S39 (Supporting Information) illustrates the electron cloud distributions for the three systems. Among these, Bi/CN exhibits strong adsorption capacities for all sulfur species, Co/CN demonstrates significantly weaker adsorption, and Co–Bi/CN displays intermediate adsorption energies. The notable affinity of the Co–Bi/CN DAC for polysulfides aligns with the observed low increase in overpotential at higher discharge rates. In contrast, Co/CN exhibits relatively weak interactions with LiPS, which is consistent with the notable rise in overpotential as the discharge rate increases, as previously noted. Interestingly, while Bi demonstrates a strong affinity for polysulfides, it struggles to sustain the reaction pace at high discharge rates. This observation suggests the presence of additional underlying mechanisms that hinder its performance under rapid cycling conditions.

Figure 6d presents the Gibbs free energy evolution of the sulfur reduction reaction on the surfaces of Co–Bi/CN, Bi/CN, and Co/CN catalysts at the molecular level. Bi/CN shows high overpotentials for the formation of both Li_2_S_2_ and Li_2_S, while Co/CN exhibits a very low Gibbs free energy change for Li_2_S_2_ formation but a significant energy barrier for Li_2_S formation. Although these results are obtained at the molecular level and do not account for the energy of formation or crystallization of solid phases, they align with the observed extended dip at the onset of the second discharge plateau, the rapid increase in overpotential with rising rates during the second plateau, and the absence of Li_2_S formation at high current densities observed for the less performing catalysts. In contrast, Co–Bi/CN displays a notable energy barrier for Li_2_S_2_ formation, consistent with the observed dip, but a significantly lower energy barrier for the Li_2_S_2_‐to‐Li_2_S conversion. This finding is well‐supported by experimental data indicating more facile Li_2_S nucleation in this material.

Among TM‐Bi catalysts, Co–Bi/CN also demonstrates the lowest energy barrier (0.26 eV) for the Li_2_S_2_‐to‐Li_2_S conversion (Figure 6a), followed by Ni‐Bi/CN. Nucleation experiments (Figure S40, Supporting Information) further show that among DACs, Co–Bi/CN is particularly effective at promoting solid‐phase precipitation from a Li_2_S_6_ solution. Overall, these findings are consistent with the experimentally observed superior performance of Ni–Bi/CN and especially Co–Bi/CN DACs as sulfur reduction electrocatalysts.

During the reverse process of charging, compared with Co/CN and Bi/CN, Co–Bi/CN exhibits a particularly large Gibbs free energy barrier for the Li_2_S_8_ → S_8_ oxidation step. This is experimentally reflected in the relatively higher potentials required toward the end of the charging process for this final two‐electron step. Notably, this characteristic might actually be advantageous as it could help prevent chemical S_8_ precipitation outside the electrode, thereby enhancing the LSB cycling stability.

To experimentally verify the transfer of charge within the coupled metal atoms at the origin of the DACs enhanced performance and analyze its evolution with the reaction process, *operando* Co *K*‐edge XAS spectra were obtained. The *operando* Co *K*‐edge spectra of Co/CN/S and Co–Bi/CN/S cathodes revealed subtle yet analyzable spectral changes during cycling. As shown in Figure 6e–g, within the battery environment, the average oxidation state of Co in Co–Bi/CN/S was slightly lower during the entire cycle than in Co/CN/S, denoting a higher interaction with S in the former cathode. Principal component analysis was used to highlight the evolution of spectral features during the reaction. Two principal components were identified and deconvolved using the multicurve resolution (MCR) technique, implemented in the MCR‐alternating least squares (MCR‐ALS) Python library (Figure 6e).^[^ ^49^ ^]^ In both Co/CN/S and Co–Bi/CN/S, the relative concentration of the first MCR component, associated with the highest oxidation state, decreased during charging and increased during discharging, while the second MCR component exhibited the opposite trend (Figure 6h,i). These concentration profiles match the gradual formation of Co─S bonds and thus the decrease of Co─N structural bonds during the discharge process, when S_8_ is converted into progressively shorter chain polysulfides, and the reversal of this process during charging. This evolution and the lower valence state of Co in the Co─S bond compared to the Co─N bond, due to nitrogen's higher electronegativity, allowed us to assign the first MCR component to Co─N and the second MCR component to Co─S. Notably, the concentration of Co–S was significantly higher in Co–Bi/CN/S than in Co/CN/S, and the evolution of these two components was more reversible in Co–Bi/CN/S, aligning with the higher catalytic activity and improved cycling stability.

In addition to the Co–Bi heteronuclear DACs, we acknowledge that the presence of homonuclear DACs, such as Co–Co, Ni–Ni, Mn–Mn, and Bi–Bi, may also influence the catalytic performance. To address this, we conducted a comparative DFT analysis of these homo‐DACs to understand their potential impact on the adsorption of sulfur species. The analysis focused on calculating the Gibbs free energy for the adsorption of various sulfur intermediates, particularly Li_2_S_4_, on both homo‐DACs and hetero‐DACs (Figure S41, Supporting Information). Our results suggest that electron transfer primarily occurs at the sulfur atoms during adsorption, with negligible involvement of lithium atoms, indicating that no stable Li─N bonds are formed (Figure S34, Supporting Information). Notably, the Gibbs free energy barriers for the Li_2_S_4_ → Li_2_S_2_ and Li_2_S_2_ → Li_2_S transitions on Co–Bi sites are 0.58 and 0.26 eV (Figure S42, Supporting Information), respectively, demonstrating the favorable catalytic effect of the Co–Bi DACs. In contrast, homo‐DACs such as Co–Co and Mn–Mn display relatively low activation barriers for the Li_2_S_4_ → Li_2_S* transformation, with barriers of 0.60 and 0.78 eV, respectively. Bi–Bi sites show significantly higher energy barriers (1.58 eV), suggesting slower kinetics. These results highlight the superior catalytic performance of the Co–Bi heteronuclear DACs, where the asymmetric electronic environment enables better modulation of sulfur species adsorption and reaction pathways. Overall, our DFT analysis confirms that the Co–Bi DACs exhibit enhanced catalytic dynamics, further supporting the central role of heteronuclear DACs in optimizing LSB performance.

## 3 Conclusion

In conclusion, a series of TM‐Bi DACs supported on a CN substrate, namely Mn–Bi/CN, Co–Bi/CN, and Ni–Bi/CN, were successfully synthesized using a simple and low‐cost process. While all DACs demonstrated notable performances, the fastest sulfur redox kinetics were obtained in Co–Bi/CN/S and Ni–Bi/CN/S, which displayed the highest first discharge plateau (≈2.38 V) and a balanced Q_2_/Q_1_ ratio. In contrast, Mn–Bi/CN/S, Co/CN/S, and Bi/CN/S exhibited an extended first discharge plateau at a lower voltage (≈2.34 V) with a low Q_2_/Q_1_ ratio, attributed to the inefficient S_8_ → Li_2_S_8_ conversion. This inefficient conversion lowers the voltage of the first discharge plateau, thereby facilitating additional electrochemical reactions and subsequent comproportionation processes. As a result, some Li_2_S_2_ is chemically formed early in the reaction, during the Q_1_ step. The premature Li_2_S_2_ precipitation, indicated by the extended Q_1_ beyond the four‐electron S_8_‐Li_2_S_4_ process and the corresponding Q_2_ reduction, may occur outside the electrode, leading to active material loss and ultimately poor cycling stability. Additionally, the early formation of Li_2_S_2_ is linked to prolonged Li_2_S nucleation times in the less effective catalysts. This is associated with a low overpotential at the onset of the second discharge plateau, which reflects the facile heterogeneous growth of preformed Li_2_S_2_ and/or the challenging nucleation of Li_2_S through solid‐state transformation of the preformed Li_2_S_2_. The extended nucleation time observed in the less‐performing electrodes accounts for their relatively poor rate performance, as these systems struggle to initiate Li_2_S nucleation and thus fail to promote extensive Li_2_S crystallization at high rates (>1C). This stands in stark contrast to the Ni–Bi/CN/S and Co–Bi/CN/S electrodes, which sustain high capacities even at elevated discharge rates. Similarly, during charging, Ni‐Bi/CN/S and Co–Bi/CN/S exhibited a steady potential increase with a final two‐electron plateau, effectively confining the electrochemical LiPS to S_8_ conversion to the electrode. In contrast, other cathodes showed lower potential and prolonged final plateaus, which could reflect the chemical formation of electrically isolated sulfur, thereby diminishing cycling stability. DFT calculations reveal that the asymmetric coordination configuration of Co–Bi significantly modulates the local electronic structure and introduces additional electron density to Co within the Co–Bi/CN framework. This modification enhances the interaction between Co and polysulfides, facilitates Co─S bond formation, improves electron transfer, and ultimately boosts the catalytic activity of the Co sites. Consequently, this leads to Co‐Bi/CN/S‐based LSBs with superior rate performance and long‐term stability. Overall, this study not only elucidates the mechanisms of metal interaction, LiPS adsorption, and catalytic enhancement in TM‐Bi DACs but also establishes a robust platform for the rational design of optimized DACs. These advancements hold significant potential for improving conversion‐type cathodes, particularly sulfur cathodes in LSBs, as well as other electrocatalytic applications.

## Conflict of Interest

The authors declare no conflict of interest.

## Author Contributions

Conceptualization was done by J.Y. and C.Y.Z. Methodology was done by Z.L. Investigation was done by J.Y., C.H., I.P.H., C.L., C.Z., K.G., L.L., Y.L., J.L., and C.Y.Z. The original draft was written by J.Y. A.C. dealt with writing and editing. Funding Acquisition was done by A.C. and J.A. Resources was done by O.U., M.B., L.S., F.F., and J.Y.Z. Supervision was done by J.A. and A.C.

## Supporting information



Supporting Information

## Data Availability

The data that support the findings of this study are available from the corresponding author upon reasonable request.

## References

[1] S. Wang , Z. Wei , H. Hong , X. Guo , Y. Wang , Z. Chen , D. Zhang , X. Zhang , X. Yang , C. Zhi , Nat. Commun. 2025, 16, 511. 39779662 10.1038/s41467-024-55385-6 PMC11711384

[2] C. Y. Zhang , X. Lu , X. Han , J. Yu , C. Zhang , C. Huang , L. Balcells , A. G. Manjón , J. Jacas Biendicho , J. Li , J. Arbiol , G. Sun , J. Y. Zhou , A. Cabot , J. Am. Chem. Soc. 2023, 145, 18992. 37603793 10.1021/jacs.3c06288

[3] C. Li , D. Yang , J. Yu , J. Wang , C. Zhang , T. Yang , C. Huang , B. Nan , J. Li , J. Arbiol , Y. Zhou , Q. Zhang , A. Cabot , Adv. Energy Mater. 2024, 14, 2303551.

[4] X. Liang , C. Hart , Q. Pang , A. Garsuch , T. Weiss , L. F. Nazar , Nat. Commun. 2015, 6, 5682. 25562485 10.1038/ncomms6682

[5] C. Huang , J. Yu , C. Y. Zhang , Z. Cui , J. Chen , W.‐H. Lai , Y.‐J. Lei , B. Nan , X. Lu , R. He , L. Gong , J. Li , C. Li , X. Qi , Q. Xue , J. Y. Zhou , X. Qi , L. Balcells , J. Arbiol , A. Cabot , Adv. Mater. 2024, 36, 2400810. 10.1002/adma.202400810 38569213

[6] S. Zhou , J. Shi , S. Liu , G. Li , F. Pei , Y. Chen , J. Deng , Q. Zheng , J. Li , C. Zhao , I. Hwang , C.‐J. Sun , Y. Liu , Y. Deng , L. Huang , Y. Qiao , G.‐L. Xu , J.‐F. Chen , K. Amine , S.‐G. Sun , H.‐G. Liao , Nature 2023, 621, 75. 37673990 10.1038/s41586-023-06326-8

[7] C. Huang , J. Yu , Y.‐J. Lei , O. Usoltsev , L. Gong , Z. Cui , J. Li , C. Li , B. Nan , X. Lu , R. He , X. Qi , Q. Xue , J. Chai , Y. Ren , X. Bi , Y. Cheng , J. Y. Zhou , A. Skorynina , A. Bugaev , P. R. Martínez‐Alanis , L. Balcells , J. Arbiol , C. Y. Zhang , A. Cabot , Chem. Eng. J. 2025, 506, 160146.

[8] C. Huang , J. Yu , C. Li , Z. Cui , C. Zhang , C. Zhang , B. Nan , J. Li , J. Arbiol , A. Cabot , Adv. Funct. Mater. 2023, 33, 2305624.

[9] L. Zhou , D. L. Danilov , F. Qiao , J. Wang , H. Li , R.‐A. Eichel , P. H. L. Notten , Adv. Energy Mater. 2022, 12, 2202094.

[10] Y.‐C. Lu , Q. He , H. A. Gasteiger , J. Phys. Chem. C 2014, 118, 5733.

[11] S. Schaefer , H. Wang , L. Ren , S. Fu , H. Wang , ACS Appl. Mater. Interfaces 2025, 17, 10629. 39918179 10.1021/acsami.4c18988

[12] R. Liu , Z. Wei , L. Peng , L. Zhang , A. Zohar , R. Schoeppner , P. Wang , C. Wan , D. Zhu , H. Liu , Z. Wang , S. H. Tolbert , B. Dunn , Y. Huang , P. Sautet , X. Duan , Nature 2024, 626, 98. 38297176 10.1038/s41586-023-06918-4

[13] C. Barchasz , F. Molton , C. Duboc , J.‐C. Leprêtre , S. Patoux , F. Alloin , Anal. Chem. 2012, 84, 3973. 22482872 10.1021/ac2032244

[14] M. Wild , L. O'Neill , T. Zhang , R. Purkayastha , G. Minton , M. Marinescu , G. Offer , Energy Environ. Sci. 2015, 8, 3477.

[15] D. Zheng , G. Wang , D. Liu , J. Si , T. Ding , D. Qu , X. Yang , D. Qu , Adv. Mater. Technol. 2018, 3, 1700233.

[16] C. Prehal , J.‐M. von Mentlen , S. Drvarič Talian , A. Vizintin , R. Dominko , H. Amenitsch , L. Porcar , S. A. Freunberger , V. Wood , Nat. Commun. 2022, 13, 6326. 36280671 10.1038/s41467-022-33931-4 PMC9592616

[17] Z. Wu , M. Liu , W. He , T. Guo , W. Tong , E. Kan , X. Ouyang , F. Qiao , J. Wang , X. Sun , X. Wang , J. Zhu , A. Coskun , Y. Fu , Nat. Commun. 2024, 15, 9535. 39496586 10.1038/s41467-024-53797-y PMC11535435

[18] Y.‐J. Lei , X. Lu , H. Yoshikawa , D. Matsumura , Y. Fan , L. Zhao , J. Li , S. Wang , Q. Gu , H.‐K. Liu , S.‐X. Dou , S. Devaraj , T. Rojo , W.‐H. Lai , M. Armand , Y.‐X. Wang , G. Wang , Nat. Commun. 2024, 15, 3325. 38637537 10.1038/s41467-024-47628-3 PMC11026416

[19] X. Sun , Y. Qiu , B. Jiang , Z. Chen , C. Zhao , H. Zhou , L. Yang , L. Fan , Y. Zhang , N. Zhang , Nat. Commun. 2023, 14, 291. 36653348 10.1038/s41467-022-35736-x PMC9849388

[20] Z. Liang , D. Yang , P. Tang , C. Zhang , J. Jacas Biendicho , Y. Zhang , J. Llorca , X. Wang , J. Li , M. Heggen , J. David , R. E. Dunin‐Borkowski , Y. Zhou , J. R. Morante , A. Cabot , J. Arbiol , Adv. Energy Mater. 2021, 11, 2003507.

[21] K. Liu , J. Fu , Y. Lin , T. Luo , G. Ni , H. Li , Z. Lin , M. Liu , Nat. Commun. 2022, 13, 2075. 35440574 10.1038/s41467-022-29797-1 PMC9018836

[22] Q. Gong , D. Yang , H. Yang , K. Wu , J. Zhang , W. Bi , J. Diao , C. Li , J. Yu , C. Y. Zhang , M. Li , G. Henkelman , J. Arbiol , Q. Zhang , A. Cabot , ACS Nano 2024, 18, 28382. 39361502 10.1021/acsnano.4c11068

[23] J. Kang , X. Chen , R. Si , X. Gao , S. Zhang , G. Teobaldi , A. Selloni , L.‐M. Liu , L. Guo , Angew. Chem. 2023, 135, 202217428. 10.1002/anie.202217428 36775803

[24] M. Mato , J. Cornella , Angew. Chem., Int. Ed. 2024, 63, 202315046. 10.1002/anie.202315046 37988225

[25] E. M. Leitao , T. Jurca , I. Manners , Nat. Chem. 2013, 5, 817. 24056337 10.1038/nchem.1749

[26] W. Hua , T. Shang , H. Li , Y. Sun , Y. Guo , J. Xia , C. Geng , Z. Hu , L. Peng , Z. Han , C. Zhang , W. Lv , Y. Wan , Nat. Catal. 2023, 6, 174.

[27] R. Sui , B. Liu , C. Chen , X. Tan , C. He , D. Xin , B. Chen , Z. Xu , J. Li , W. Chen , Z. Zhuang , Z. Wang , C. Chen , J. Am. Chem. Soc. 2024, 146, 26442. 39267445 10.1021/jacs.4c09642

[28] Y. Wang , C. Xu , B. Li , M. Tian , M. Liu , D. Zhu , S. Dou , Q. Zhang , J. Sun , ACS Nano 2024, 18, 34858. 39716922 10.1021/acsnano.4c12536

[29] X. Cao , Y. Tian , J. Ma , W. Guo , W. Cai , J. Zhang , Adv. Mater. 2024, 36, 2309648. 10.1002/adma.202309648 38009597

[30] L. Li , Preparation of Novel Photoactive Materials: Different Pre‐Compositions, Post‐Modifications and Improved Performance, Universität Potsdam, Potsdam, Germany 2017.

[31] S. J. Pennycook , Ultramicroscopy 1989, 30, 58.

[32] J. Yu , C. Huang , O. Usoltsev , A. P. Black , K. Gupta , M. C. Spadaro , I. Pinto‐Huguet , M. Botifoll , C. Li , J. Herrero‐Martín , J. Zhou , A. Ponrouch , R. Zhao , L. Balcells , C. Y. Zhang , A. Cabot , J. Arbiol , ACS Nano 2024, 18, 19268. 38981060 10.1021/acsnano.4c05278 PMC11271176

[33] C. Y. Zhang , J. Yu , C. Huang , G. Sun , L. Balcells , J. Li , X. Qi , C. Z. Yi , J. Herrero‐Martín , L. Simonelli , F. Fauth , R. He , X. Pan , J. Li , J. Arbiol , J. Y. Zhou , A. Cabot , J. Am. Chem. Soc. 2025, 147, 7070. 39950673 10.1021/jacs.4c18236

[34] C. Huang , J. Yu , C. Y. Zhang , Z. Cui , R. He , L. Yang , B. Nan , C. Li , X. Qi , X. Qi , J. Li , J. Y. Zhou , O. Usoltsev , L. Simonelli , J. Arbiol , Y.‐J. Lei , Q. Sun , G. Wang , A. Cabot , Angew. Chem., Int. Ed. 2025, 64, 202420488. 10.1002/anie.202420488 39688080

[35] W. Xia , Y. Chen , M. Han , X. Wu , H. Yang , K. Fu , M. Chen , X. Wang , H. Shu , Adv. Funct. Mater. 2024, 34, 2400262.

[36] A. Rosenman , R. Elazari , G. Salitra , E. Markevich , D. Aurbach , A. Garsuch , J. Electrochem. Soc. 2015, 162, A470.

[37] Q. Liu , W. Jiang , J. Xu , Y. Xu , Z. Yang , D.‐J. Yoo , K. Z. Pupek , C. Wang , C. Liu , K. Xu , Z. Zhang , Nat. Commun. 2023, 14, 3678. 37344449 10.1038/s41467-023-38229-7 PMC10284918

[38] H. Chu , H. Noh , Y.‐J. Kim , S. Yuk , J.‐H. Lee , J. Lee , H. Kwack , Y. Kim , D.‐K. Yang , H.‐T. Kim , Nat. Commun. 2019, 10, 188. 30643115 10.1038/s41467-018-07975-4 PMC6331553

[39] X. Li , J. Liu , J. He , H. Wang , S. Qi , D. Wu , J. Huang , F. Li , W. Hu , J. Ma , Adv. Funct. Mater. 2021, 31, 2104395.

[40] Y. Zhao , T. Zhou , T. Ashirov , M. E. Kazzi , C. Cancellieri , L. P. H. Jeurgens , J. W. Choi , A. Coskun , Nat. Commun. 2022, 13, 2575. 35523785 10.1038/s41467-022-29199-3 PMC9076822

[41] J. D. Randall , D. J. Eyckens , F. Stojcevski , P. S. Francis , E. H. Doeven , A. J. Barlow , A. S. Barrow , C. L. Arnold , J. E. Moses , L. C. Henderson , ChemPhysChem 2018, 19, 3176. 10.1002/cphc.201800789 30253016

[42] Z. Han , S. Zhao , J. Xiao , X. Zhong , J. Sheng , W. Lv , Q. Zhang , G. Zhou , H.‐M. Cheng , Adv. Mater. 2021, 33, 2105947. 10.1002/adma.202105947 34569660

[43] Y. Zhang , C. Kang , W. Zhao , Y. Song , J. Zhu , H. Huo , Y. Ma , C. Du , P. Zuo , S. Lou , G. Yin , J. Am. Chem. Soc. 2023, 145, 1728. 36640116 10.1021/jacs.2c10345

[44] R. A. Marcus , J. Chem. Phys. 1965, 43, 679.

[45] Q. He , Z. Li , M. Wu , M. Xie , F. Bu , H. Zhang , R. Yu , L. Mai , Y. Zhao , Adv. Mater. 2023, 35, 2302418. 10.1002/adma.202302418 37279156

[46] Q. He , B. Yu , H. Wang , M. Rana , X. Liao , Y. Zhao , Nano Res. 2020, 13, 2299.

[47] K. Fei , Q. He , M. Wu , J. Liu , Z. Wei , W. Lu , Y. Zhao , J. Colloid Interface Sci. 2025, 680, 666. 39579432 10.1016/j.jcis.2024.11.091

[48] L. Wang , Z. Hu , X. Wan , W. Hua , H. Li , Q.‐H. Yang , W. Wang , Adv. Energy Mater. 2022, 12, 2200340.

[49] J. Yu , I. Pinto‐Huguet , C. Y. Zhang , Y. Zhou , Y. Xu , A. Vizintin , J.‐J. Velasco‐Vélez , X. Qi , X. Pan , G. Oney , A. Olgo , K. Märker , L. M. Da Silva , Y. Luo , Y. Lu , C. Huang , E. Härk , J. Fleming , P. Chenevier , A. Cabot , Y. Bai , M. Botifoll , A. P. Black , Q. An , T. Amietszajew , J. Arbiol , ACS Energy Lett. 2024, 9, 6178. 39698339 10.1021/acsenergylett.4c02703 PMC11650778

[50] L. Simonelli , C. Marini , W. Olszewski , M. Ávila Pérez , N. Ramanan , G. Guilera , V. Cuartero , K. Klementiev , Cogent Phys. 2016, 3, 1231987.

[51] R. Ciancio , R. E. Dunin‐Borkowski , E. Snoeck , M. Kociak , R. Holmestad , J. Verbeeck , A. I. Kirkland , G. Kothleitner , J. Arbiol , Microsc. Microanal. 2022, 28, 2900.

