# Supplementary material for: Promoting Electrochemical Reactions with Dual‐Atom Catalysts for High‐Rate Lithium–Sulfur Batteries
Source: Adv Mater. 2025 Sep 22;38(2):e11345. doi: 10.1002/adma.202511345 (PMC12783965; doi:10.1002/adma.202511345)
Supplement: Supplementary file 1 — Supporting Information [file ADMA-38-e11345-s001.pdf]

ADVANCED MATERIALS

Supporting Information

for *Adv. Mater.*, DOI 10.1002/adma.202511345

Promoting Electrochemical Reactions with Dual-Atom Catalysts for High-Rate Lithium–Sulfur Batteries

Jing Yu, Oleg Usoltsev, Irina Martynova, Chen Huang, Zhifu Liang, Ivan Pinto-Huguet, Canhuang Li, Liqiang Lu, Chaoqi Zhang, Xuan Lu, Kapil Gupta, Marc Botifoll, Laura Simonelli, François Fauth, Jin Yuan Zhou, Jordi Llorca, Yan Lu, Chao Yue Zhang, Jordi Arbiol* and Andreu Cabot**

Supporting Information

Promoting Electrochemical Reactions with Dual-Atom Catalysts for High-Rate Lithium-Sulfur Batteries

Jing Yu, Oleg Usoltsev, Irina Martynova, Chen Huang, Zhifu Liang, Ivan Pinto-Huguet, Canhuang Li, Liqiang Lu, Chaoqi Zhang, Xuan Lu, Kapil Gupta, Marc Botifoll, Laura Simonelli, François Fauth, Jin Yuan Zhou, Jordi Llorca, Yan Lu, Chao Yue Zhang*, Jordi Arbiol*, Andreu Cabot*

J. Yu, I. Martynova, C. Huang, C. Li, X. Lu, A. Cabot
Catalonia Institute for Energy Research (IREC), Sant Adrià de Besòs, Barcelona 08930, Spain
E-mail: acabot@irec.cat

J. Yu, I. Pinto-Huguet, K. Gupta, M. Botifoll, J. Arbiol
Catalan Institute of Nanoscience and Nanotechnology (ICN2), CSIC and BIST, Campus UAB, Bellaterra, 08193 Barcelona, Catalonia, Spain
E-mail: arbiol@icrea.cat

O. Usoltsev, L. Simonelli, F. Fauth
CELLS-ALBA Synchrotron, Carrer de la Llum, 2, 26, 08290 Cerdanyola del Vallès, Barcelona, Catalonia, Spain

C. Huang
Department of Chemistry, University of Barcelona, 08028 Barcelona, Catalonia, Spain

Z. Liang
College of Ocean Science and Engineering, Shanghai Maritime University, 201306 Shanghai, China

L. Lu, Y. Lu
Helmholtz-Zentrum Berlin für Materialien und Energie, Hahn-Meitner-Platz 1, 14109 Berlin, Germany

C. Zhang
College of Materials Science and Engineering, Fuzhou University, No.2, Xueyuan Road, Minhou County, Fuzhou City, Fujian Province 350108, China

J.Y. Zhou
School of Physical Science and Technology, Lanzhou University, Lanzhou, Gansu, China.

J. Llorca
Department of Chemical Engineering, Universitat Politècnica de Catalunya, EEBE, Eduard Maristany 10-14, 08019 Barcelona, Spain

C.Y. Zhang

Department of Chemistry and Biochemistry, University of California, Los Angeles, CA, USA
E-mail: chyzhang0810@g.ucla.edu

J. Arbiol, A. Cabot
ICREA Pg. Lluis Companys, Barcelona 08010, Spain

Experimental Section

Synthesis of TM-Bi/CN: Chloroanilic acid (417.96 mg, 2 mmol) was placed into a three-necked round-bottom flask under an argon atmosphere, and the flask was cooled in an ice bath. N-methyl-2-pyrrolidone (NMP) (12 mL) was used as the solvent, and melamine (504.48 mg, 4 mmol), 20 mg $BiCl_3$, and the same amount of the selected transition metal chloride ($MnCl_2$, $CoCl_2$, $NiCl_2$) were introduced into the mixture at around 0°C. While stirring vigorously, a few drops of concentrated sulfuric acid were slowly added. After 20 minutes of continuously stirring, the ice bath was removed, and the reaction mixture was allowed to warm to room temperature. The solution was then heated to 170 °C for 24 hours under an argon atmosphere. Upon cooling to room temperature, the mixture was vacuum-filtered, washed with ethanol and water three times, and freeze-dried for 24 hours. The resulting black solid was annealed at 700 °C for 3 hours under an argon atmosphere, with a temperature ramp of 5 °C/min.

Preparation of TM-Bi/CN/S: TM-Bi/CN/S was prepared through a three-step process. First, the cathode host material was mixed with sulfur powder in a 1:3 ratio. The mixture was then ground thoroughly using a mortar and subjected to heat treatment at 155°C for 12 hours. Finally, the resulting product was collected for subsequent analysis.

Synthesis of Li2S6 solution and adsorption tests: S and Li_2S were dissolved into a mixed solution of DME and DOL at a ratio of 5:1 and then heated at 80 °C overnight to obtain the Li_2S_4 solution. For the adsorption test, 15 mg of each host material was suspended in the Li_2S_6 solution and stood for 1.5 h to analyze the solution color change and its UV-vis absorption spectra.

LSB assembly: LSBs were assembled in 2032-type coin cells in an argon-filled glove box. The cathode was prepared by combining TM-Bi/CN/S, SP, and polyvinylidene fluoride (PVDF) in a weight ratio of 8:1:1, casting the mixture onto aluminum foil, with the S mass loading 1.2 mg cm^{-2}, and drying it under low vacuum at 80°C overnight. The dried cathode was then cut into circular electrodes with a diameter of 12 mm. 40 μL of electrolyte, which consists of 1.0 M lithium bis(trifluoromethanesulfonyl)imide (LiTFSI) and 0.2 M lithium nitrate ($LiNO_3$) dissolved in a 1:1 (v/v) mixture of volumetric mixture of DOL and DME. A lithium disk served as the anode.

Assembly of Single-layer Li-S Pouch Cells: Single-sided lithium metal coated on copper foil (50 μm, from China Energy Lithium Co., Ltd) was punched into pieces with 58 mm × 45 mm to serve as the Li metal anodes. Each pouch cell was assembled by stacking one single-sided sulfur

cathode (with a sulfur loading of ~1.85 mg cm^{-2}), one layer of Celgard 2400 separator, and one lithium metal anode. The stack was welded with a tab and placed into an aluminum-laminated pouch cell case. Electrolyte was injected through the top opening of the pouch cell, with the amount adjusted to achieve an electrolyte-to-sulfur (E/S) ratio of 10 μL mg^{-1}. The pouch cell was then sealed under vacuum (−25 kPa) using a hot sealer (180 °C, MSK-115-III, MTI Corp.). All cell assembly and electrolyte injection steps were carried out inside an argon-filled glove box, with oxygen and moisture levels maintained below 0.1 ppm. The assembled Li–S pouch cells were initially cycled at 0.05 C, followed by cycling at 0.2 C using an Arbin battery testing system.

Symmetric cell assembly: Symmetric cells were fabricated using a method analogous to that employed for LSBs. Two identical electrodes were prepared by combining TM-Bi/CN, SP, and PVDF in a weight ratio of 8:1:1, serving as the working and counter electrodes, respectively. The electrolyte comprised 40 μL of a solution containing 0.5 M Li_2S_6 and 1 M LiTFSI in a 1:1 (v/v) mixture of DOL and DME. The electrode loading mass was approximately 0.5 mg cm^{-2}.

Li_2S nucleation experiment: Coin cells were assembled using TM-Bi/CN as the working electrode, a lithium disk as the counter electrode, a Celgard 2400 membrane as the separator, and a 0.5 mM Li_2S_6 solution as the electrolyte. Firstly, the freshly assembled coin cell was discharged to 2.06 V at a constant current of 2.56 mA cm^{-2} and then followed by potentiostatic deposition at 2.05 V.

Material characterization: The crystal structure was analyzed using a Bruker D8 Advance XRD instrument. Atomic-resolution imaging was conducted using a Thermo Fisher Spectra 300 STEM with double aberration correction, operated at 300 kV. The instrument was equipped with a Super-X EDS detector for elemental analysis. The 3D plotting of Co and Bi atoms was carried out using the Matplotlib Python library. Similarly, atomic detection in the HAADF image was performed using the Laplacian of Gaussian function from the Scikit-image library. The sulfur content in the cathode was quantified using PerkinElmer TGA 4000 from 50°C to 400°C under an N_2 atmosphere, with a heating rate of 5°C min^{-1}. The adsorption performance of electrode materials on polysulfides was studied using a Lambda 950 UV-Vis-NIR spectrophotometer from Perkin Elmer.

XAS: All the XAS data were acquired at the CLAESS beamline of CELLS-ALBA synchrotron light source. Data processing for *ex situ* measured spectra of TM-Bi/CN electrodes, including

energy calibration and normalization, was performed Athena module DEMETER software package. Operando data or Co-edge and S K-edge were analyzed using original Python scripts utilizing the xralarch library. Extended X-ray absorption fine structure (EXAFS). Spectra were obtained by subtracting the background function from the overall absorption, normalizing the data with respect to the edge-jump step, and Fourier transforming the $\chi(k)$ data into real (R) space using Hanning windows ($dk = 1.0$ Å^{-1}). This process separated the contributions from different coordination shells. Standard 1st shell Fourier analysis. The fit of EXAFS data was performed with the ARTEMIS module of the DEMETER software package. To extract quantitative structural parameters such as interatomic distances, theoretical models were used to simulate the EXAFS signal was calculated using FEFF software implemented in the same package. Principal components analysis (PCA) for Co/CN and Co-Bi/CN operando datasets was performed within PyFitit software.[1] The minimal number of principal components was selected to 2. The decision was made based on using scree plot, factor indicator function (IND), the embedded error function (IE), and the Vogt–Mizaikoff F-test (Figure S30). The multivariate curve resolution (MCR) approach was used to decompose the dataset described above into pure MCR components with the original Python script utilizing the pymcr library.

Operando XRD: *Operando* XRD patterns were conducted at the MSPD beamline of CELLS-ALBA synchrotron light source to investigate the polysulfide conversion process under operating conditions. The experiments were performed under 29 keV using a CR2032 coin cell, which featured a 4 mm hole, purchased from KIT. Kapton film was applied to seal the holes in the negative and positive caps, preventing electrolyte evaporation and ensuring stable electrochemical operation throughout the measurement.

Operando XAS: *Operando* Co-K edge XAS measurements were conducted at the CLAESS beamline of CELLS-ALBA synchrotron light source to investigate the electronic structure of Co under operating conditions. The experiments were performed in fluorescence mode using a CR2032 coin cell, which featured a 4 mm hole, purchased from KIT. To ensure full penetration of the X-ray beam and enable real-time monitoring of electrochemical reactions, three 4-mm diameter holes were precisely punched into the negative and positive caps, as well as the spacer. Additionally, Kapton film was applied to seal the holes in the negative and positive caps, preventing electrolyte evaporation and ensuring stable electrochemical operation throughout the measurement.

DFT calculation: First-principles calculations were performed using DFT in the Vienna Ab initio simulation package (VASP). The electron exchange and correlation effects were treated using the generalized gradient approximation (GGA) in the Perdew-Burke-Ernzerhof (PBE) pseudopotential parameterization. The projector augmented wave (PAW) method was employed with a plane wave cutoff energy of 500 eV. Doping effects were simulated using a 2×2×1 TM-Bi/CN supercell. A vacuum slab of 16 Å was included to prevent interlayer interactions. Structural relaxation was performed using a 2×2×1 gamma-centered k-point mesh with a cutoff energy of 500 eV. The convergence criteria for energy and force were set to 1×10^{-5} eV and 0.05 eV/Å, respectively. The adsorption energy (ΔEad) was calculated as follows:

$$\Delta E_{ad} = E_{\text{surf+ad}} - E_{\text{surf}} - E_{\text{ad}}$$

where $E_{\text{surf+ad}}$ is the energy of LiPS adsorbed on the surface, E_{surf} is the energy of the clean surface, and E_{ad} is the energy of free LiPS. In addition, the differential spin density was calculated to examine the spin electron transfer during the adsorption process. The differential electron density ($\Delta\rho$) was determined using the following formula:

$$\Delta\rho = \rho_{AB} - \rho_{A} - \rho_{B}$$

where ρ_{AB} is the spin density of the system after adsorption, and ρ_{A} and ρ_{B} are the spin densities of the adsorbed species and catalyst, respectively.

The calculation formula of Gibbs free energy is:

$$\Delta G(S_8\text{-}Li_2S_8) = E_{Li_2S_8} - E_{S_8} - 2E_{Li^+}$$

$$\Delta G(Li_2S_8\text{-}Li_2S_6) = E_{Li_2S_6} - E_{Li_2S_8} + 0.25E_{S_8}$$

$$\Delta G\ (Li_2S_6\text{-}Li_2S_4) = E_{Li_2S_4} - E_{Li_2S_6} + 0.25E_{S_8}$$

$$\Delta G(Li_2S_4\text{-}Li_2S_2) = E_{Li_2S_2} - E_{Li_2S_4} + 0.25E_{S_8}$$

$$\Delta G(Li_2S_2\text{-}Li_2S) = E_{Li_2S} - E_{Li_2S_2} + 0.125E_{S_8}$$

Supplementary Data

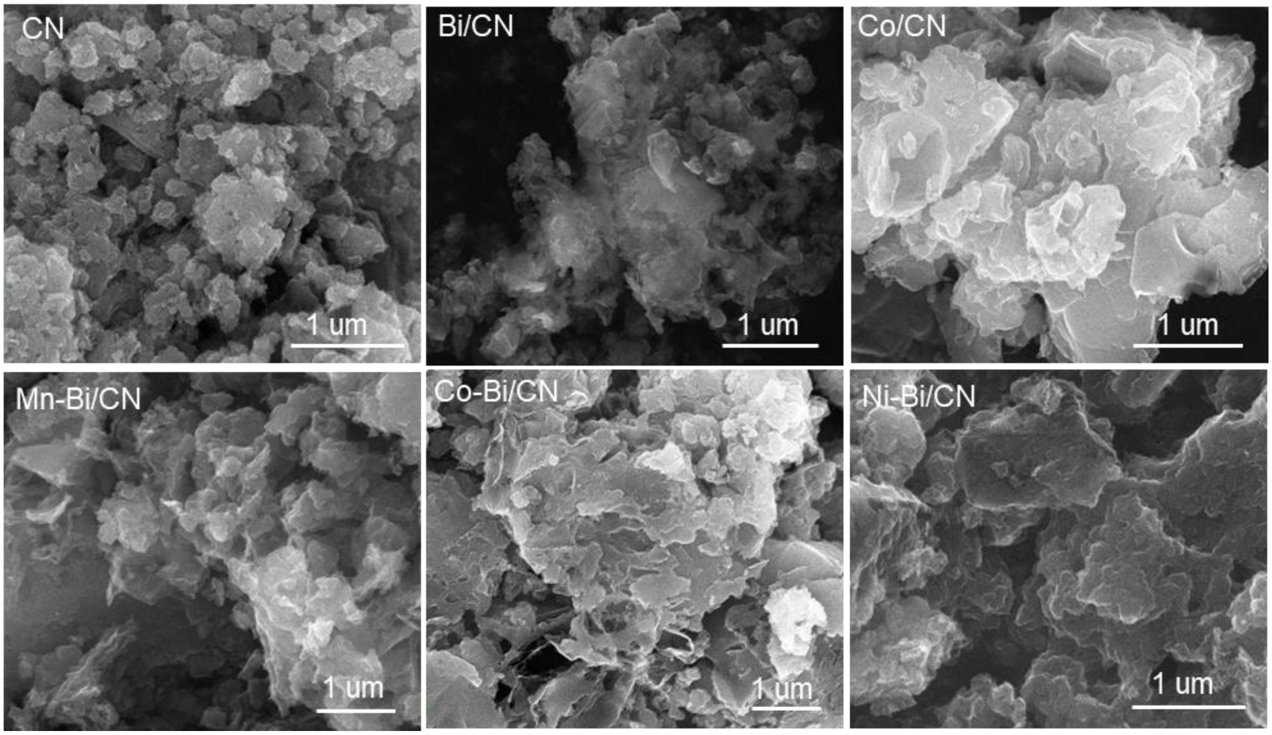

Figure S1. SEM images of CN, Bi/CN, Co/CN, Mn-Bi/CN, Co-Bi/CN, and Ni-Bi/CN.

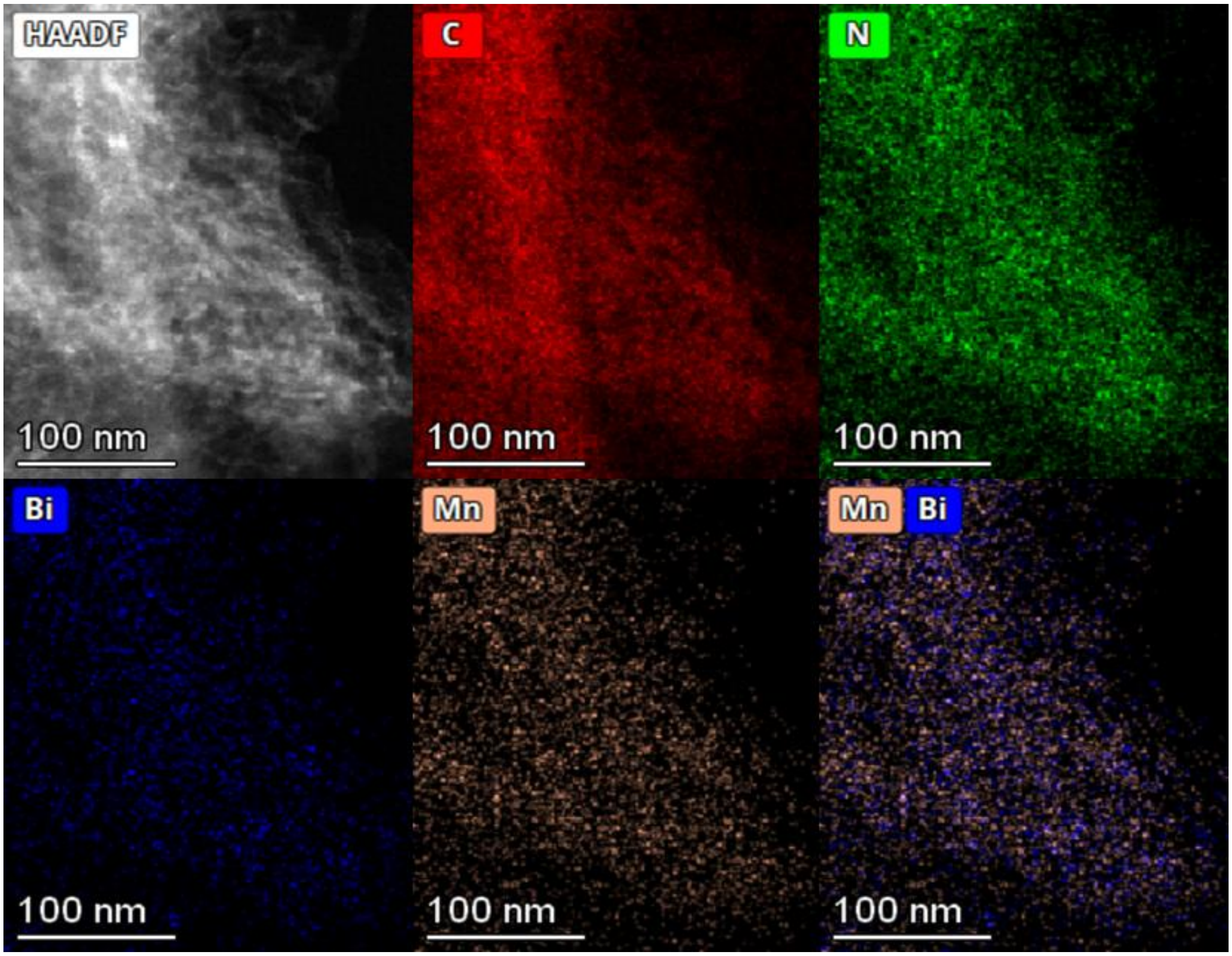

Figure S2. STEM-HAADF image and corresponding EDS elemental maps of Mn-Bi/CN.

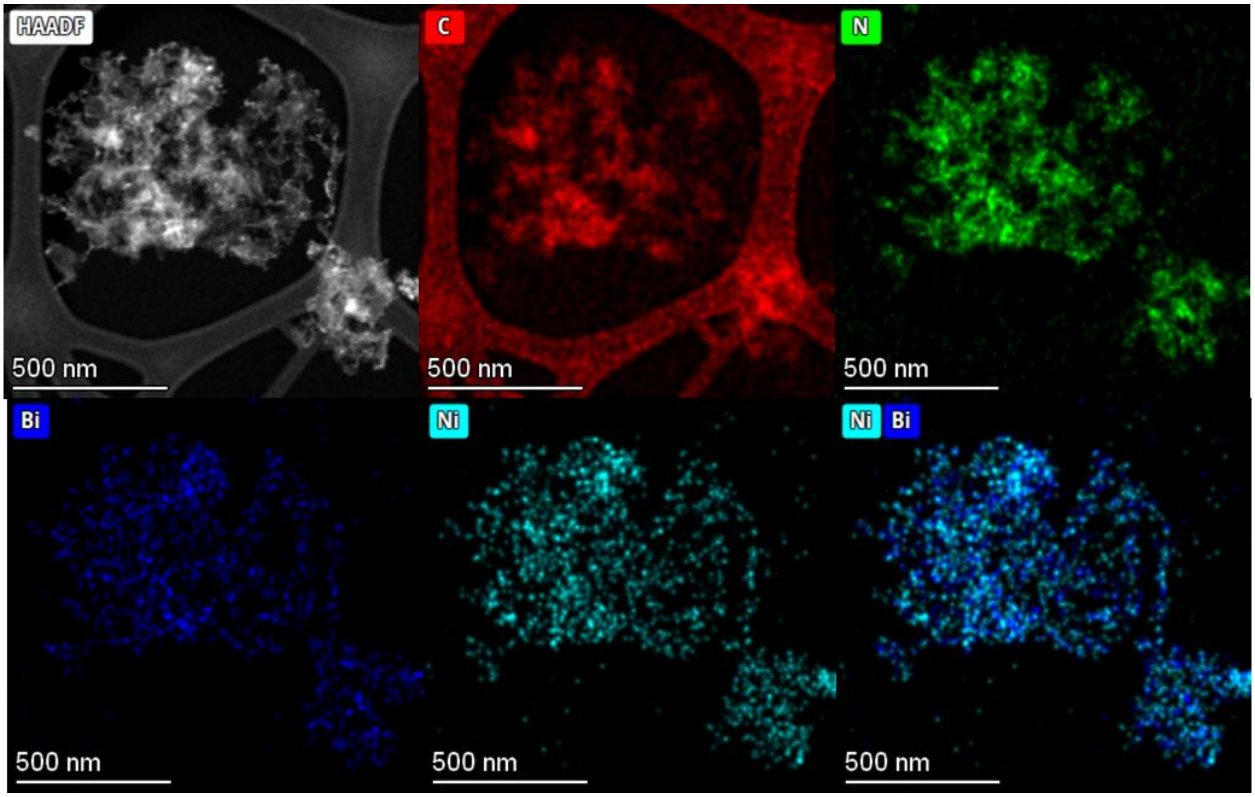

Figure S3. STEM-HAADF image and corresponding EDS elemental maps of Ni-Bi/CN.

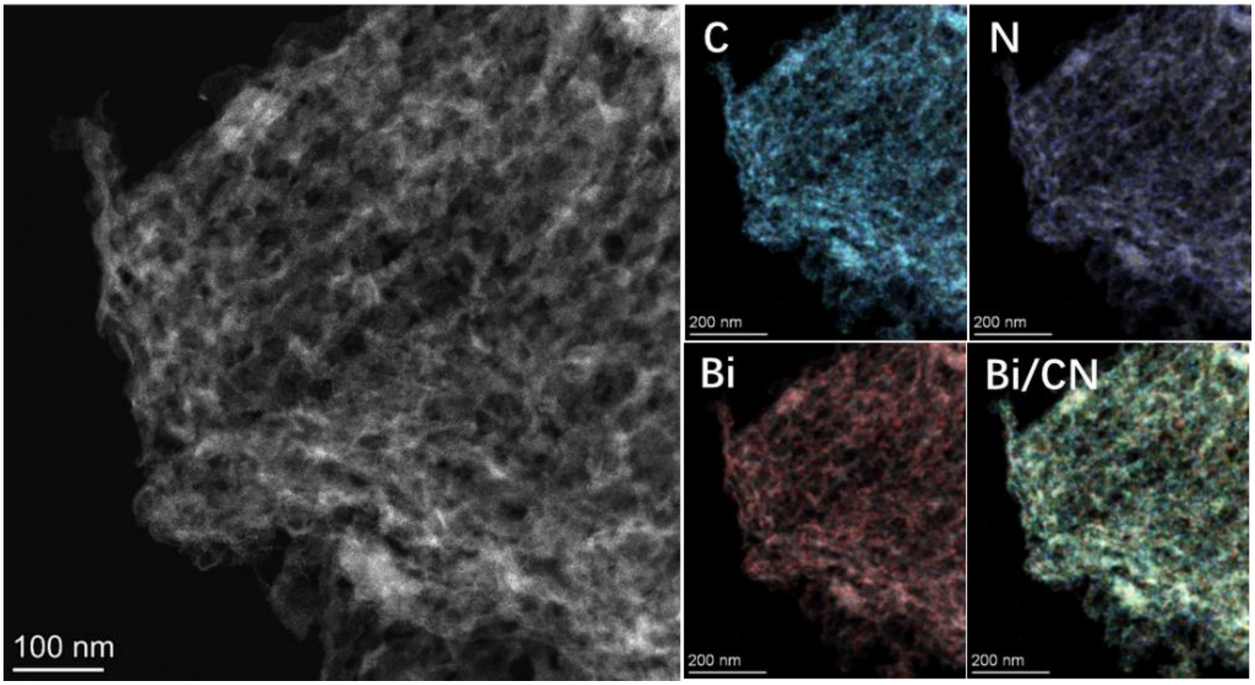

Figure S4. STEM-HAADF image and corresponding EDS elemental maps of Bi/CN.

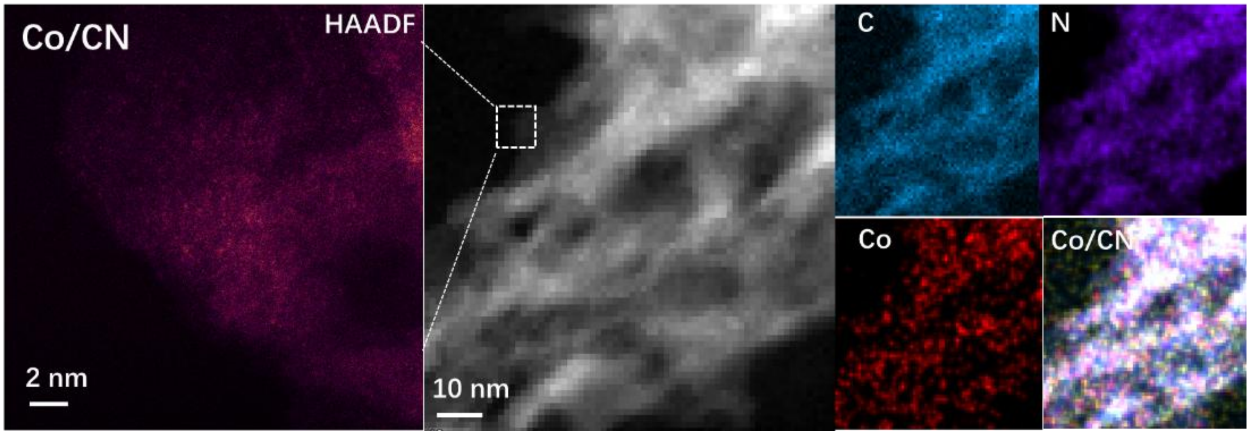

Figure S5. HAADF-STEM images (false-color) and STEM-EDS elemental maps of Co/CN.

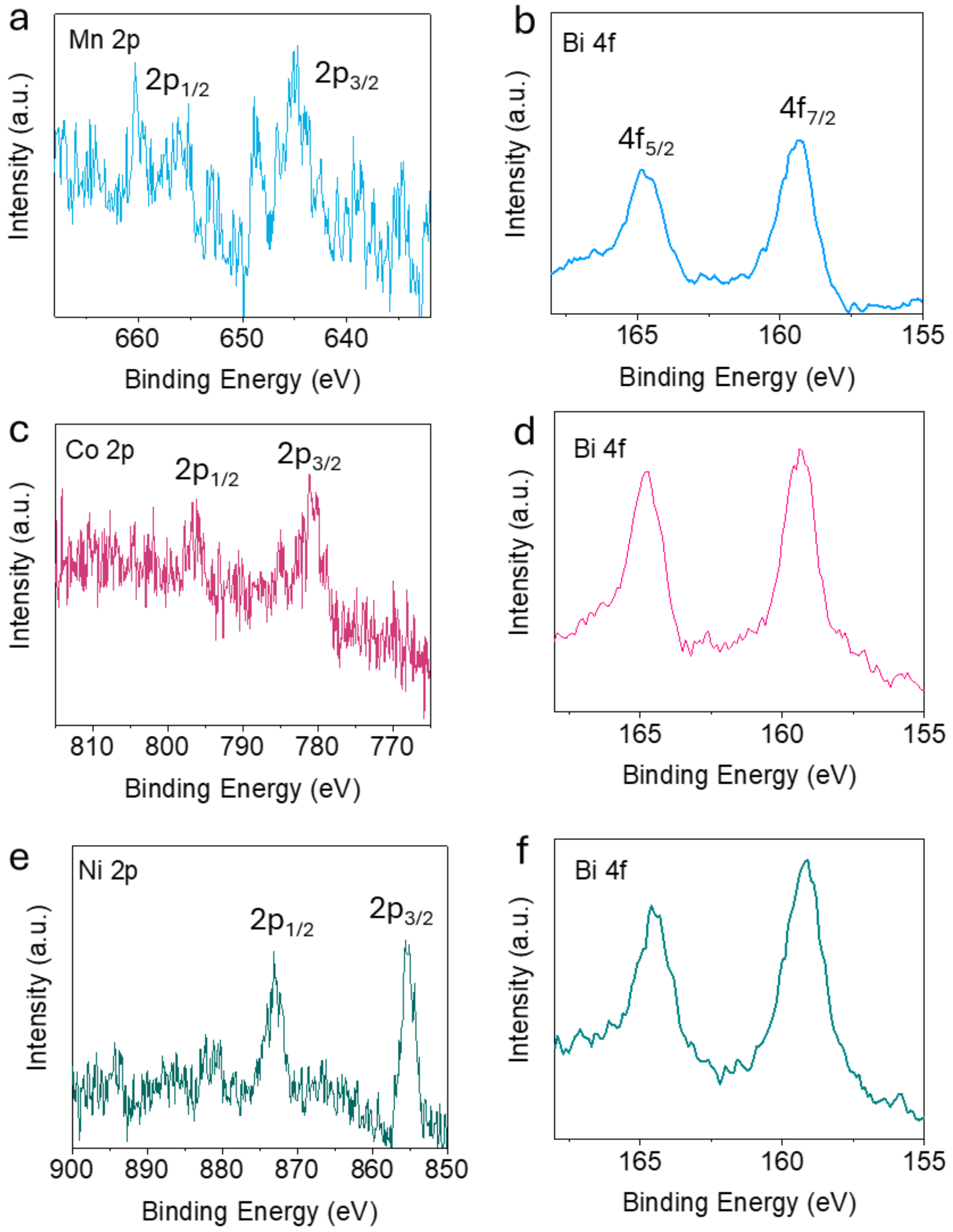

Figure S6. (a,b) High-resolution Mn 2p (a) and Bi 4f (b) XPS spectra of Mn-Bi/CN. (c,d) High-resolution Co 2p (c) and Bi 4f (d) XPS spectra of Co-Bi/CN. (e,f) High-resolution Ni 2p (e) and Bi 4f (f) XPS spectra of Ni-Bi/CN.

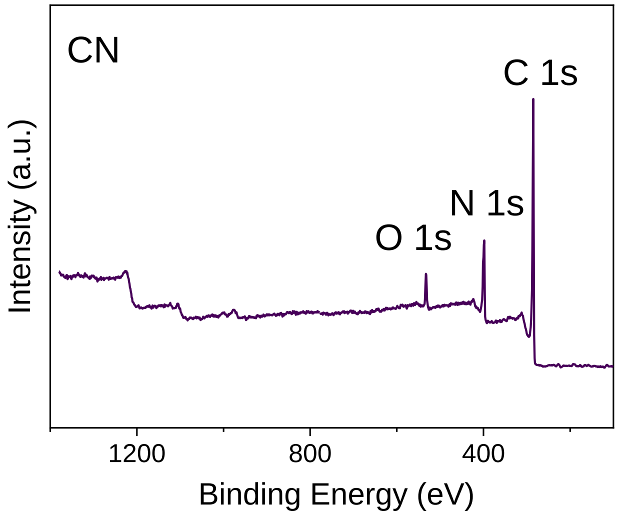

Figure S7. Survey XPS spectrum of the CN sample showing the absence of transition metals and Bi elements.

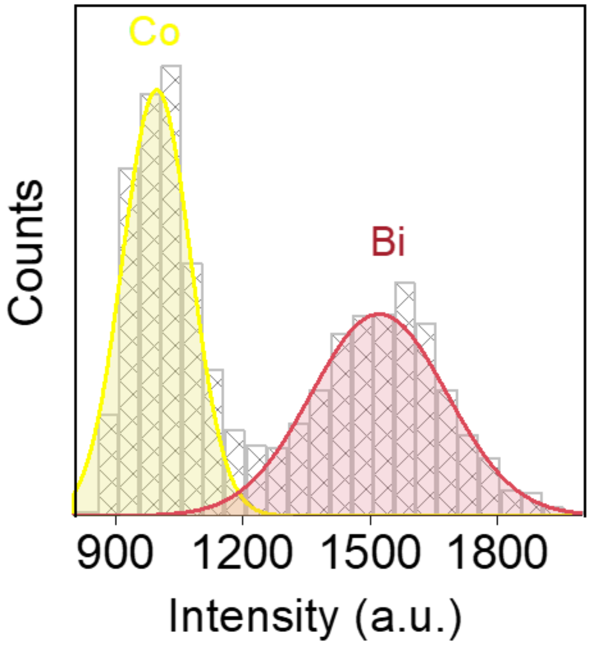

Figure S8. Histogram of all the intensity spikes detected in the Co-Bi/CN AC HAADF STEM image using Laplacian of Gaussian algorithm.

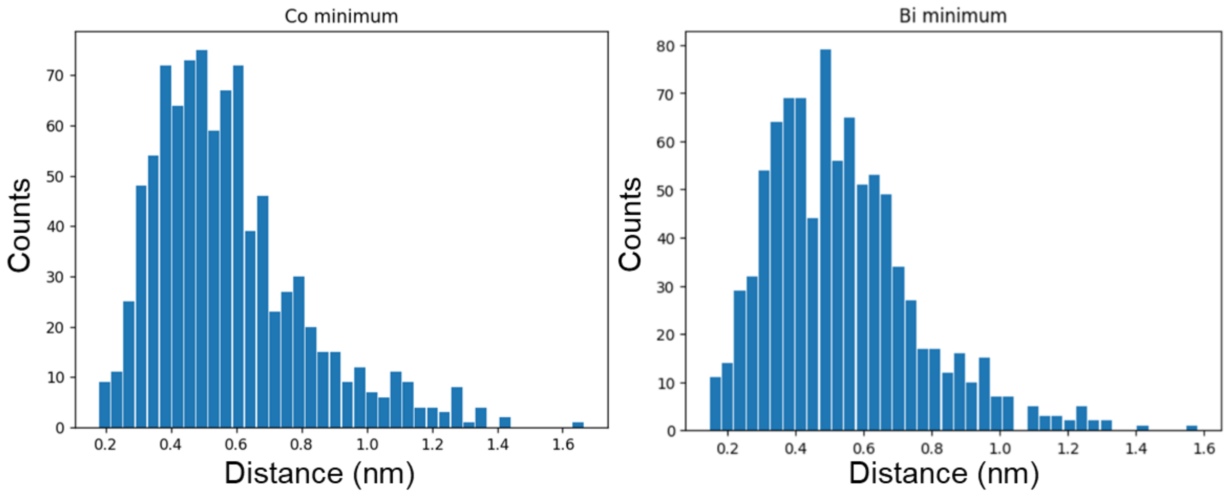

Figure S9. Histograms of the minimum distance's distribution from Co and Bi atoms within Co-Bi/CN

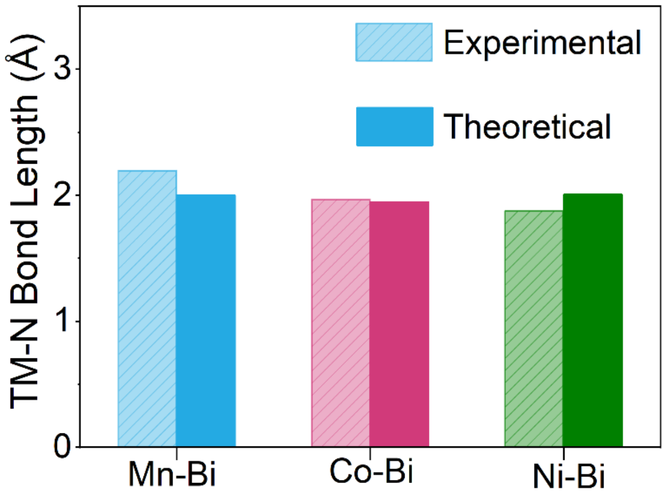

Figure S10. Comparison of the TM-N bond length experimentally obtained with that calculated from the DAC model (TM-Bi/CN).

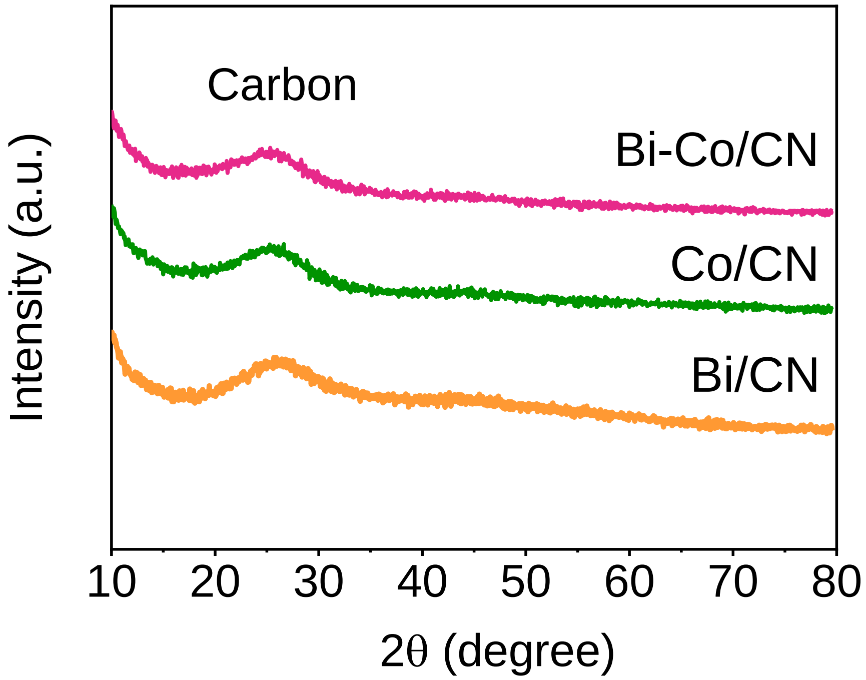

Figure S11. XRD patterns of Bi/CN, Co/CN and Co-Bi/CN.

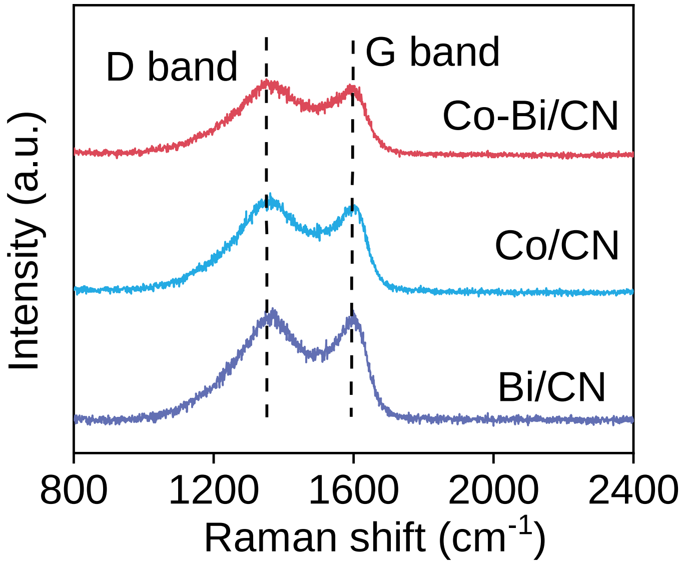

Figure S12. Raman spectra of Bi/CN, Co/CN, and Co-Bi/CN samples. The results indicate that the D band and G band remain unaffected by the anchored atoms.

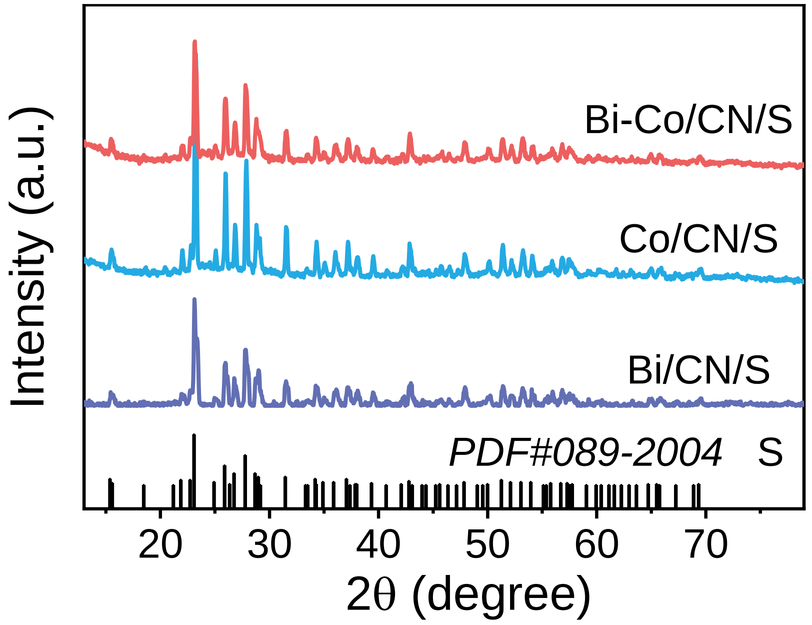

Figure S13. XRD patterns of Bi/CN/S, Co/CN/S and Co-Bi/CN/S.

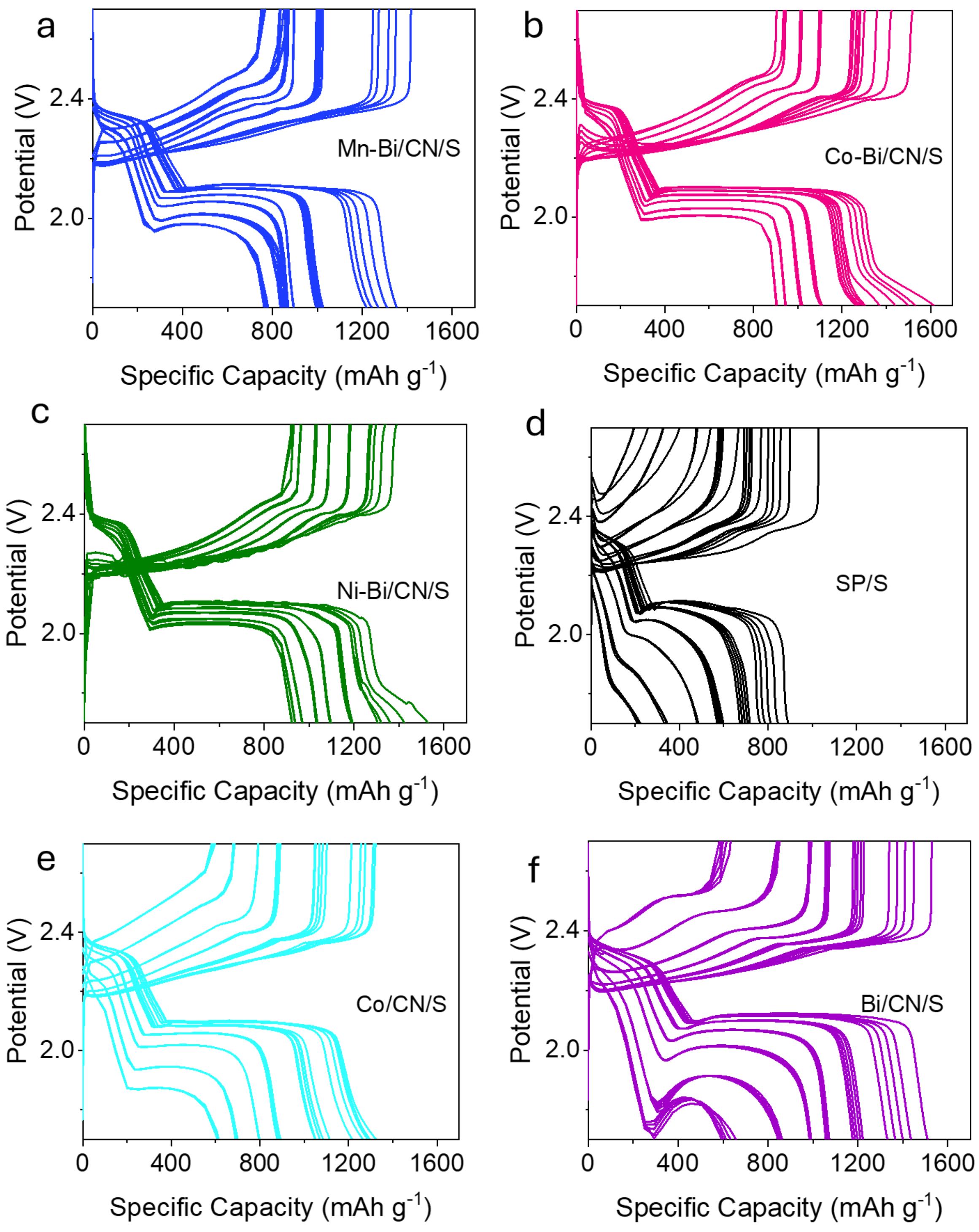

Figure S14. GCD profiles at different rates (0.1C, 0.2C, 0.5C, 1C, 2C, and 3C, for 5 cycles each) for Mn-Bi/CN/S, Co-Bi/CN/S, Ni-Bi/CN/S, SP/S, Co/CN/S, and Bi/CN/S, with the y-axis displayed over the voltage range of 1.7–2.8 V.

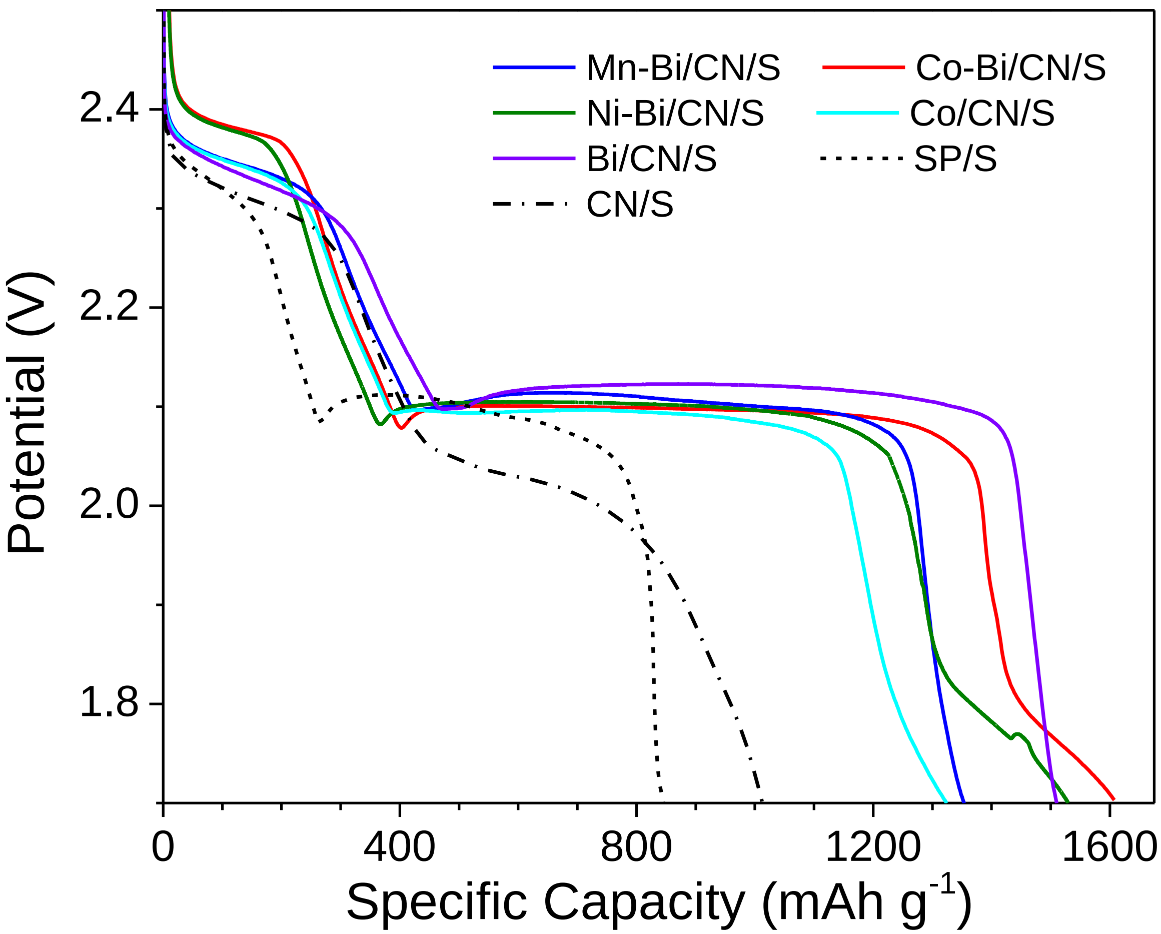

Figure S15. Galvanostatic discharge profiles of Mn-Bi/CN/S, Co-Bi/CN/S, Ni-Bi/CN/S, Co/CN/S, Bi/CN/S, SP/S, and CN/S at 0.1C, with the y-axis displayed over the voltage range of 1.7–2.5 V.

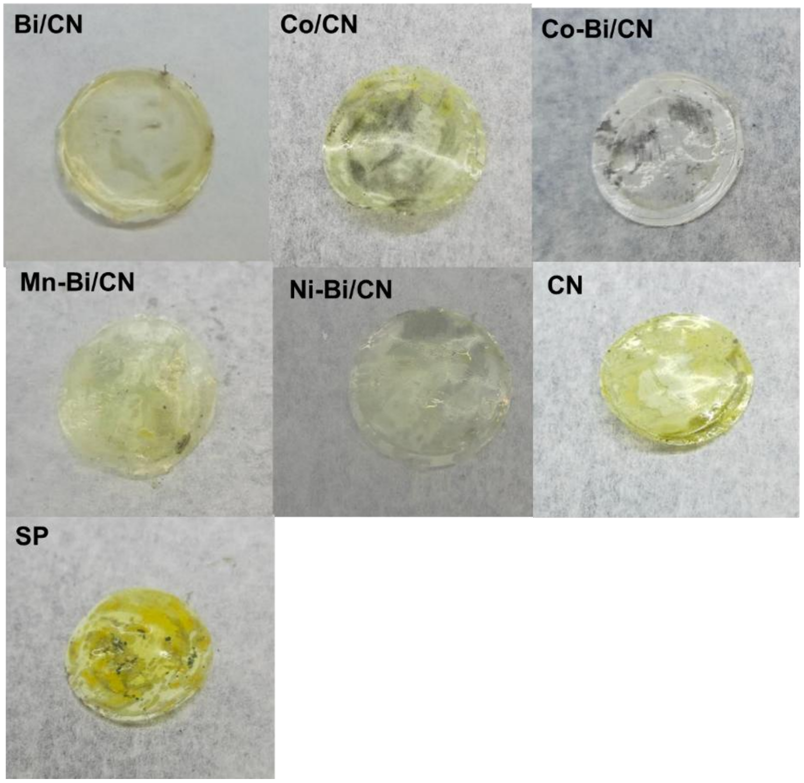

Figure S16. Digital images of coin cell separators after one charge/discharge cycle with sulfur cathodes incorporating different catalysts: Bi/CN/S, Co/CN/S, Co-Bi/CN/S, Mn-Bi/CN/S, Ni-Bi/CN/S, CN/S, and SP/S.

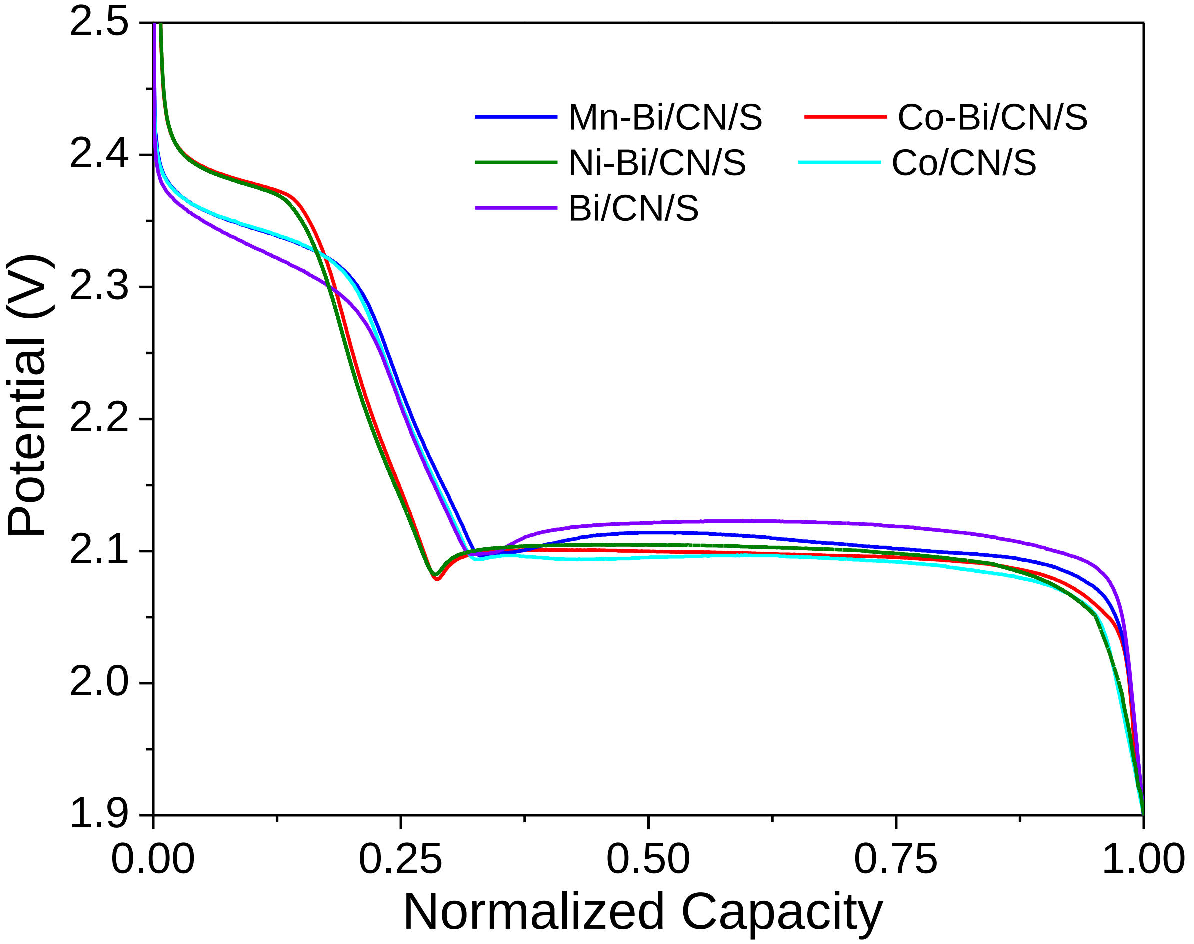

Figure S17. Normalized galvanostatic discharge curves of Mn-Bi/CN/S, Co-Bi/CN/S, Ni-Bi/CN/S, Co/CN/S, and Bi/CN/S at 0.1C, with the y-axis displayed over the voltage range of 1.9–2.5 V.

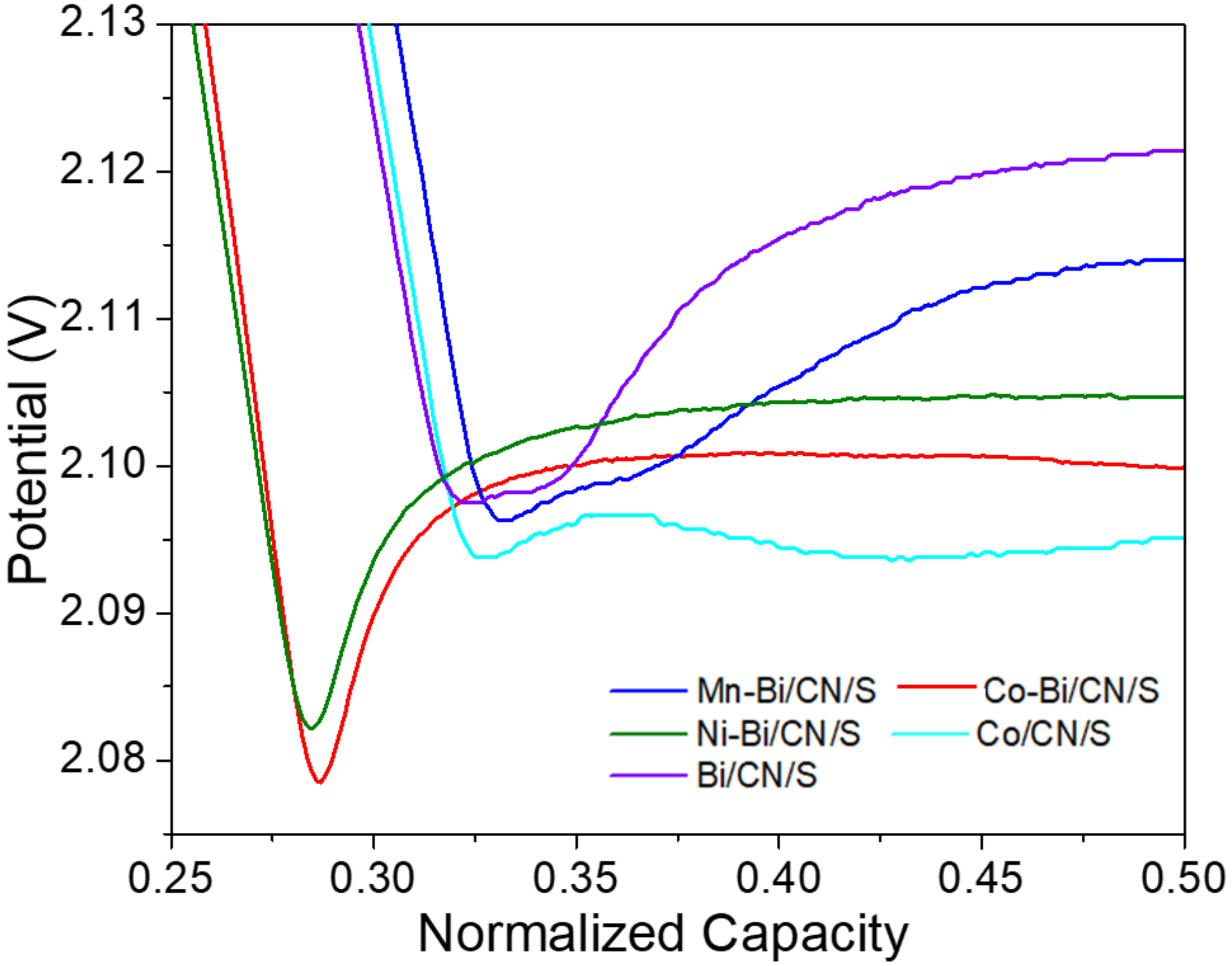

Figure S18. Galvanostatic discharge curves at 0.1C at the transition between the two plateaus.

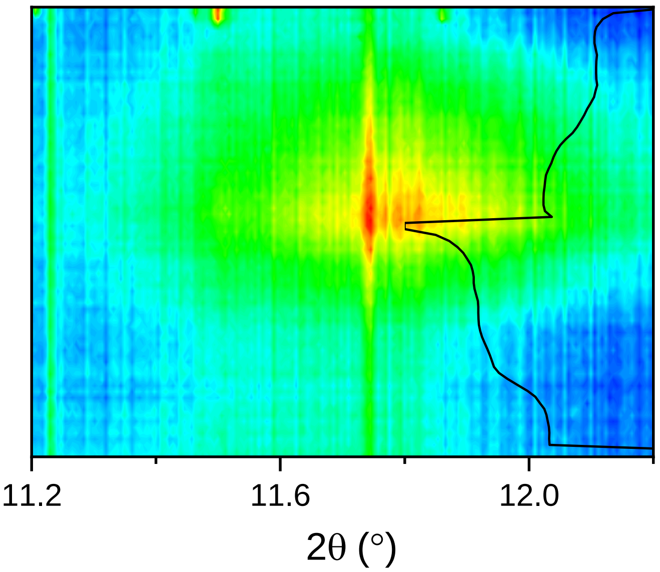

Figure S19. *Operando* XRD patterns of Mn-Bi/CN/S and GCD profile.

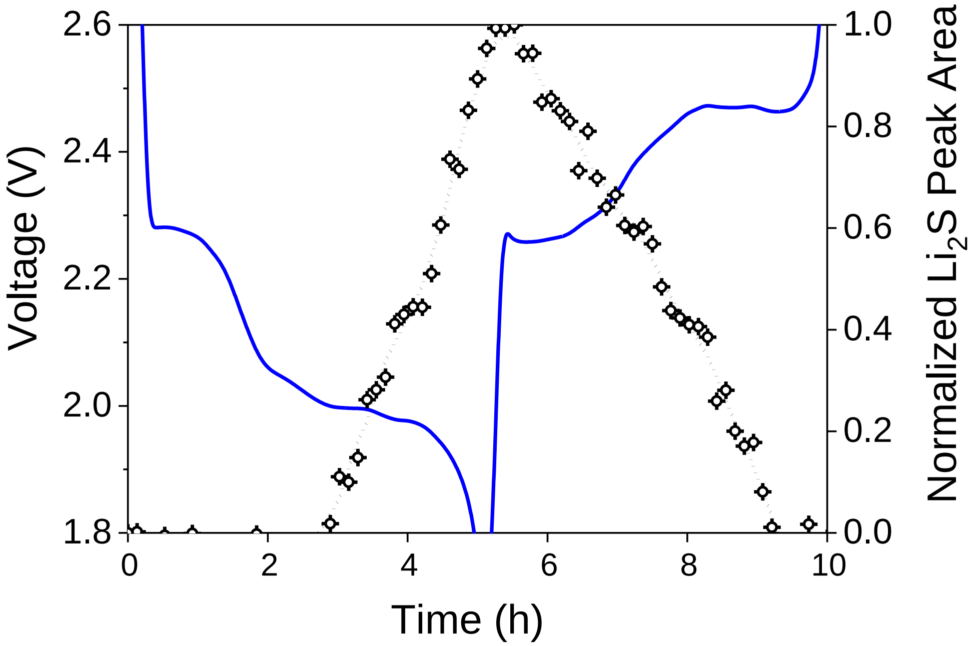

Figure S20. Integrated Li_2S (331) peak area and GCD profile for Mn-Bi/CN/S.

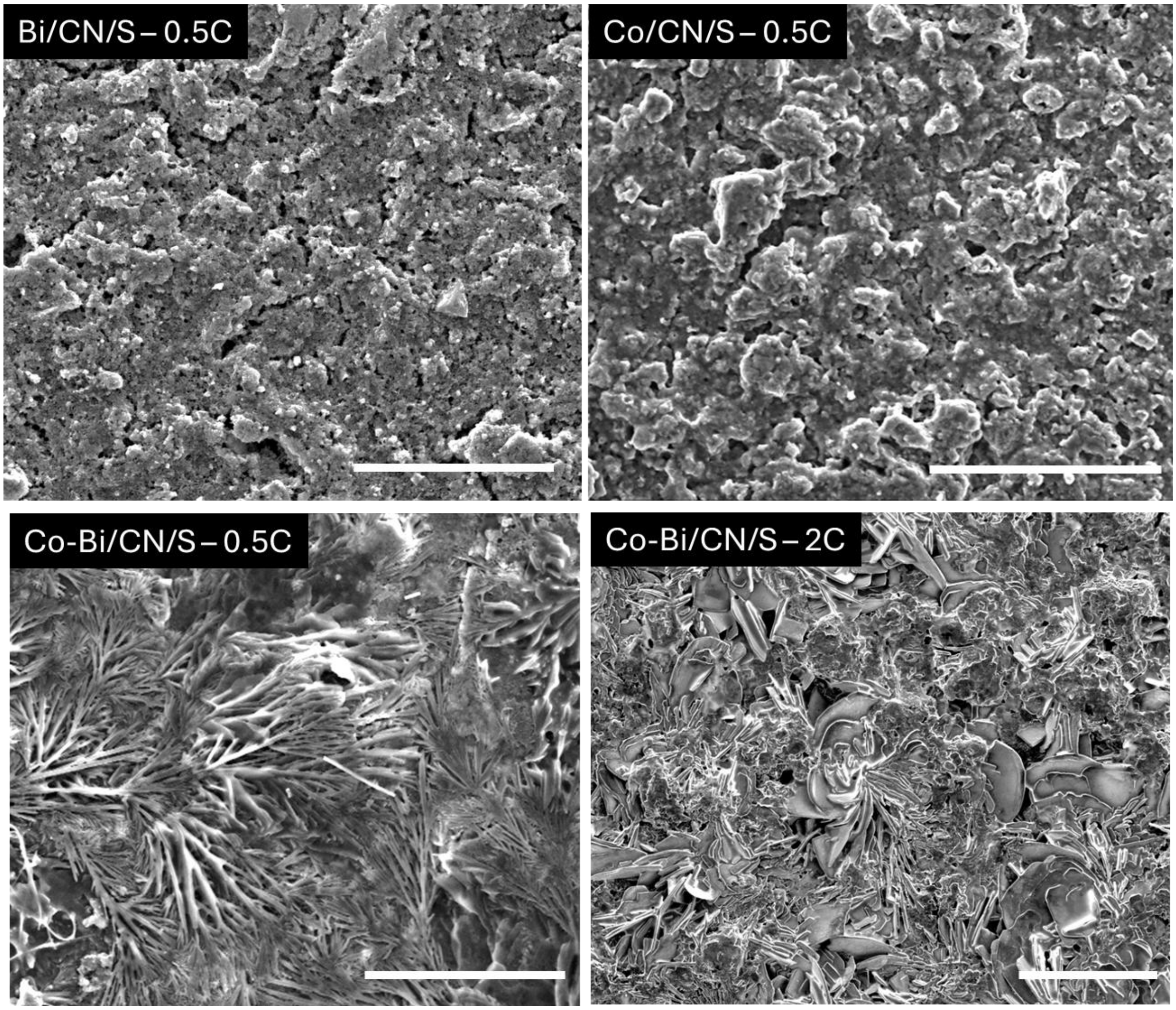

Figure S21. SEM images of Bi/CN/S, Co/CN/S, and Co-Bi/CN/S cathodes after discharging at 0.5C and a Co-Bi/CN/S cathode discharged at 2C, as noted in labels. Scale bars = 100 μm.

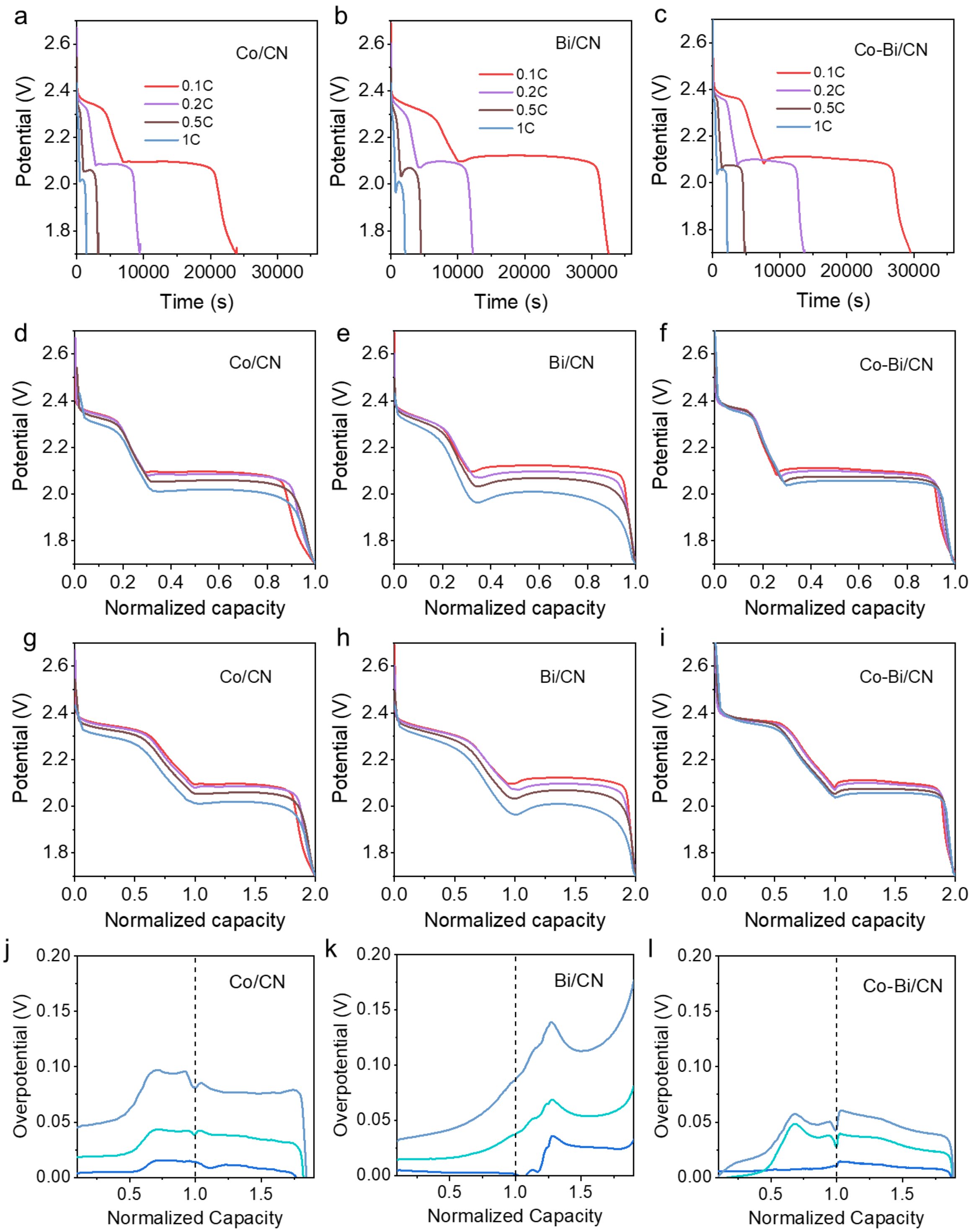

Figure S22. Galvanostatic discharge profiles (a-c), profiles normalized to the total capacity (d-f), profiles normalized for both Q_1 and Q_2 steps (g-i), and relative overpotential at 0.2C, 0.5C and 1C, referred to the 0.1C profiles (j-i) of Co/CN/S (a,d,g,j), Bi/CN/S (b,e,h,k) and Co-Bi/CN/S (c,f,i,l) based cells.

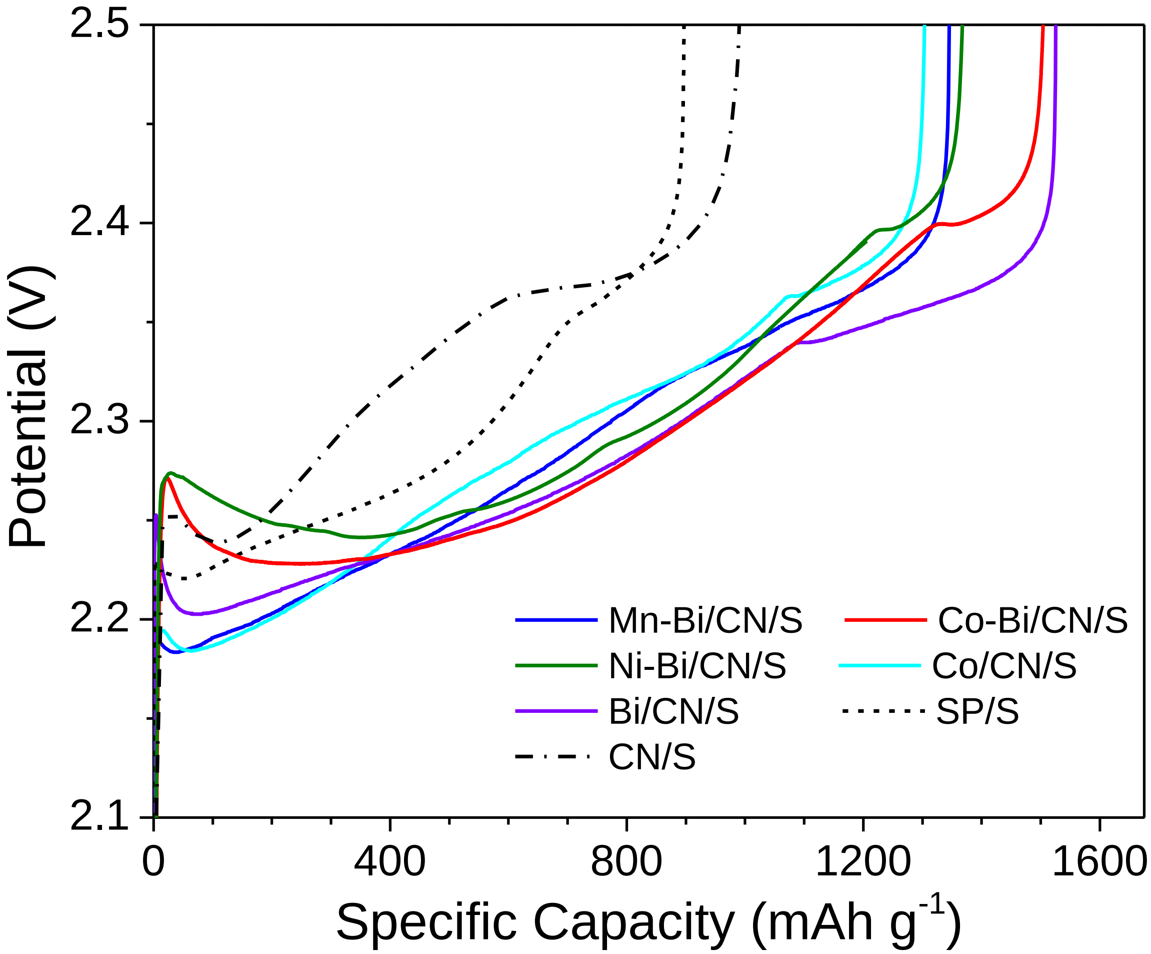

Figure S23. Galvanostatic charge curves of Mn-Bi/CN/S, Co-Bi/CN/S, Ni-Bi/CN/S, Co/CN/S, Bi/CN/S, SP/S, and CN/S at 0.1C, with the y-axis displayed over the voltage range of 2.1–2.5 V.

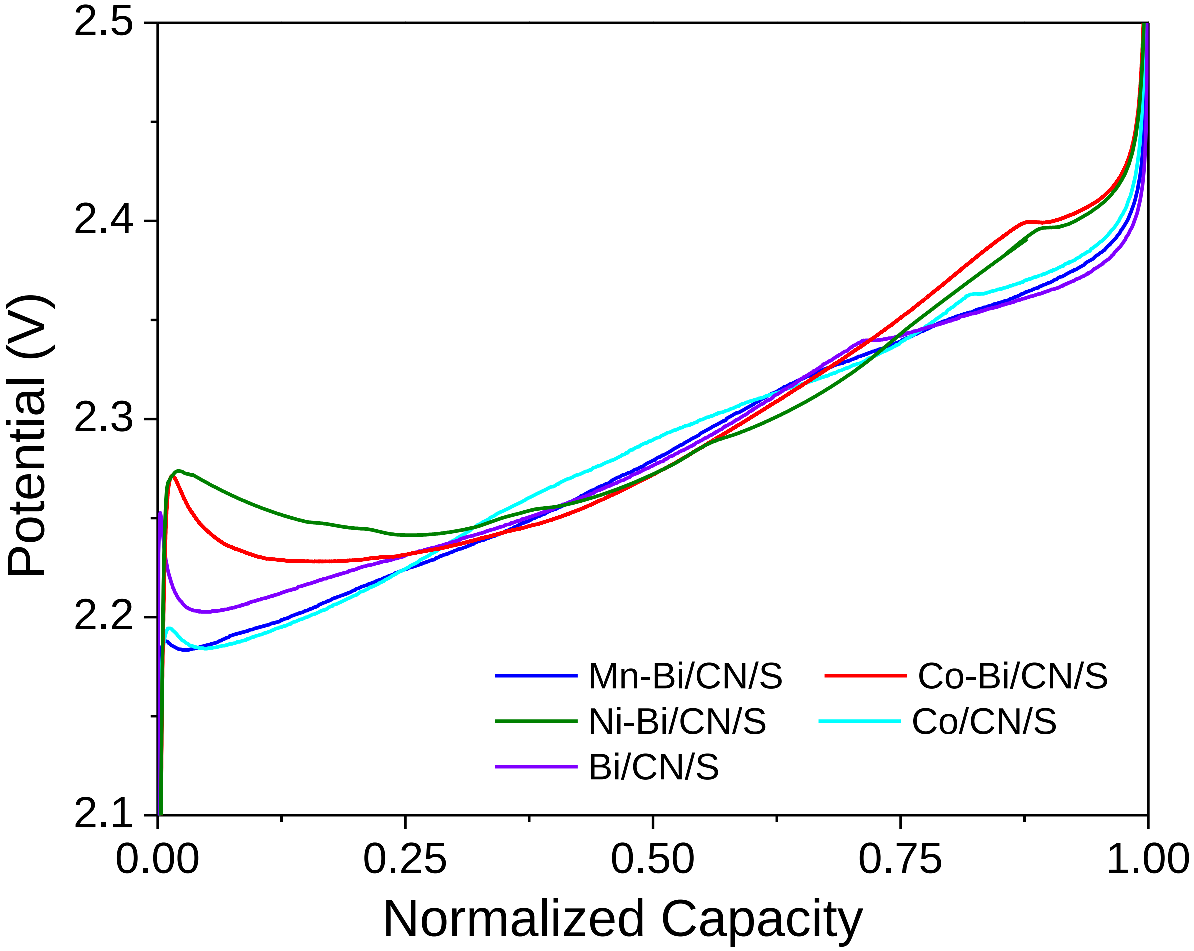

Figure S24. Normalized galvanostatic charge profiles of Mn-Bi/CN/S, Co-Bi/CN/S, Ni-Bi/CN/S, Co/CN/S, and Bi/CN/S at 0.1C, with the y-axis displayed over the voltage range of 2.1–2.5 V.

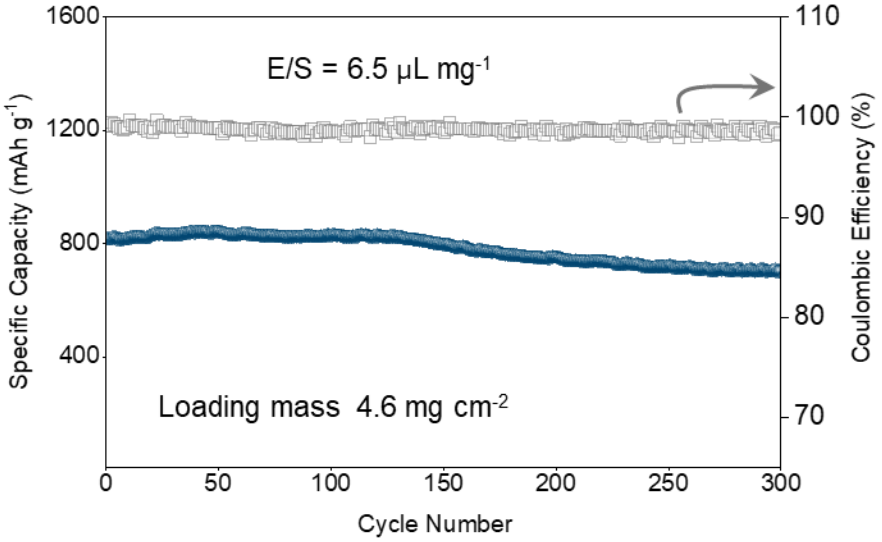

Figure S25. Cycling stability of the Co-Bi/CN/S electrode at a sulfur loading of 4.6 mg cm^{-2} and an electrolyte-to-sulfur ratio of 6.5 mL mg^{-1}, tested at 0.5C.

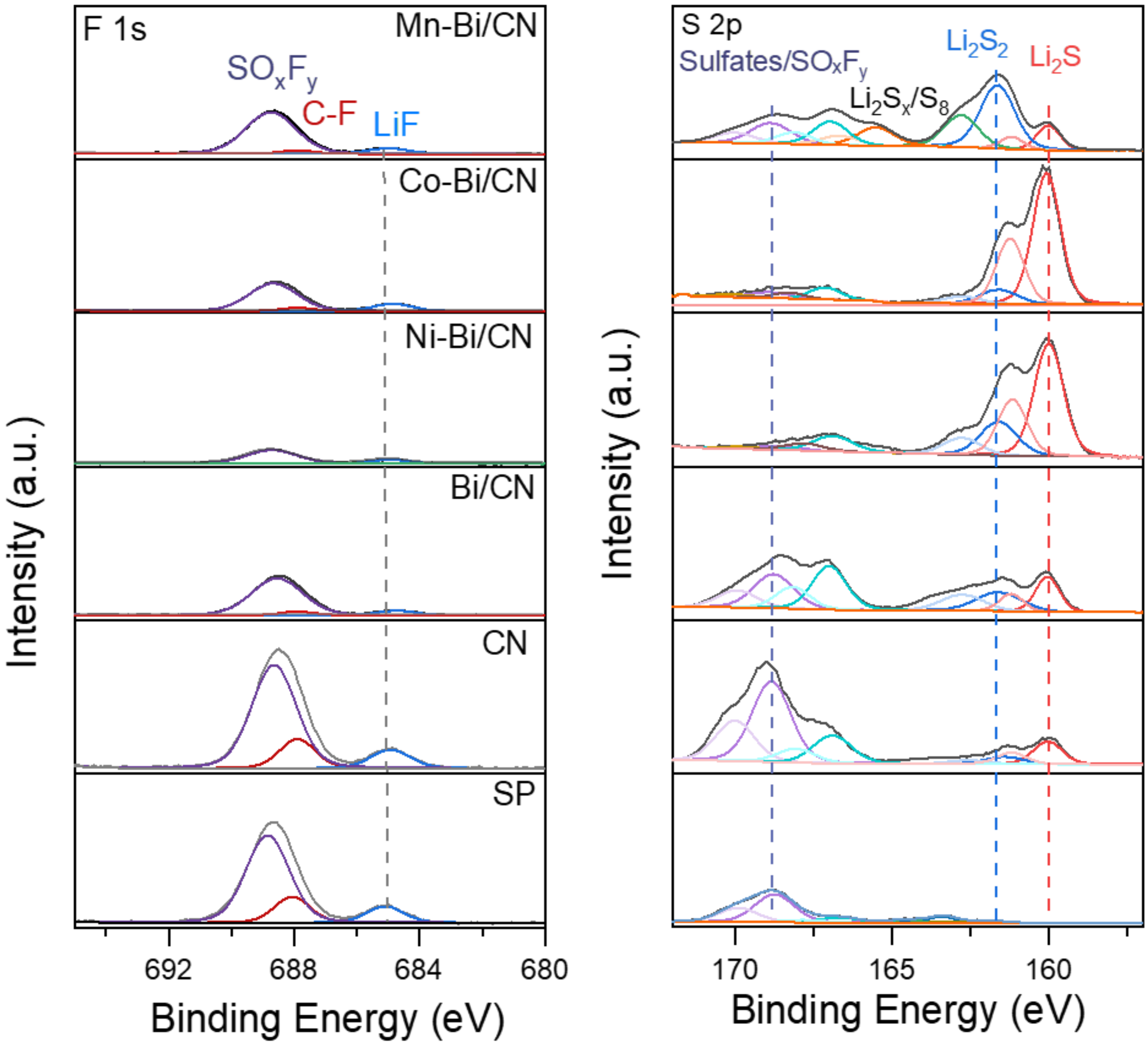

Figure S26. High-resolution F 1s (left) and S 2p (right) XPS spectra of the different electrode after 100 cycles at 1C.

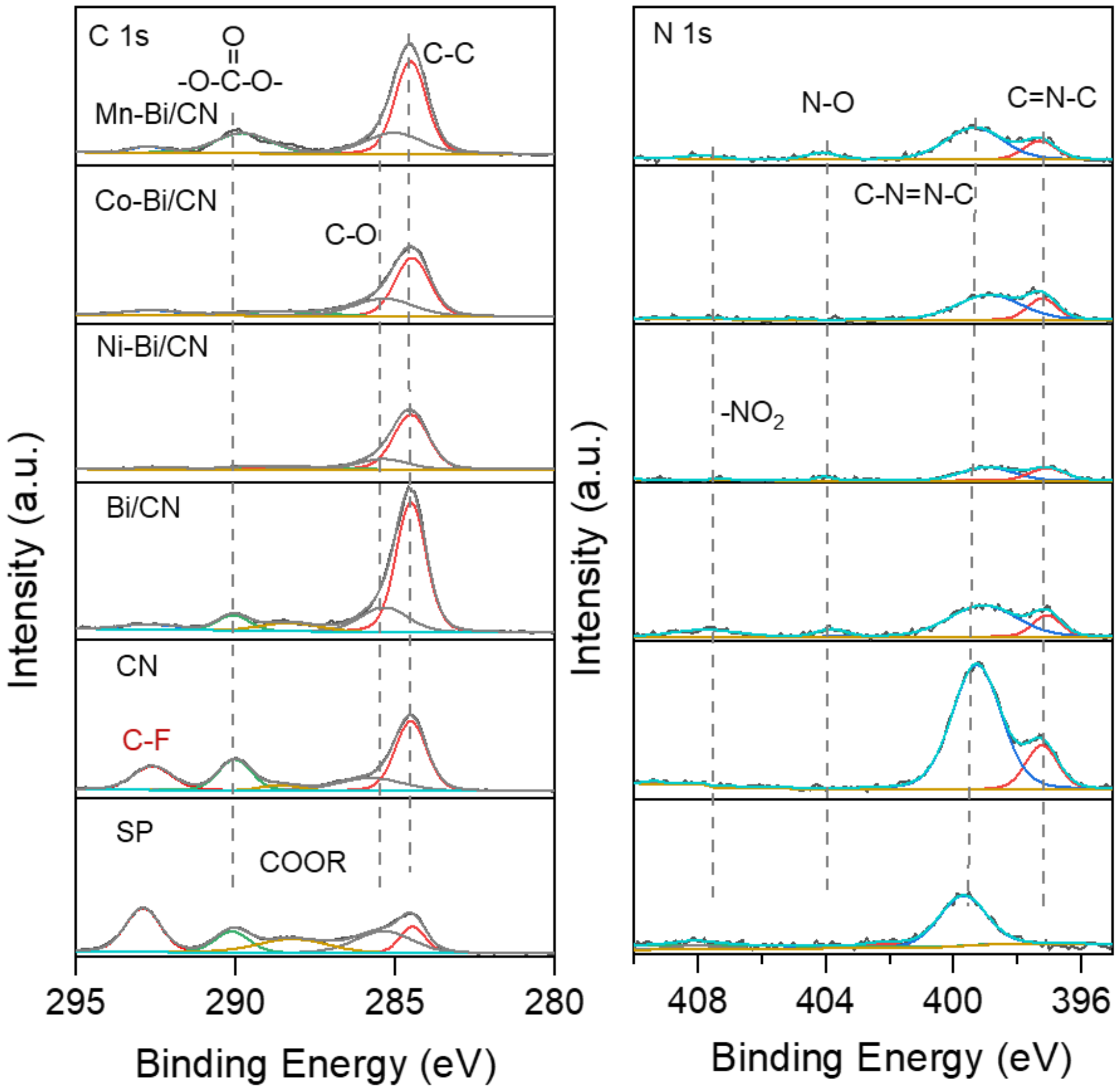

Figure S27. High-resolution C 1s (left) and N 1s (right) XPS spectra of the different electrodes after 100 cycles at 1C.

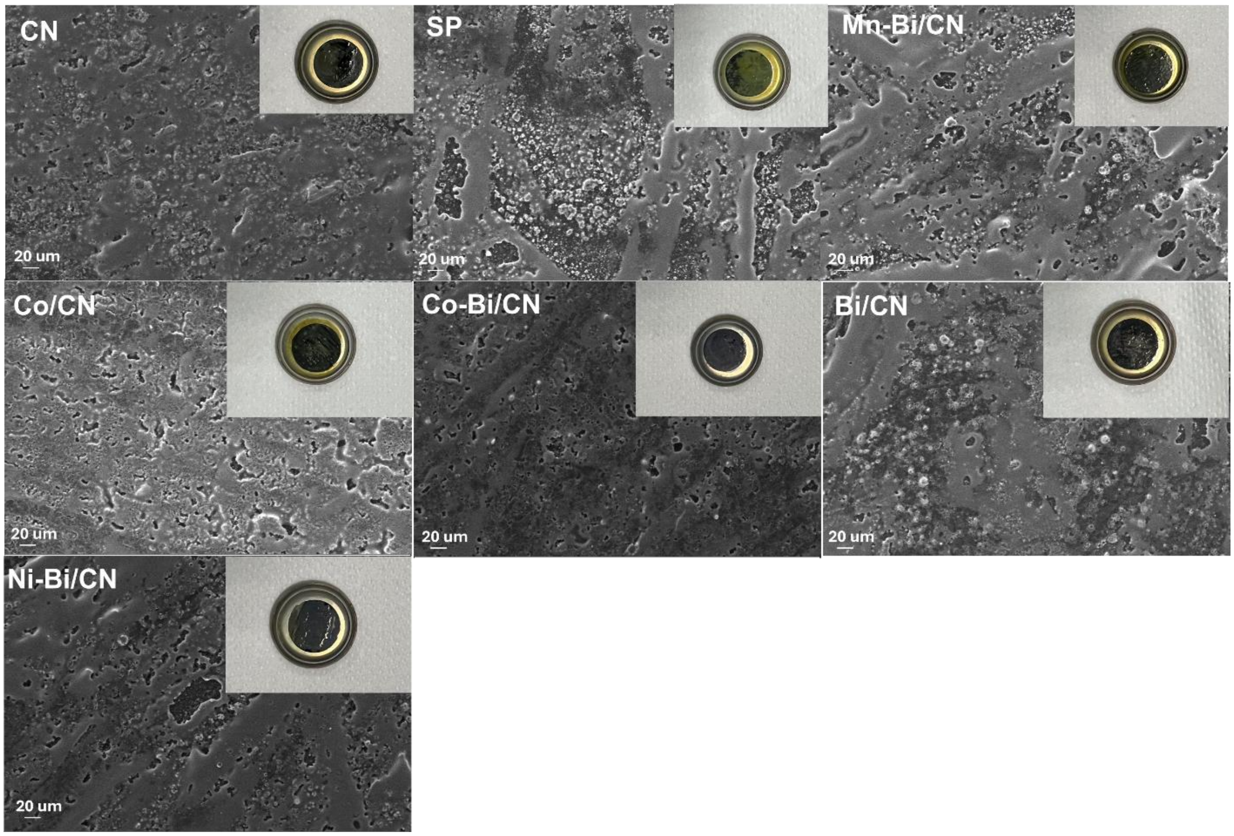

Figure S28. SEM images of Li anode and corresponding digital photographs of the Li anode with the separator after 100 charge/discharge cycles with different catalysts: Bi/CN/S, Co/CN/S, Co–Bi/CN/S, Mn–Bi/CN/S, Ni–Bi/CN/S, CN/S, and SP/S.

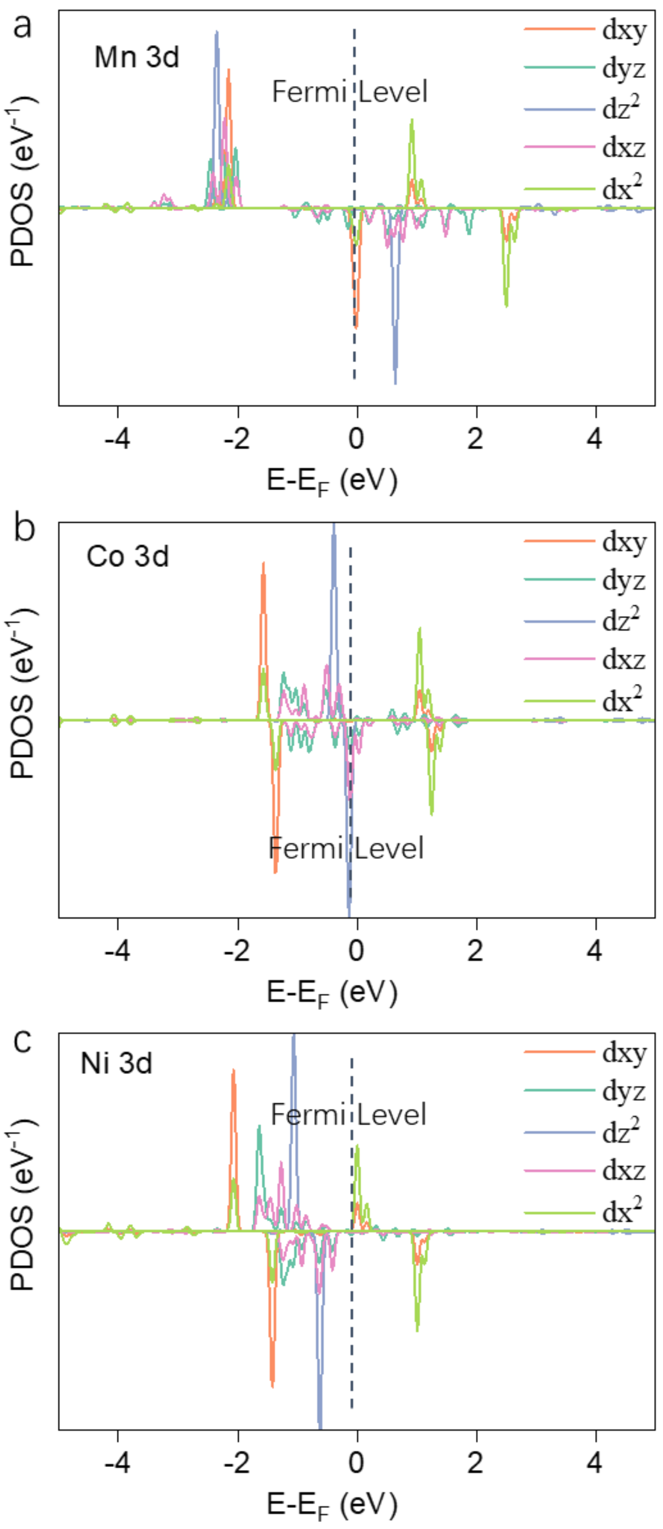

Figure S29. Partial density of states (PDOS) of 3d TM states of Mn-Bi/CN, Co-Bi/CN, and Ni-Bi/CN.

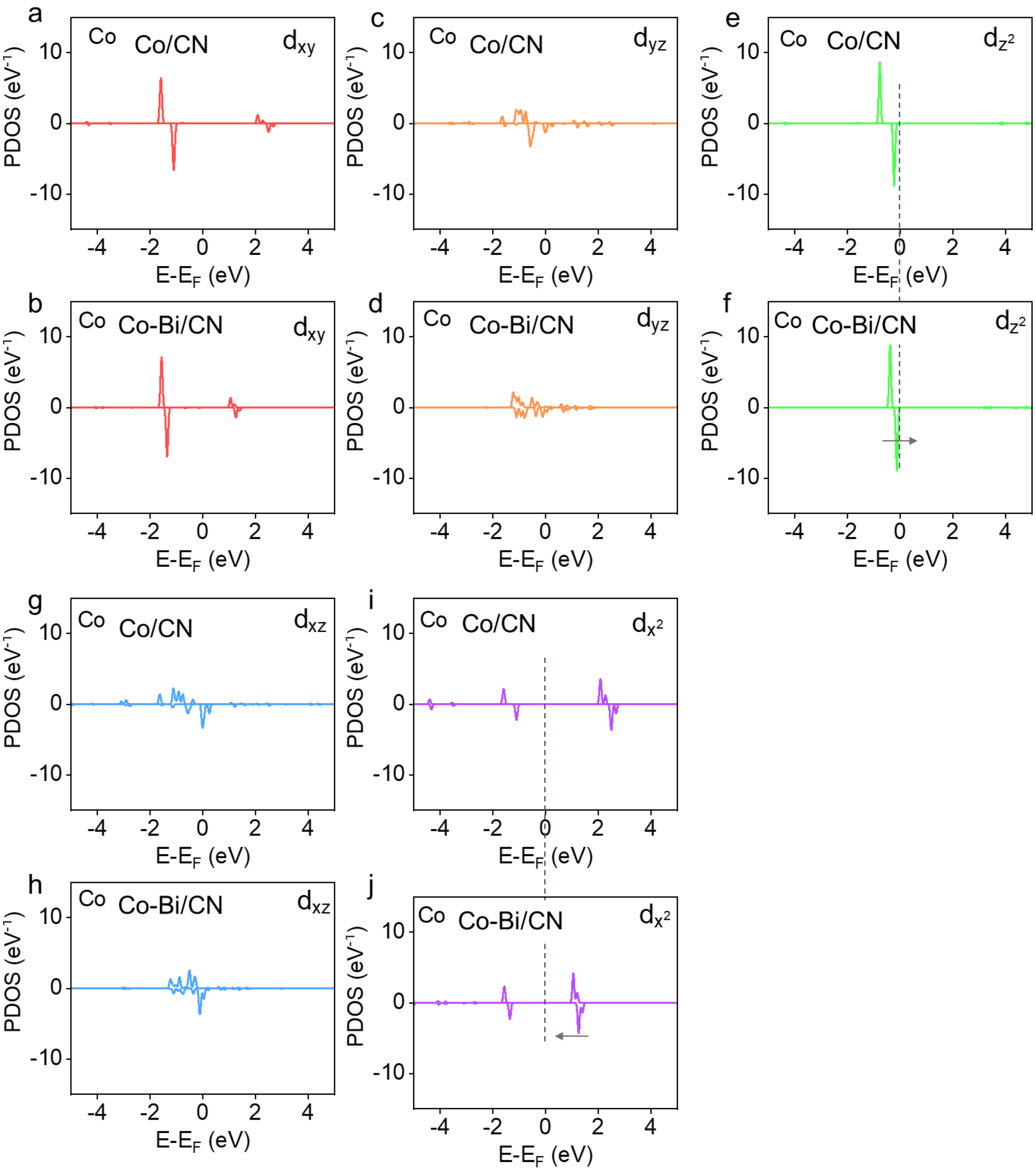

Figure S30. PDOS of d orbitals (d_{xy}, d_{yz}, d_z^2, d_{xz}, and d_x^2) of Co 3d from Co/CN and Co-Bi/CN samples.

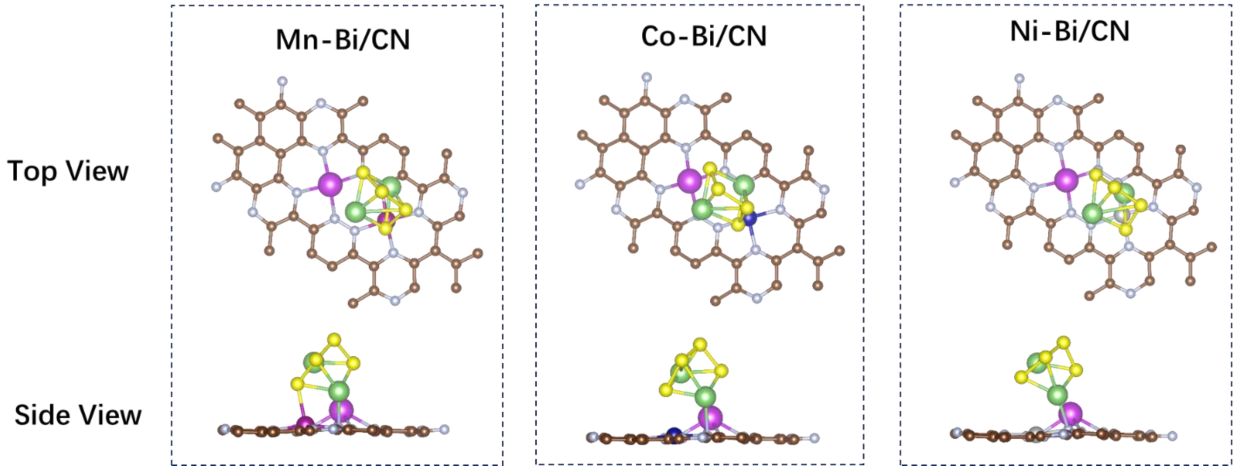

Figure S31. Top and side views of the Li_2S_4 adsorption on Mn-Bi/CN, Co-Bi/CN, and Ni-Bi/CN.

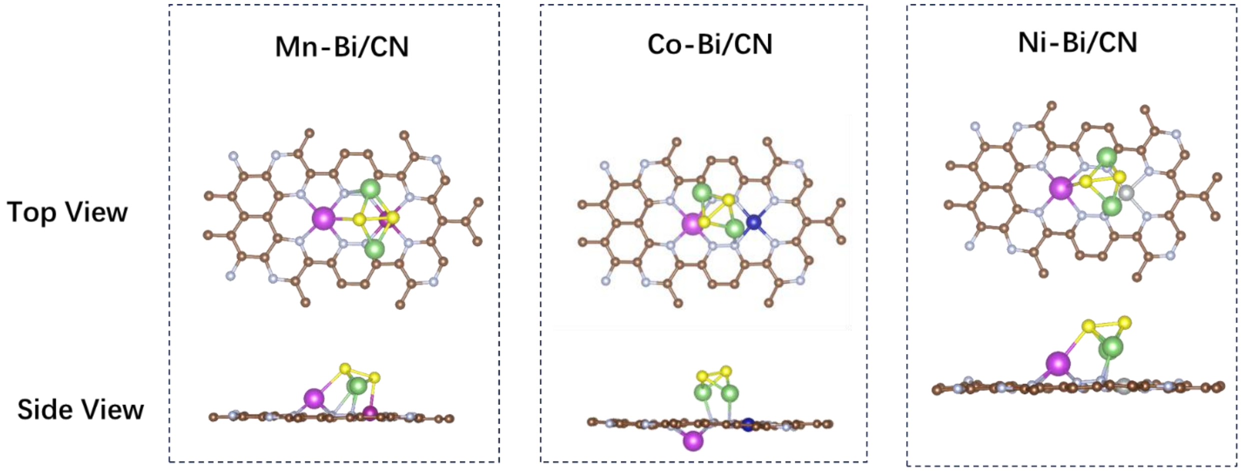

Figure S32. Top and side views of the Li_2S_2 adsorption on Mn-Bi/CN, Co-Bi/CN, and Ni-Bi/CN.

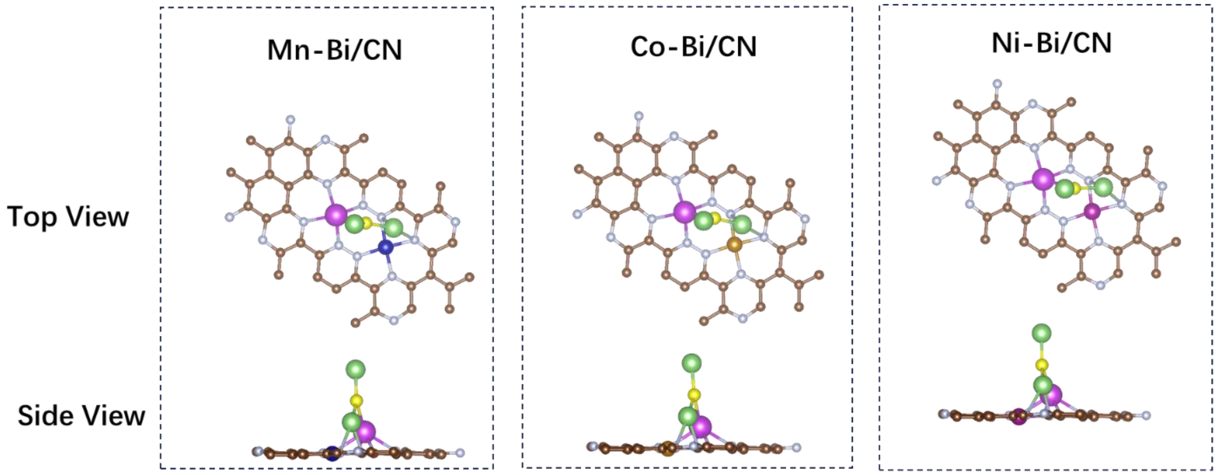

Figure S33. Top and side views of the Li_2S adsorption on Mn-Bi/CN, Co-Bi/CN, and Ni-Bi/CN.

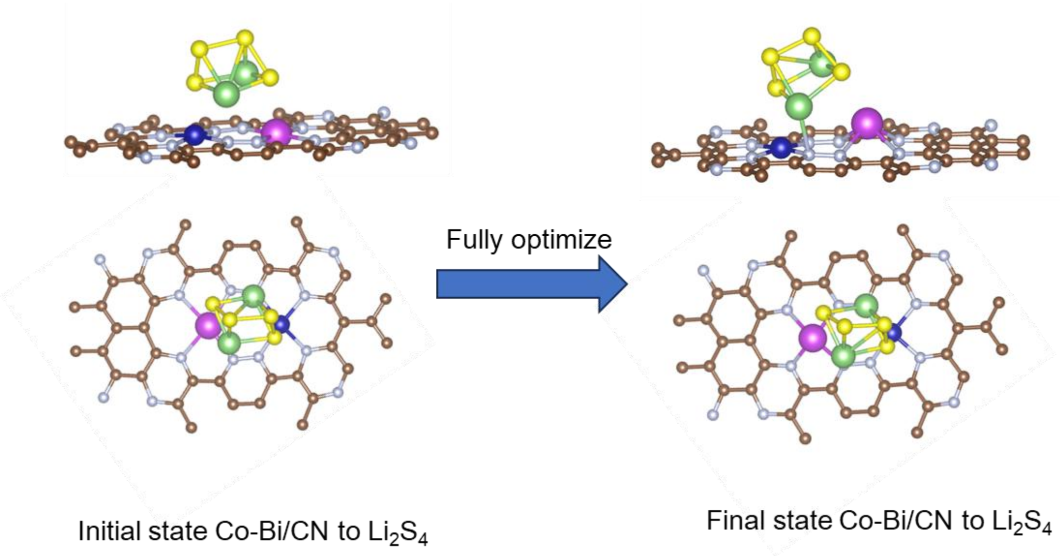

Figure S34. Top and side views of Li_2S_4 adsorption on the Co–Bi/CN surface: comparison between initial and fully relaxed structures.

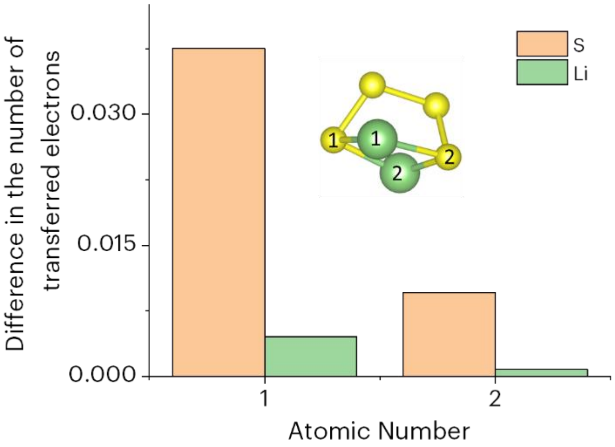

Figure S35. Number of electrons transferred by Li_2S_4 before and after adsorption on the Co-Bi catalyst surface.

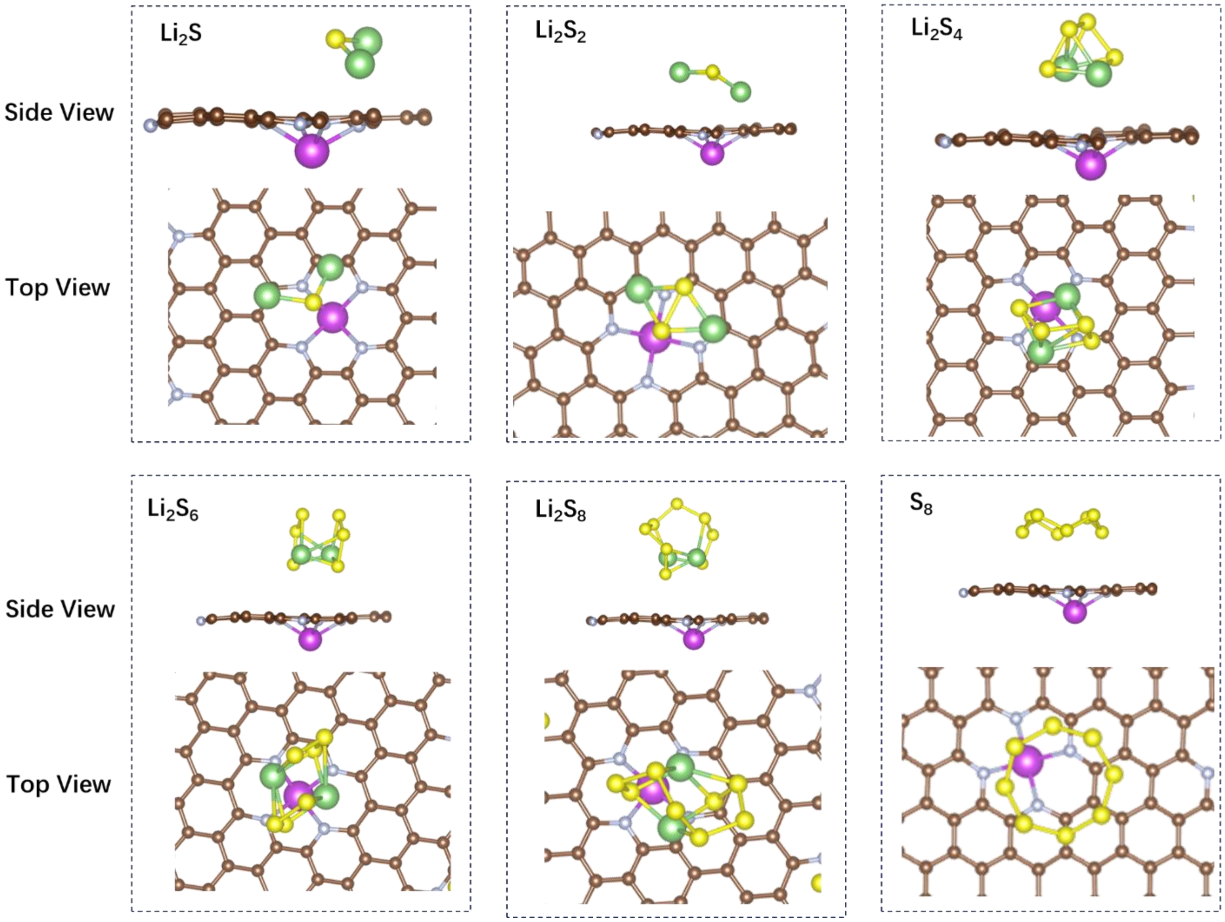

Figure S36. DFT optimized geometrical configuration of Bi/CN with LiPS.

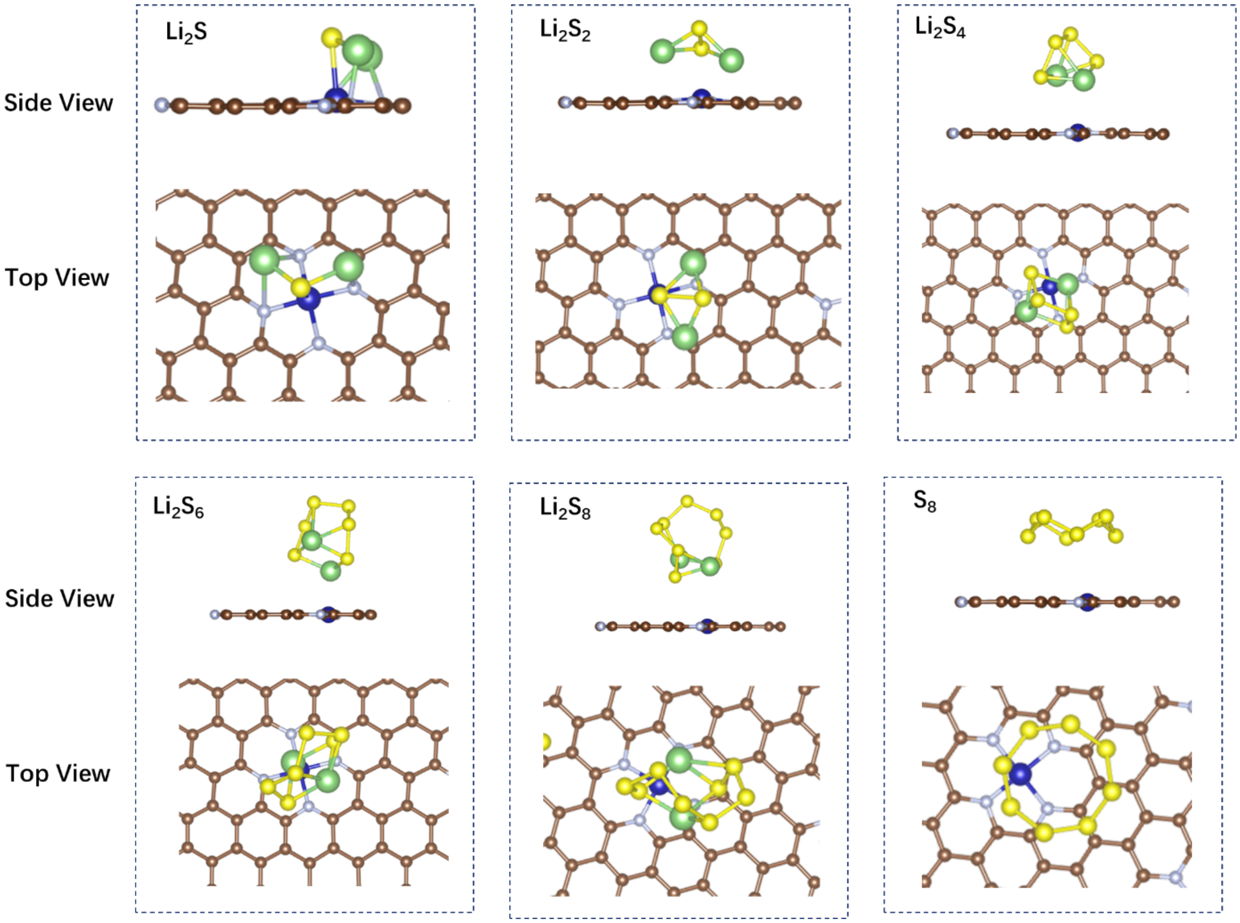

Figure 37. DFT optimized geometrical configuration of Co/CN with LiPS.

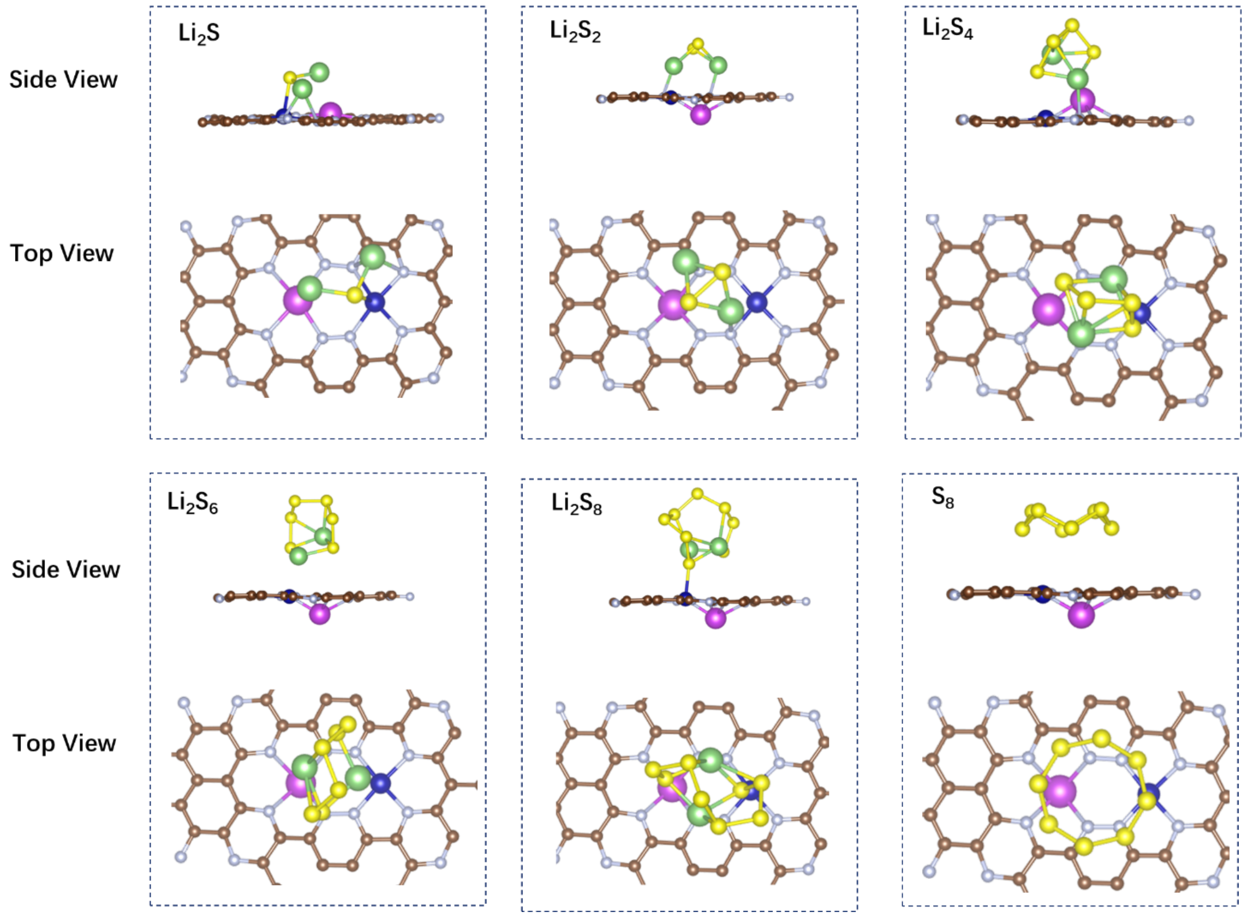

Figure S38. DFT optimized geometrical configuration of Co-Bi/CN with LiPS.

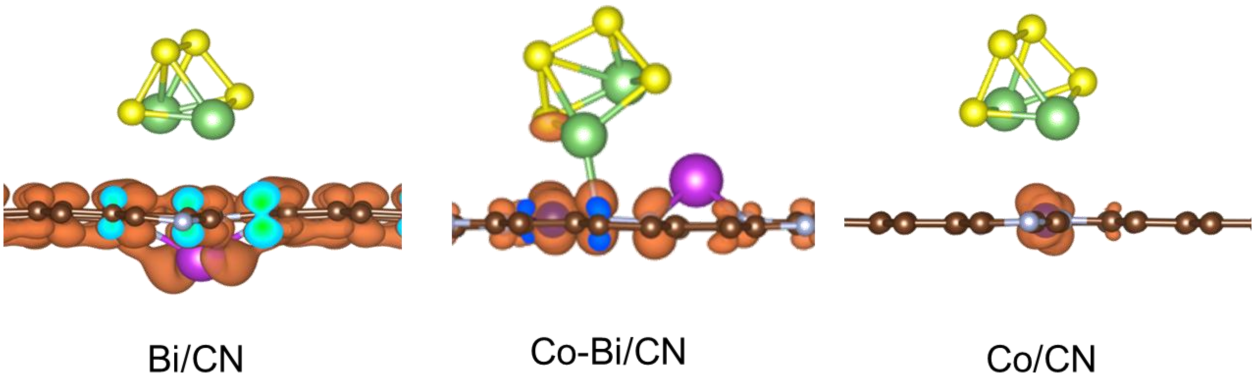

Figure S39. Side view of spin slabs of a Li_2S_4 molecule adsorbed on Bi/CN (left), Co/CN (right), and Co-Bi/CN (middle). The isosurface level is 0.001 e/a_0^3.

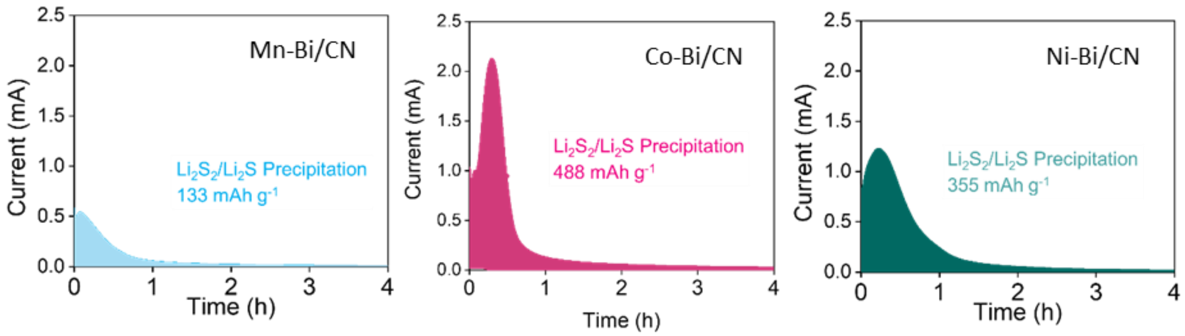

Figure S40. Potentiostatic discharge curves on Mn-Bi/CN, Co-Bi/CN, and Ni-Bi/CN electrodes to study the Li_2S_2/Li_2S nucleation kinetics. The Li_2S_2/Li_2S nucleation kinetics after galvanostatic discharge to 2.06 V at a relatively high rate (2.56 mA cm^{-2}) were analyzed by conducting potentiostatic deposition at 2.05 V using a TM-Bi/CN cathode and a Li anode using an electrolyte containing 0.5 M Li_2S_6 and 1 M LiTFSI in a 1:1 (v/v) mixture of DOL and DME (see additional details in the Experimental Section). Among the tested catalysts, Co-Bi/CN exhibited a particularly sharp nucleation peak, with the highest deposition amount (488 mAh g^{-1}), confirming its superior catalytic activity for sulfide precipitation from the polysulfide solution.

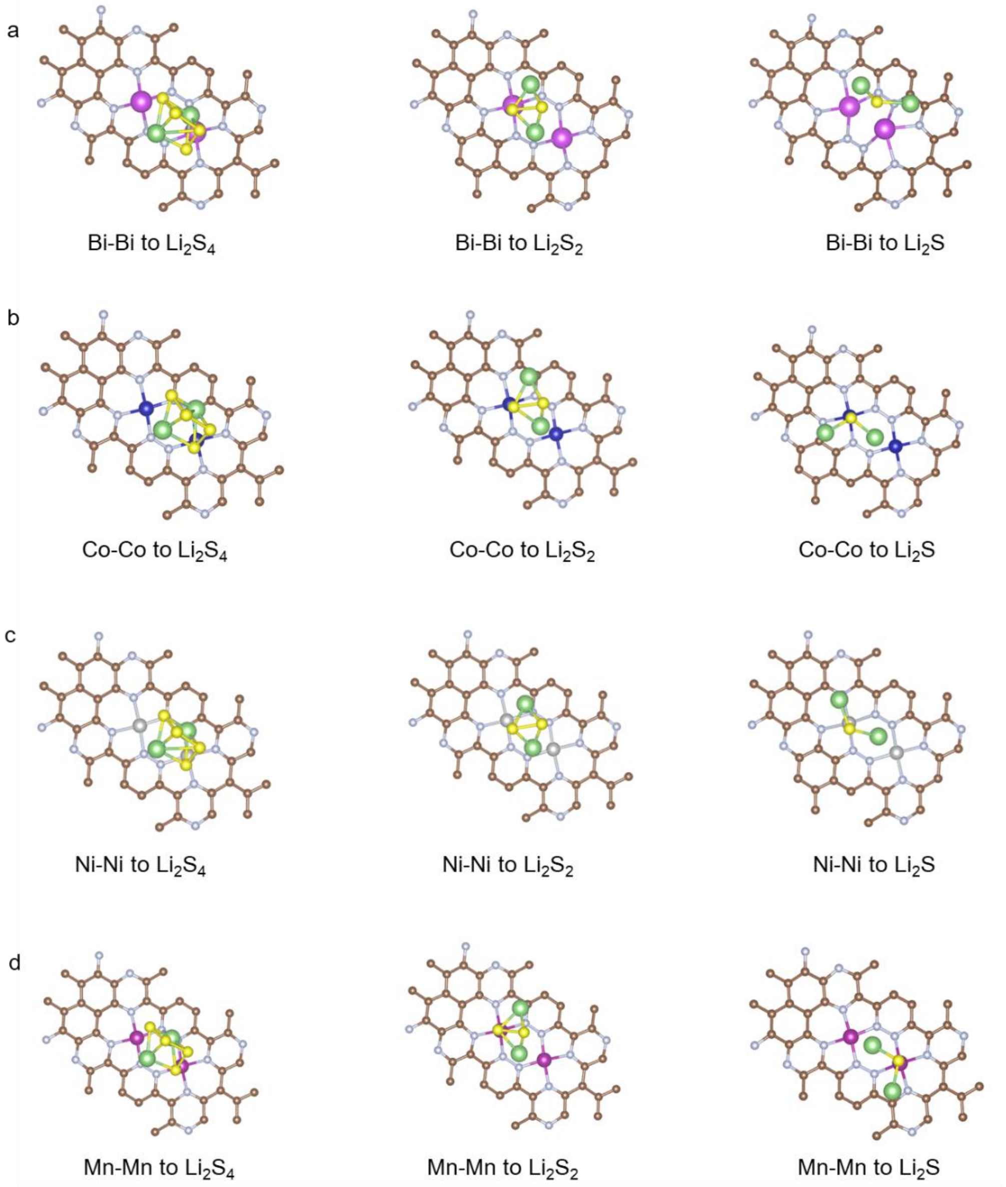

Figure S41. Top views of the Li_2S_4, Li_2S_2, and Li_2S adsorption on Bi-Bi/CN, Co-Co/CN, Ni-Ni/CN, and Mn-Mn/CN.

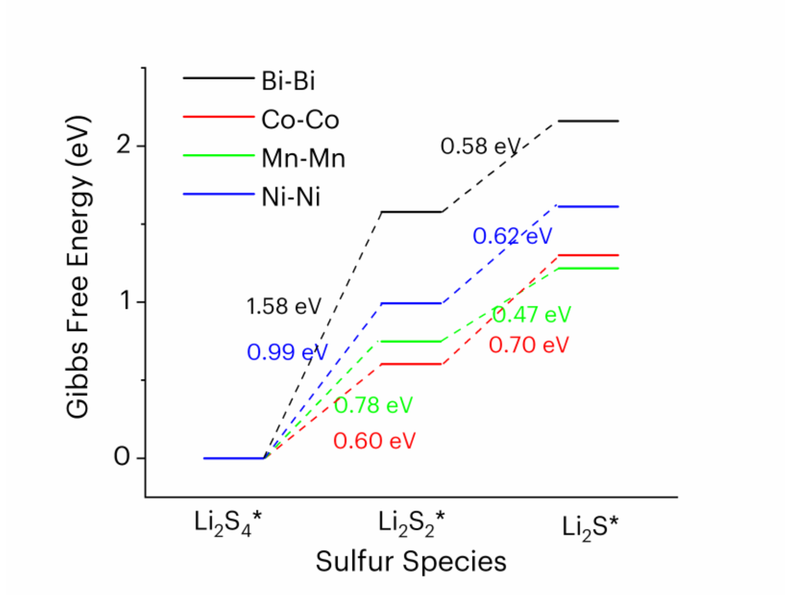

Figure S42. Gibbs free energy of polysulfides converting to Li_2S on the surface of Bi-Bi/CN, Co-Co/CN, Ni-Ni/CN, and Mn-Mn/CN.

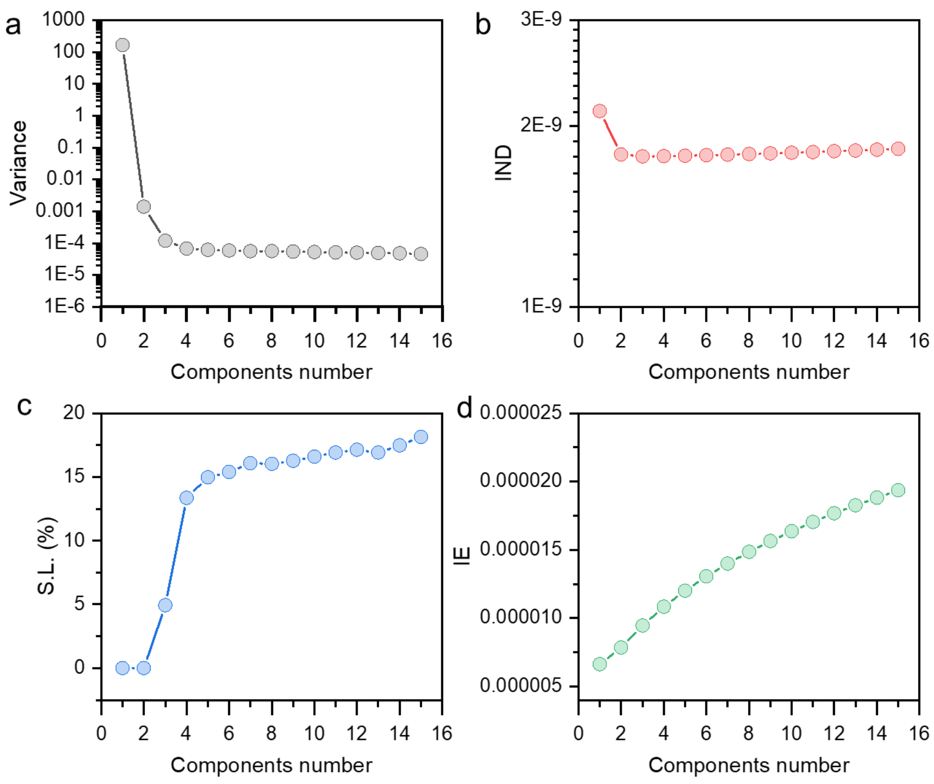

Figure S43. (a) Scree plot. (b) Factor indicator function plot. (c) Vogt–Mizaikoff F-test plot. (d) Embedded error function plot for Co/CN and Co-Bi/CN XANES spectra set.

Table S1. Atomic quantification EDS results from Figures S2-S5.

Samples / Relative Content	TM (%)	Bi (%)
Mn-Bi/CN	0.55	0.46
Co-Bi/CN	0.56	0.47
Ni-Bi/CN	0.41	0.25

Table S2. Concentrations of metal ions in various samples measured by inductively coupled plasma mass spectrometry (ICP-MS).

Samples / Relative Content	TM Concentration (%)	RSD (%)	Bi Concentration (%)	RSD (%)
Mn-Bi/CN	0.468	2.74	0.551	0.63
Co-Bi/CN	0.517	8.64	0.622	3.97
Ni-Bi/CN	0.541	5.02	0.680	2.62

RSD: Relative Standard Deviation

Table S3. Structural parameters extracted from 1st shell Fourier analysis

Name	Bonding	R (Å)	R error (Å)	N	σ^2	σ^2 error
Mn-Bi/CN	Mn-N	2.1918	0.0408	4	0.0069	0.0028
Co-Bi/CN	Co-N	1.9634	0.0138	4	0.0101	0.0023
Ni-Bi/CN	Ni-N	1.8726	0.0327	4	0.0077	0.0020

Table S4. Quantitative analysis of discharge capacities Q2/Q1 ratios for various sulfur cathodes

Samples	Q2/Q1
**Mn-Bi/CN/S**	2.23
**Co-Bi/CN/S**	2.97
**Ni-Bi/CN/S**	2.98
**Co/CN/S**	2.42
**Bi/CN/S**	2.37
**SP/S**	2.15
**CN/S**	1.31

Table S5. Comparison of rate capacity and capacity decay per cycle at 1C of metal elements in previously reported SACs and DACs, and Co-Bi/CN/S.

Catalyst Type	Metal Element(s)	Support	Sulfur Loading	High Rate Capacity ($mAh\ g^{-1}$)	Capacity Decay per cycle at 1C	Reference
DAC	Fe–Co	Hollow carbon	1.2	841 at 2C	0.018% over 1000	[2]
DAC	Co–Cu	C_3N_4	1.0	835 at 2C	0.025% over 1200	[3]
DAC	Fe–V	Carbon nanosheets	1.0–1.2	930 at 2C<br>834 at 4C	0.033% over 1000	[4]
SAC	Co	C_2N	1.27	880 at 3C	0.010% over 1000	[5]
SAC	Co	N-doped graphene	2	790 at 2C	0.053% over 500	[6]
DAC	Pt-Cu	N-doped graphene	1.5	810 at 2C	0.053% over 500	[7]
SAC	Co	P-doped CN	1.27	850 at 3C	0.056% over 600	[8]
SAC	Ni	S-doped CN	1.0-1.5	774 at 3C	0.070 % over 1200	[9]
DAC	Co-Bi	CN	1.2	906 at 3C	0.014% over 1000	This work

Table S6. Number of electrons transferred by Li_2S_4 before and after adsorption on the Co-Bi catalyst surface.

	Before Adsorption	After Adsorption	Difference
Li	2.120076	2.124649	0.00457
	2.119417	2.12021	7.93E-4
S	6.709827	6.672292	0.03753
	6.707352	6.716957	0.0096

References

[1] A. Martini, S. A. Guda, A. A. Guda, G. Smolentsev, A. Algasov, O. Usoltsev, M. A. Soldatov, A. Bugaev, Yu. Rusalev, C. Lamberti, A. V. Soldatov, *Computer Physics Communications* **2020**, *250*, 107064.

[2] X. Sun, Y. Qiu, B. Jiang, Z. Chen, C. Zhao, H. Zhou, L. Yang, L. Fan, Y. Zhang, N. Zhang, *Nat Commun* **2023**, *14*, 291.

[3] Y. Liu, Z. Hu, F. Wu, L. Li, R. Chen, *Advanced Materials* **n.d.**, *n/a*, 2506839.

[4] L. Yang, Y. Pan, Z. Zhou, Y. Zhang, J. Xu, C. Ma, Y. Zhang, J. Wang, W. Qiao, L. Ling, *ACS Nano* **2023**, *17*, 17405.

[5] D. Yang, J. Wang, C. Lou, M. Li, C. Zhang, A. Ramon, C. Li, M. Tang, G. Henkelman, M. Xu, J. Li, J. Llorca, J. Arbiol, D. Mitlin, G. Zhou, A. Cabot, *ACS Energy Lett.* **2024**, *9*, 2083.

[6] Z. Du, X. Chen, W. Hu, C. Chuang, S. Xie, A. Hu, W. Yan, X. Kong, X. Wu, H. Ji, L.-J. Wan, *J. Am. Chem. Soc.* **2019**, *141*, 3977.

[7] S. Xie, X. Chen, L. Wang, G. Zhang, H. Lv, G. Cai, Y.-R. Lu, T.-S. Chan, J. Zhang, J. Dong, H. Jin, X. Kong, J. Lu, S. Jin, X. Wu, H. Ji, *eScience* **2024**, *4*, 100222.

[8] Y. Li, Z. Chen, X.-Y. Zhong, T. Mei, Z. Li, L. Yue, J.-L. Yang, H. J. Fan, M. Xu, *Advanced Functional Materials* **2025**, *35*, 2412279.

[9] F. Zhang, Z. Tang, T. Zhang, H. Xiao, H. Zhuang, P. Han, L. Zheng, L. Jiang, Q. Gao, *Angewandte Chemie International Edition* **2025**, *64*, e202418749.
